# Assessing Cytotoxicity, Proteolytic Stability, and Selectivity of Antimicrobial Peptides: Implications for Orthopedic Applications

**DOI:** 10.3390/ijms252413241

**Published:** 2024-12-10

**Authors:** Davide Campoccia, Giulia Bottau, Andrea De Donno, Gloria Bua, Stefano Ravaioli, Eleonora Capponi, Giovanna Sotgiu, Chiara Bellotti, Silvia Costantini, Carla Renata Arciola

**Affiliations:** 1 Laboratorio di Patologia delle Infezioni Associate all’Impianto, IRCCS Istituto Ortopedico Rizzoli, Via di Barbiano 1/10, 40136 Bologna, Italy; giulia.bottau@ior.it (G.B.); andrea.dedonno@ior.it (A.D.D.); gloria.bua@ior.it (G.B.); stefano.ravaioli@ior.it (S.R.); eleonora.capponi@ior.it (E.C.); 2 Institute for Organic Synthesis and Photoreactivity (ISOF), National Research Council, Via Gobetti 101, 40129 Bologna, Italy; giovanna.sotgiu@isof.cnr.it; 3 Osteoncology, Bone and Soft Tissue Sarcomas and Innovative Therapies Unit, IRCCS Istituto Ortopedico Rizzoli, Via di Barbiano 1/10, 40136 Bologna, Italy; chiara.bellotti@ior.it; 4 Department of Medical and Surgical Sciences (DIMEC), University of Bologna, Via San Giacomo 14, 40126 Bologna, Italy; silvia.costantini4@studio.unibo.it; 5 Laboratory of Immunorheumatology and Tissue Regeneration, Laboratory of Pathology of Implant Infections, IRCCS Istituto Ortopedico Rizzoli, Via di Barbiano 1/10, 40136 Bologna, Italy

**Keywords:** antimicrobial peptides, AMPs, implant orthopedic infections, cytotoxicity, cytocompatibility, selectivity index, proteolytic stability, MG63, L929, hMSCs

## Abstract

In orthopedics, the use of anti-infective biomaterials is considered the most promising strategy to contrast the bacterial contamination of implant surfaces and reduce the infection rate. KSL, KSL-W, and Dadapin-1 are three antimicrobial peptides (AMPs) that possess significant antibacterial properties, making them promising candidates for producing anti-infective biomaterials not based on antibiotics. To fully assess their true potential, this study explores in detail their cytocompatibility on human osteoblast-like MG63 cells, murine fibroblastoid L929 cells, and hMSCs. To this end, the cytotoxicity of the AMPs in terms of IC_50_ was tested over a range of concentrations of 450–0.22 µg/mL using the ATP bioluminescence assay. The tests were performed both in the presence and absence of bovine serum to assess the effects of serum components on peptide stability. IC_50_ values obtained under the most stringent conditions were used to extrapolate the selectivity index (S.I.) toward salient bacterial species. In medium containing serum, all AMPs exhibited minimal to no cytotoxicity, with IC_50_ values exceeding 100 µg/mL. Dadapin-1 was the peptide that exhibited the lowest cytotoxicity, KSL-W exhibited the highest stability, and KSL exhibited the highest selectivity. Overall, these findings highlight the potential of these AMPs for the future production of anti-infective materials.

## 1. Introduction

In orthopedics, implant infections are primarily caused by staphylococci and other biofilm-forming bacterial species. Biofilm infections are arduous to eradicate via conventional antibiotics and insidiously elusive to immune defenses [1,2]. Indeed, once bacteria adhere to a biomaterial surface and form a biofilm, they become tolerant to antibiotics [3]. This tolerance is associated with the low sensitivity of slowly growing dormant bacteria encased in biofilms, known as bacterial persisters [4]. Bacterial persisters and intracellular bacteria are known to survive antibiotic therapies [2,4].

The alarming rate of multidrug resistance in pathogens causing biomaterial-associated infections poses additional concerns about the future possibilities for treatment. 

In this complex scenario, anti-infective biomaterials have emerged as one of the key strategies to deal with this complex phenomenon [5]. Therefore, research studies are embracing multifaceted approaches that include strengthening the immune defenses against biofilms and promoting the biocompatibility and osseointegration of implant materials, since these measures contribute toward thwarting bacterial colonization and infection.

AMPs are at the forefront of research on advanced anti-infective biomaterial systems. Natural AMPs have inspired the design of new synthetic AMPs with improved antimicrobial and biological properties [6,7]. Moreover, AMPs can be identified or, alternatively, designed and optimized to achieve additional biological functionalities. Interestingly, apart from their antibacterial activity, single AMP molecules have been shown to possess additional unexpected biological activities. These include the neutralization of bacterial virulence factors (anti-endotoxic effect) [8]; immunomodulatory effects on leukocytes [8]; induction of cell differentiation [9] and, interestingly, cell adhesion and healing properties [10]. As of March 2024, Cresti et al. [11] reported the descriptions of over 3100 natural AMPs after consulting the Antimicrobial Peptide Database (APD3) [12], of which, 383 were from bacteria, 5 were from archaea, 8 were from protists, 29 were from fungi, 250 were from plants, and 2463 were from animals. Nonetheless, a growing number of new antibacterial peptides are represented by synthetic AMPs. Indeed, new artificial AMP sequences can rapidly be generated by identifying and improving the sequences of more promising natural AMPs by site-directed mutations, de novo designs, template-based designs, and chemical and motif-based modifications. Computer-assisted designs are increasingly becoming the strategies of choice for identifying new, better-performing AMPs, either based on simple statistical modeling or the most advanced and sophisticated approaches by neural networks [13], deep learning [14], and machine learning AI-driven discovery [15]. 

The driving reason for designing new artificial molecules arises from decennial investigations conducted on thousands of natural peptides, which have shown that, in front of many interesting and precious properties, AMPs tend to exhibit certain general limitations. Among the appealing characteristics of AMPs is that their mechanisms of action differ from those of conventional antibiotics, thus they retain powerful bactericidal effects, even on multi-drug resistant (MDR) strains. Numerous AMP molecules are capable of synergistic effects with common antibiotics. Moreover, some have been reported to restore the bactericidal activity of antibiotics on resistant strains [7]. 

Differing from most antibiotics, they can often destroy even bacterial cells that are dormant or live protected within biofilms or host cells, following internalization. Additionally, many AMPs have been found to possess anticancer and immunomodulatory effects. Furthermore, although several bacterial mechanisms of resistance have already been unveiled [16], AMPs rely on antibacterial mechanisms that are generally less prone to stimulate the emergence of drug-resistant strains. On the other hand, the shortcomings mainly concern intrinsic characteristics typical of peptides, such as low molecular stability, susceptibility to different physical and chemical factors (pH, light, oxidation, proteolytic enzymes), short half-lives, and, in many cases, lack of selectivity with consequent hemolysis and toxicity to host cells. Antimicrobial peptide discovery can be directed to mining the best-performing peptides, further improving their structures, or designing new molecules, in order to maximize the bactericidal and antibiofilm activities, improve molecular stability, enhance selectivity, and minimize any hemolytic and cytotoxic unwished effects on mammalian cells. 

The selection of improved peptide sequences helps to minimize certain shortcomings of current AMPs, such as thermal instability, low selectivity, vulnerability to the enzymic action of proteases, or the inactivation following the interaction/complexation with components of the physiologic fluids or defense mechanisms developed by bacteria.

Several AMPs are amphipathic cationic peptides and exhibit broad-spectrum antimicrobial activity against Gram-positive and Gram-negative bacteria, fungi, and viruses. Some AMPs are known to display antimicrobial activity against bacteria that are resistant to antibiotics and reside within biofilms. Their mechanisms of action involve interacting with constituents of the bacterial cell envelope, with consequent depolarization and destabilization, and finally, disruption of the bacterial plasma membrane, causing bacterial cell death. However, other AMPs exploit different mechanisms of action and cellular targets, such as enzymatic activities that induce the lysis of bacterial cell walls [7]. Notably, some AMPs are well-suited for incorporation into drug delivery systems that are suitable for implant coatings [6].

In recent years, diverse papers have underscored the important role that AMPs could play in the battle against implant-related infections in orthopedic surgery [17,18,19,20]. As early as 2021, searching for valuable AMP molecules for possible applications in orthopedics, our group conducted an in-depth systematic review of known short-chain AMPs, mainly based on the Database of Antimicrobial Activity and Structure of Peptides (DBAASP) [21]. The quest included all monomeric AMPs with lengths between 7 and 13 amino acids. Taking into account all the preliminary information available, and after refining our research, we identified KSL and KSL-W as the two peptides with the greatest potential for orthopedic applications. Both KSL and its derivative KSL-W have already been demonstrated to possess potent activity against oral bacteria [22,23,24,25]. Along with these two short peptides, our interest was also in a 23-amino acid peptide, Dadapin-1, which was recently developed and described by Rončević et al. (2019) [26]. The antibacterial properties of all three of these AMPs have recently been thoroughly tested on bacterial isolates from clinical orthopedic infections [27,28]. 

Unfortunately, there is a limited number of studies that have exhaustively investigated the toxicity of antimicrobial peptides on mammalian cells or assessed their level of selectivity in terms of IC_50_ and selectivity. As far as KSL-W is concerned, one study investigated the long-term cytotoxicity on MC3T3-E1 cells of extracts from KSL-W-loaded PLGA/chitosan composite microspheres, which were meant to be used for the treatment of oral infectious diseases as well as within bone graft substitutes for alveolar bone augmentation [29]. The authors reported no cytotoxicity affecting the growth of osteoblast MC3T3-E1 over a 5-day time period. More recently, a KSL-W@PLGA/collagen (COL)/silk fibroin (SF)/nano-hydroxyapatite (nHA) (COL/SF/nHA) scaffold via a 3D-printing technique was reported to exhibit good biocompatibility and osteoconductive properties [30]. However, to our knowledge, in the literature, no detailed information on IC_50_ and S.I. for KSL and KSL-W is available. This has to be attributed at least in part to the low level of cytotoxicity. Just for Dadapin-1, we earlier reported some experimental data on the IC_50_ found for the osteoblast-like MG63 cells [28]. 

The overall aim of this study is to explore the behaviors of the two decapeptides, KSL and KSL-W, as well as Dadapin-1, when challenged in vitro with a panel of different eukaryotic cells. After the past investigation of Dadapin-1 [28], in the present work, the characterization of the cytocompatibility is further extended, with its cytotoxicity and behavior on two new eukaryotic cell lines tested. 

Selectivity of the AMPs toward procaryotic cells is another crucial parameter when assessing the potential clinical use of new bioactive substances. Therefore, the selectivity indices of Dadapin-1, KSL, and KSL-W were estimated after assessing the level of cytotoxicity on mammalian cells. Analogous to what has been described for the inhibitory activity of undiluted bacterial culture broth, Mueller Hinton Broth (MHB) II [28], even bovine serum components present in mammalian cell culture medium are known to interfere with AMP activity. For instance, peptides can be sensitive to proteolytic degradation by serum proteases. Thus, for a more complete assessment, the cytotoxicity of the peptides was assayed on three distinct cell lines, i.e., murine fibroblast-like L929 cells, human osteoblast-like MG63, and mesenchymal stem cells (hMSCs), and tested both in the presence and absence of bovine serum.

## 2. Results

### 2.1. AMPs Cytotoxicity

#### 2.1.1. Cell Cytotoxicity

The cytotoxicity data of KSL and KSL-W were assayed on three different cell lines, respectively, the murine fibroblast-like L929 cells, the human osteoblast-like MG63 cells, and hMSCs, both in the presence and absence of bovine serum. For Dadapin-1, the cytotoxicity data concerning the effects on MG63 cells have already been published in a previous paper [28]; here, only the new data on L929 cells and hMSCs are introduced.

- Dadapin-1.

Dadapin-1 was previously found to exhibit minimal cytotoxicity on human osteoblast-like MG63 [28]. In the earlier study, at the highest concentration (450 µg/mL), Dadapin-1 caused an 18.8% reduction in cell metabolism in MG63 cells cultured in the presence of serum with respect to the reference control. Conversely, in the absence of serum supplementation in the culture medium, a 17.4% reduction was observed at a lower concentration of Dadapin-1 (225 μg/mL), suggesting a slight increase in cytotoxicity. 

**Figure 1:**
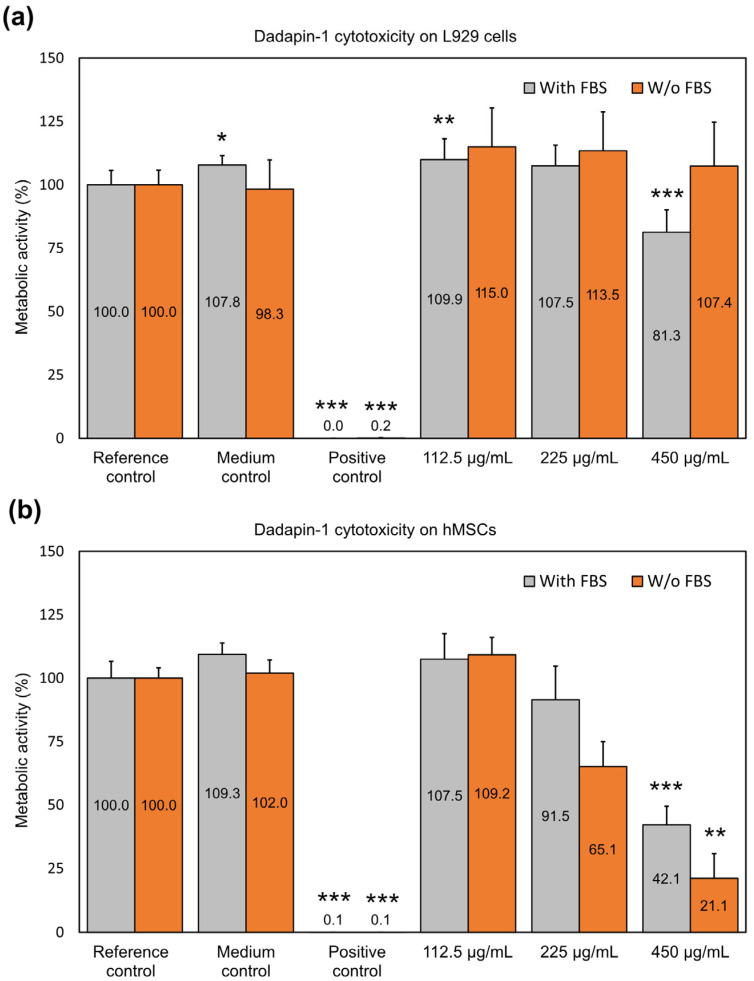
The bar graphs show the effects of Dadapin-1 on the cellular metabolism of (**a**) L929 cells and (**b**) hMSCs in the absence and presence of fetal bovine serum (FBS). Bioluminescence readings in relative luminescence units (RLUs) were normalized to the mean of the reference control set to 100%. Bars represent the mean ± S.D. of three independent experiments (*N* = 9), except for the assay performed with L929 without FBS (four independent experiments, *N* = 12). The statistically significant difference with respect to the reference control: * *p* < 0.05; ** *p* < 0.01; *** *p* < 0.001. Detailed information on the statistical analysis performed and exact *p*-values can be found in Tables S2 and S3.

In the present work, the cytotoxicological studies on Dadapin-1 were further extended. Fibroblast-like L929 cells were found to be only marginally affected by Dadapin-1 (Figure 1a). In the presence of the serum, L929 cells behaved similarly to MG63 cells, exhibiting the same susceptibility to the effects of Dadapin-1. Indeed, just a slight metabolic reduction of 18.7% was actually observed at the highest concentration of Dadapin-1 assayed. Unexpectedly, in the absence of serum, L929 cells did not exhibit significant metabolic reduction at any concentration of Dadapin-1 tested. This result was confirmed by four independent experiments (*N* = 12). For such a low level of cytotoxicity both in the presence and absence of serum, the IC_50_ could not be extrapolated.

hMSCs proved to be more susceptible to the effects of Dadapin-1 (Figure 1b). Indeed, in a medium supplemented with serum, a concentration of 450 µg/mL of Dadapin-1 caused a 57.9% reduction in cell activity, while at 225 µg/mL, the reduction was just 8.5%. On the contrary, in the absence of serum, at a concentration of 225 µg/mL was already capable of reducing cell metabolism by 34.9%. The difference in cell activity, comparing the effects in the presence and absence of serum, was 26.4% at a concentration of 225 µg/mL and 21.1% at 450 µg/mL. As earlier seen with MG63 cells, FBS appears to exert a protective effect against the cytotoxicity of Dadapin-1. Likely, as observed in the case of other peptides, Dadapin-1 is sensitive to the action of the proteases present in serum.

These results confirm no (or very low) cytotoxicity of Dadapin-1 with L929 cells and slightly more pronounced effects on hMSCs, starting at 450 μg/mL with the serum and 250 μg/mL without serum. The trend of susceptibility to Dadapin-1 for the different cell lines was as follows: hMSCs > MG63 cells = L929 cells with FBS and hMSCs > MG63 cells > L929 without FBS.

It may be noticed that, as a trend, at sub-cytotoxic concentrations of 225 and 112.5 μg/mL, Dadapin-1 appears to stimulate the cell metabolism of L929 fibroblasts both in the presence and absence of serum. However, statistical significance is fully achieved only at a concentration of 112.5 μg/mL with FBS. At a concentration of 112.5 μg/mL, a similar effect is observed even on hMSCs, nonetheless, the increase in metabolism did not reach statistical significance.

- KSL.

**Figure 2:**
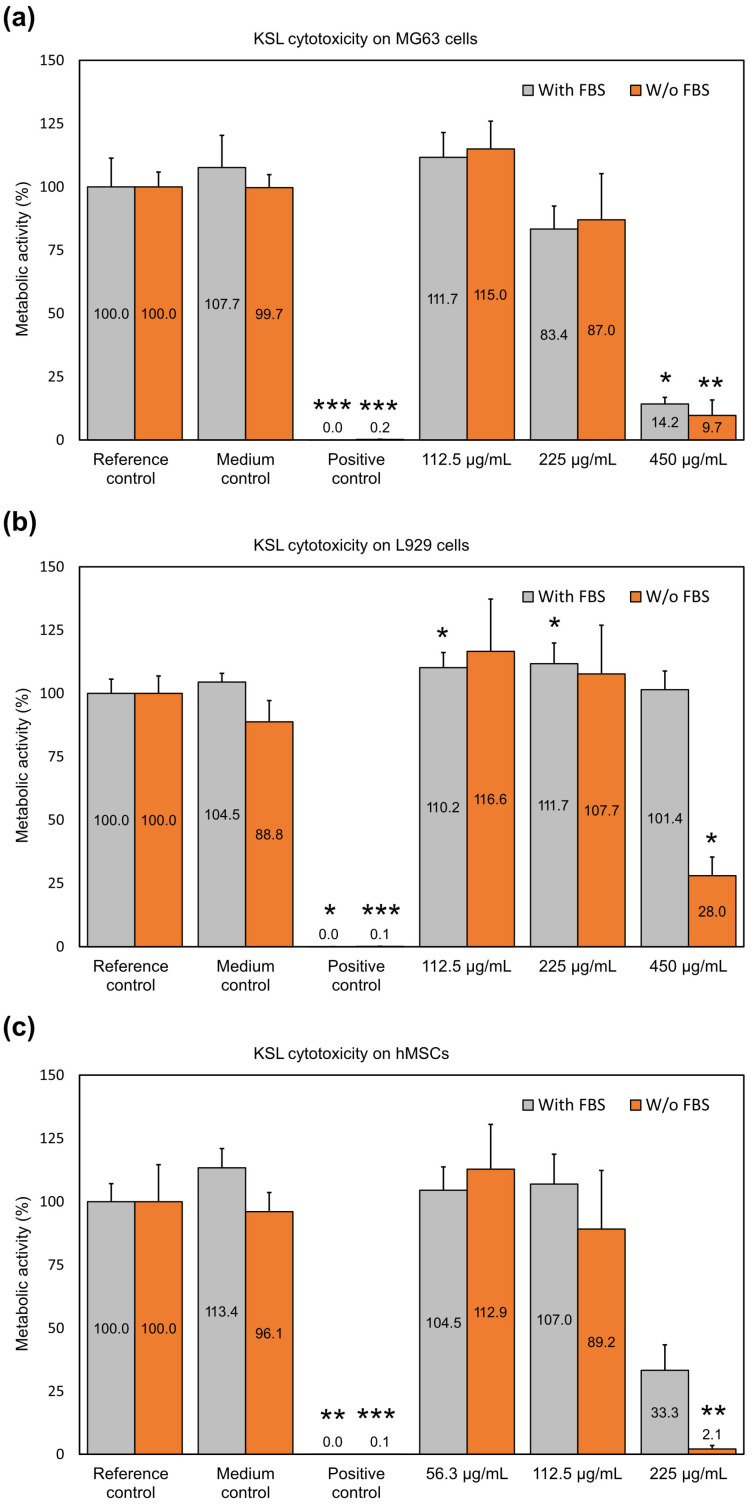
The bar graphs show the effects of KSL on the cellular metabolism of (**a**) MG63 cells, (**b**) L929 cells, and (**c**) hMSCs in the absence and presence of FBS. Bioluminescence readings in relative luminescence units (RLUs) were normalized to the mean of the reference control set to 100%. Bars in the graph represent the mean ± S.D. of three independent experiments (*N* = 9), except for the assay performed with MG63 and hMSCs without FBS (four independent experiments, *N* = 12). The statistically significant difference with respect to the Reference control: * *p* < 0.05; ** *p* < 0.01; *** *p* < 0.001. Detailed information on the statistical analysis performed and exact *p*-values can be found in Tables S2 and S3.

Regarding KSL (Figure 2a), mild cytotoxicity was observed on MG63 cells starting at 225 µg/mL, when very slight reductions in cell metabolism of 16.6% and 13.0% were observed with FBS and without FBS, respectively. At 450 µg/mL, KSL was overtly cytotoxic for osteoblast-like cells. No significant difference emerged from the treatment comparison with and without serum.

Conversely, in the presence of serum, the metabolism of L929 cells was not affected at any of the tested KSL concentrations (Figure 2b). Only in the absence of serum and at the highest concentration tested was the cell activity significantly altered by the peptide, with a reduction of up to 78.0% in metabolism observed. Thus, the presence of FBS was able to influence the cell behavior, but only at the highest concentration of the peptide.

Again, hMSCs proved to be the most sensitive cell line to the test peptide (Figure 2c). A significant reduction in cell metabolism was noticeable at 225 µg/mL both in the presence and absence of FBS. At this concentration of the peptide, a 31.2% difference in metabolic activity was observed, depending on the use of serum supplementation of the medium. The trend of susceptibility to KSL for the different cell lines was as follows: hMSCs > MG63 cells > L929 cells. 

Overall, concentrations of up to 112.5 µg of KSL/mL appear to be safe even for the most sensitive cell line used in the present investigation. However, it remains difficult to explain the reason why the presence/absence of serum did not always produce different effects on cellular metabolism, as one would expect. In fact, regardless of the cell line tested, the activity of serum proteases should always result in a clear reduction of cytotoxicity, causing a shift in the final concentration of active peptide in the solution.

Even for KSL, as previously reported for Dadapin-1, sub-cytotoxic concentrations of the peptide were found to stimulate cell metabolism. At a concentration of 112.5 µg/mL, KSL stimulated and increased metabolism in MG63 cells and L929 cells in the presence and absence of serum. With hMSCs, sub-cytotoxic concentrations of KSL caused a slight increase in cell metabolism, but the difference never met statistical significance.

- KSL-W.

**Figure 3:**
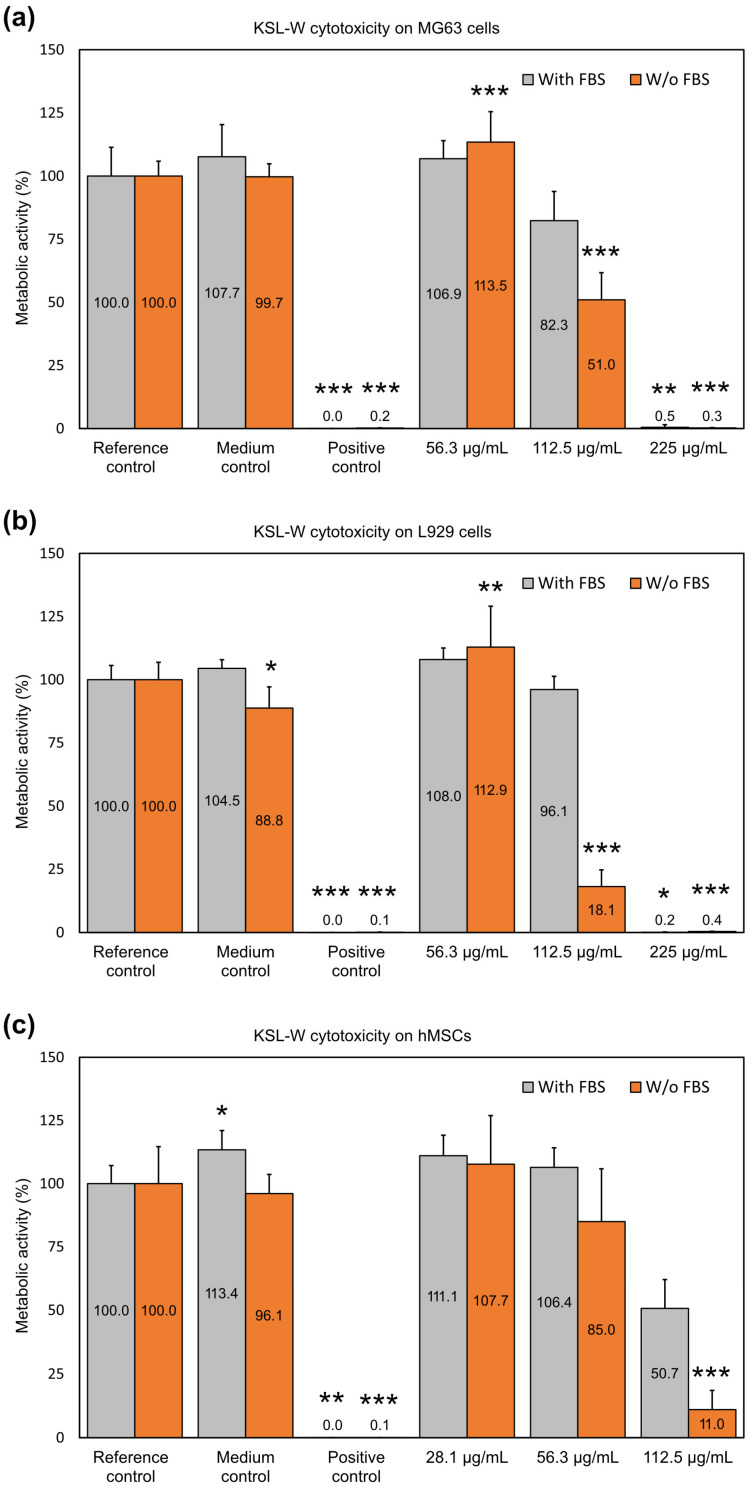
The bar graphs show the effects of KSL-W on the cellular metabolism of (**a**) MG63 cells, (**b**) L929 cells, and (**c**) hMSCs in the absence and presence of FBS. Bioluminescence readings in relative luminescence units (RLUs) were normalized to the mean of the reference control set to 100%. Bars in the graph represent the mean ± S.D. of three independent experiments (*N* = 9), except for the assay performed with MG63 and hMSCs without FBS (four independent experiments, *N* = 12). The statistically significant difference with respect to the Reference control: * *p* < 0.05; ** *p* < 0.01; *** *p* < 0.001. Detailed information on the statistical analysis performed and exact *p*-values can be found in Tables S2 and S3.

When KSL-W was assayed on MG63 cells, the peptide was found to be non-cytotoxic or just slightly cytotoxic, up to a concentration of 112.5 µg/mL in the presence of serum and up to 56.3 µg/mL in its absence (Figure 3a). At 112.5 µg/mL, the reductions in cell activity observed with and without FBS were 17.7% and 49.0%, respectively. The absence of serum, therefore, resulted in a 31.3% drop in cell activity.

Similarly to osteoblast-like cells, L929 cells indicated that KSL-W was not cytotoxic up to the concentration of 112.5 µg/mL in the presence of serum and up to 56.3 µg/mL in its absence (Figure 3b). Nonetheless, in the absence of serum, the concentration of 112.5 µg/mL caused an 81.9% reduction in cell metabolism. Thus, the difference in cell metabolism made by the presence or absence of serum accounted for 78.0%. 

As also observed with the other two peptides, hMSCs were the cells exhibiting the most pronounced effects. KSL-W was found to be non-cytotoxic or just mildly cytotoxic, up to a concentration of 56.3 µg/mL when tested in the presence of serum (Figure 3c). At 112.5 µg/mL, the difference in metabolic activity between treatments performed with and without serum was 37.7%. The trend of susceptibility to KSL-W across the different cell lines was as follows: hMSCs > L929 cells > MG63 cells with FBS and hMSCs > MG63 cells > L929 cells without FBS. 

With all three cell lines, the presence of serum clearly attenuated the reduction in metabolic activity caused by the peptide. Our findings suggest that hMSCs are the cells with the most vulnerable phenotypes to the cytotoxic activity of cationic AMPs. Among the three peptides assayed for cytotoxicity, KSL-W appeared to exhibit the strongest effect on eukaryotic cells. When uniquely considering the experiments conducted in the presence of serum, 112.5 µg/mL can be considered a safe concentration for MG63 cells and L929 cells. Nonetheless, if including hMSCs and testing conditions without FBS, the safe concentration drops to 56.3 µg/mL, in which case, a mild decrease in metabolic activity (15.0%) is observed only with hMSCs in the absence of FBS.

The increased metabolism trend at sub-cytotoxic concentrations was observed even in the case of KSL-W. With osteoblast-like MG63 cells, the 56.3 µg/mL concentration was found to increase the cell activity both in the presence and absence of the serum, but with the serum, the difference did not reach statistical significance. 

#### 2.1.2. Assessment of IC_50_ Value

**Figure 4:**
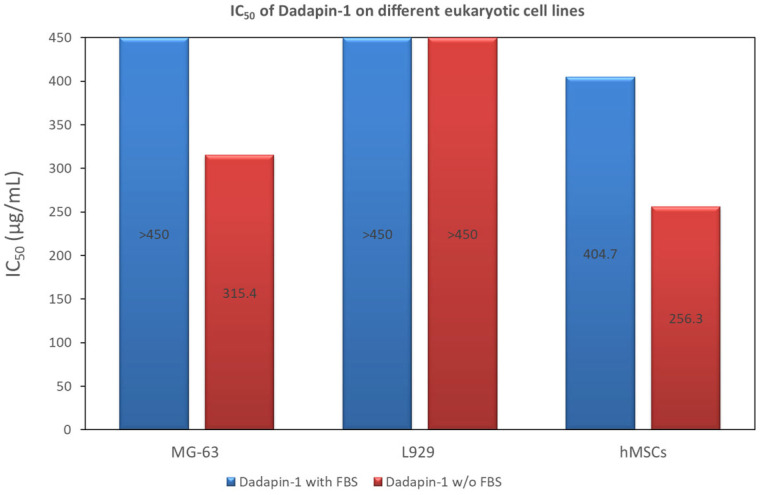
The bar graph reports the calculated IC_50_ values of Dadapin-1 when tested on MG63 cells, L929 cells, and hMSCs, in the presence (blue bars) and absence of FBS (red bars). Plotted values for MG63 cells refer to our previously published work [18]. Conversely, the values for L929 cells and hMSCs are newly introduced by the present investigation. It may be noticed that Dadapin-1 exhibits no or very low cytotoxicity. For both MG63 cells and hMSCs, the level of cytotoxicity increased in the absence of serum.

In our earlier work [18], Dadapin-1 exhibited the absence of cytotoxic effects up to the highest concentration of AMP tested (450 μg/mL), when assayed in the presence of serum on MG63 cells. The absence of bovine serum in the medium resulted in very mild cytotoxicity, with an IC_50_ value of 315.4 μg/mL. The overall low profile of cytotoxicity of Dadapin-1 was further confirmed in this extended investigation, where this AMP was also tested on fibroblast-like L929 cells and hMSCs (Figure 4). L929 cells were found to be less susceptible to the effects of Dadapin-1, and it was impossible to calculate an IC_50_ value in the range of concentrations tested even when the peptide was assayed in the absence of FBS. hMSCs were found to be more susceptible to the peptide effects, and it was possible to calculate an IC_50_ value even in the presence of serum. Nonetheless, the level of cytotoxicity remained marginal with IC_50_ values of 404.7 and 256.3 μg/mL, respectively, in the presence and absence of serum.

**Figure 5:**
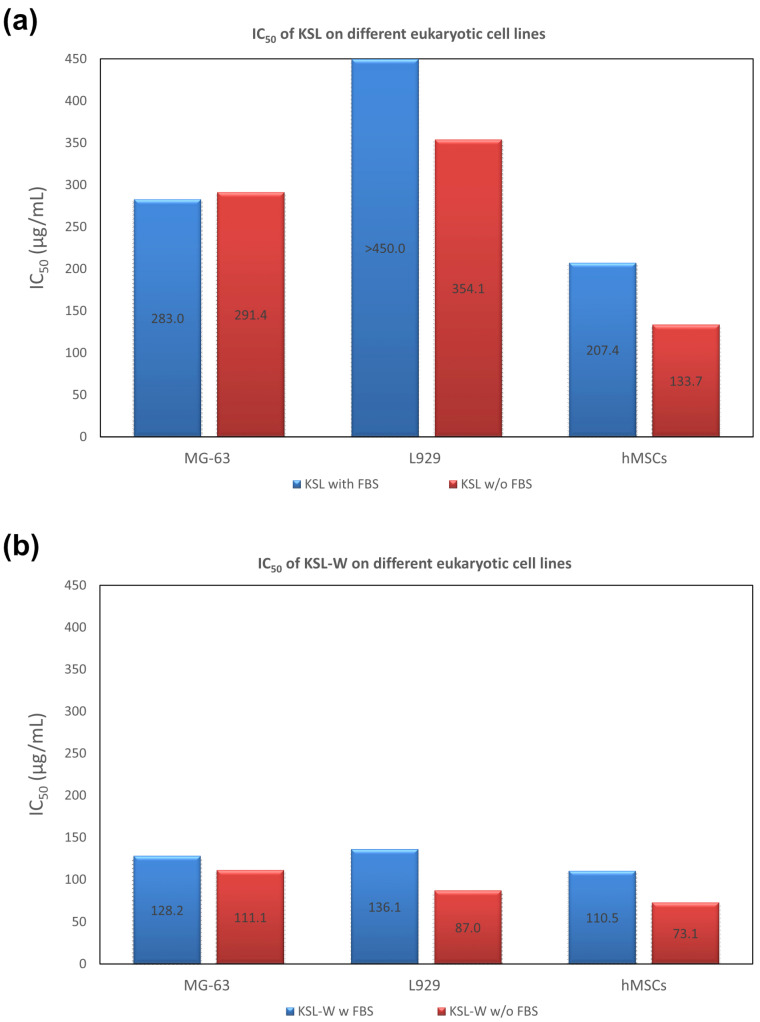
The bar graph presents the calculated IC_50_ values of (**a**) KSL and (**b**) KSL-W when tested on MG63 cells, L929 cells, and hMSCs, in the presence (blue bars) and absence of FBS (red bars). Confidence intervals for IC_50_ values can be found in Table S4.

KSL and KSL-W showed more pronounced effects on mammalian cells. As far as the former peptide is concerned, it is interesting to note the following: (i) KSL generally showed slightly increased cytotoxicity compared to Dadapin-1; (ii) the susceptibility of the cells to the effects of the peptide followed this trend: hMSCs> MG63 cells ≥ L929 cells; (iii) surprisingly, while L929 cells and hMSCs were more affected by KSL when cytotoxicity was tested in the absence of FBS, this did not hold true for MG63 cells, where IC_50_ values were roughly the same with or without serum (see Figure 5a).

Interestingly, KSL-W exhibited a more pronounced effect on mammalian cells (Figure 5b). In the presence of FBS, IC_50_ values fell in a rather restricted range: from 136.1 μg/mL to 110.5 μg/mL, respectively, for L929 cells and hMSCs. As per the other two AMPS, the trend of susceptibility to the cytotoxic effects was hMSCs > MG63 cells > L929 cells, even if differences in IC_50_ were slight. In the absence of FBS, IC_50_ values always decreased, confirming the protective effect of serum. Nonetheless, with MG63 cells, the cytotoxicity variation in the presence and absence of FBS was marginal (with an IC_50_ value of 128.2 vs. 111.1 μg/mL, respectively).

#### 2.1.3. AMP Effects on the Cytomorphology of MG63 Cells

**Figure 6:**
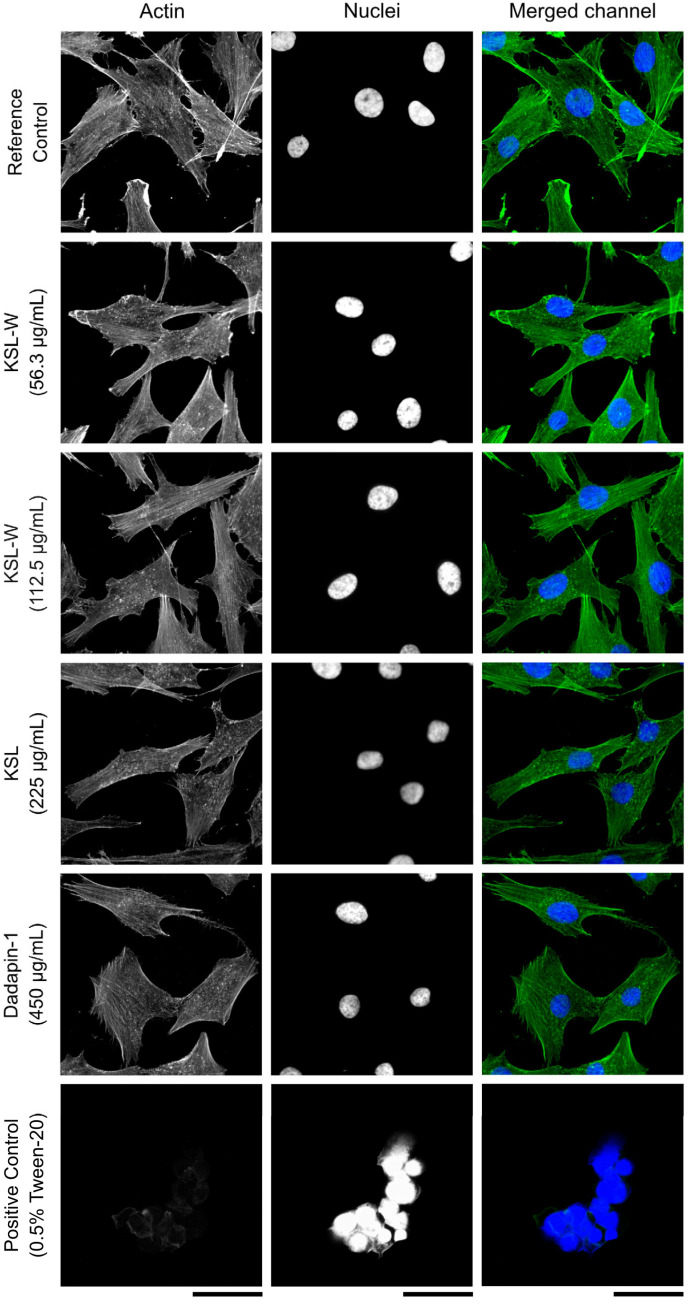
Representative images of MG63 cells treated for 24 h with the indicated substances. The nuclei staining (Hoechst 333432) is shown in blue, and the actin cytoskeleton staining (Phalloidin-FITC) is shown in green. Bottom scale bars: 50 µm.

The actin cytoskeleton plays a central role in many aspects of cell physiology, first of all, by maintaining cell shape and structure. In order to detect subtle changes that could occur in cellular morphology due to possible toxicological effects of the investigated AMPs, we analyzed the integrity of the actin network of MG-63 cells treated with the tested substances at concentrations that showed mild reductions in terms of metabolic activity. In Figure 6, representative micrographs illustrate the effects on osteoblast-like cells of Dadapin-1 (at a concentration of 450 µg/mL), KSL (at a concentration of 225 µg/mL), and KSL-W (at concentrations of 56.3 and 112.5 µg/mL). After 24 h of treatment, MG63 cells overall maintained typical fibroblast-like morphology, which was observed for the reference control in the absence of peptides. Regularly organized F-actin bundles were clearly visible along the cell axis in all conditions. Conversely, in the positive control, where cells were treated with the detergent Tween-20 (a known cytotoxic chemical active on cell membranes), cell morphology was completely lost and only a faint disorganized fluorescent signal of the actin cytoskeleton was detected.

#### 2.1.4. Selectivity of Antibacterial Activity

The selectivity of the antimicrobial activity of the test AMPs was investigated in depth using the selectivity index (S.I. = IC_50_/MIC). For greater cautiousness, I.S. was estimated using the lowest obtained IC_50_ values (i.e., those referring to hMSCs). For MICs, we refer to the values reported in [16], as follows: when the obtained MIC values spanned across two dilutions, the selectivity estimation was extrapolated using the higher of the two MIC values.

I.S. values were calculated for four different strains belonging to the most representative Gram-positive and Gram-negative pathogens causative of implant-related orthopedic infections: *S. aureus* (ATCC25923); *S. epidermidis* (*cra*4034); *E. coli* (*cra*4038); and *P. aeruginosa* (*cra*3840). For prompt reference, the values of the MICs in undiluted and diluted Müller Hinton Broth II (MHB II) that were used to extrapolate the I.S. are displayed in Table 1.

The I.S. values that were obtained, considering the MICs in both diluted and undiluted broth, are presented in detail in Table 2. In the same table, I.S. values based on IC_50_ values for hMSCs cultured with and without FBS are displayed.

- S.I. under the most challenging conditions (IC_50_ of HMSCs w/o FBS and MIC in undiluted MHB II).

Figure S1 presents the I.S values extrapolated for KSL and KSL-W under the most stringent conditions, i.e., using MICs from tests performed with undiluted broth and IC_50_ values obtained for hMSCs in the absence of serum. Under these conditions, the interference of the undiluted broth was pronounced, and MICs were much greater. It may be noticed that, generally, the two peptides tended to perform similarly and, despite the unfavorable considered conditions, showed good selectivity toward *S. epidermidis* and *E. coli*. In the case of *S. epidermidis*, KSL demonstrated a greater selectivity margin compared to KSL-W (S.I. of 34.2 vs 18.7). With *S. aureus*, selectivity values of KSL and KSL-W appeared much lower (4.3 and 4.7, respectively). However, the lowest selectivity was observed with *P. aeruginosa*, with KSL and KSL-W exhibiting values as low as 1.1 and 2.3, respectively.

Dadapin-1 is not plotted in the Figure S1 graph because its MIC values for *S. aureus*, *E. coli,* and *P. aeruginosa* were not available with undiluted media as they exceeded concentrations of 500 μg/mL. Uniquely for the *S. epidermidis cra*4034 strain, which exhibited a MIC of 250–500 μg/mL, the I.S. value was 0.5, indicating a significant loss of antibacterial activity for this peptide in undiluted MHB II.

- Trustworthy S.I. values (IC_50_ Value of hMSCs w/o FBS and MIC in 20% MHB II).

The level of selectivity consistently increased when the extrapolation of S.I. values was performed using MICs obtained from tests performed in diluted MHB II, i.e., under reliable conditions, with reduced interference from the medium affecting the antibacterial activities of peptides. Figure S2 reports the selectivity values obtained with this approach. It may be noticed that, unexpectedly, Dadapin-1 and KSL-W appear to behave similarly in terms of selectivity, with KSL-W having just a slight advantage. This advantage was much more pronounced in the case of *E. coli* (8.2 for Dadapin-1 vs. 18.7 for KSL-W). Surprisingly, KSL was discovered to exhibit completely different behavior and outperform the other two peptides, showing remarkable selectivity. For instance, with *S. epidermidis*, the S.I. value of KSL was 136.4 vs. 37.5 for KSL-W and 32.8 for Dadapin-1 and, with *P. aeruginosa*, it was 34.2 vs. 9.4 for KSL-W and 8.2 for Dadapin-1. It must be emphasized that these values were all calculated when applying restrictive conditions, i.e., when considering the IC_50_ of the most susceptible cell line in the absence of serum.

- S.I. under the most favorable conditions (IC_50_ of hMSCs with FBS and MIC in diluted MHB II).

It should be noted that, when one considers IC_50_ in the presence of serum and MICs in 20% broth, the S.I. values of the peptides further increase. Again, Dadapin-1 and KSL-W expressed similar behavior with S.I. values generally rather close to each other: with *S. aureus*—25.9 for Dadapin-1 vs. 28.3 for KSL-W; with *S. epidermidis*—51.8 for Dadapin-1 vs. 56.7 for KSL-W; with *P. aeruginosa*—13.0 vs. 14.1. Only for *E. coli* was the S.I. pronouncedly different, i.e., more than double (13.0 for Dadapin-1 vs. 28.3 for KSL-W). Interestingly, KSL selectivity was by far superior, with I.S. ranging from 53.0 for *P. aeruginosa* and up to 211.6 for *S. epidermidis*. 

Overall, under the most favorable conditions and in the absence of MHB II interference, Dadapin-1 selectivity had the following decreasing order: *S. epidermidis* > *S. aureus* > *E. coli* = *P. aeruginosa*. Conversely, KSL and KSL-W exhibited a similar profile. For both peptides, the selectivity profile was as follows: *S. epidermidis* > *S. aureus* = *E. coli* > *P. aeruginosa*. It is interesting to note that, when referring to MICs in 100% MHB, the selectivities for KSL and KSL-W followed different trends. For KSL the profile was *S. epidermidis* > *E. coli* > *S. aureus* > *P. aeruginosa*, while for KSL-W, it was *S. epidermidis* > *E. coli* = *S. aureus* > *P. aeruginosa*. Regardless of the conditions, the selectivity was always high in the case of *S. epidermidis*.

## 3. Discussion

We conducted an extensive investigation on the cytotoxicity data of KSL, KSL-W, and Dadapin-1 on different relevant cell lines as functions of the presence/absence of serum. Our investigation was based on >98% purified peptides and, in our experimental work, we purposely utilized peptides from different sources. 

Although Dadapin-1 showed the lowest toxicity to eukaryotic cells, KSL exhibited the highest selectivity and outperformed the other two peptides. This finding has never been reported before and highlights the importance of studies that address the level of selectivity of new bioactive molecules. Unfortunately, although very relevant for qualifying a compound as a true antibacterial (not simply as toxic to both prokaryotic as well as eukaryotic cells), this information is generally missing for many antimicrobial peptides currently archived in dedicated AMP databases. 

At high concentrations, KSL-W revealed some cytotoxicity, which was more pronounced in the absence of serum, and its selectivity more resembled that of Dadapin-1 rather than that of KSL, despite the structural similarity of the two decapeptides. 

In scientific literature, He et al. (2020) [31] previously reported that KSL-W was not cytotoxic to MC3T3-E1 pre-osteoblasts up to a concentration of 1 mg/mL. Based on the MTT assay, KSL-W did not significantly alter cell metabolism throughout the entire range of concentrations. Other studies aimed at ascertaining the effects of KSL-W on gingival fibroblasts were conducted by Park et al. (2017) [32]. Over a period of 3 to 6 days, KSL-W concentrations in the range of 10–100 µg/mL were found to be non-cytotoxic, as well as promote fibroblast growth and cell migration, ultimately, stimulating wound healing. Furthermore, pro-proliferative activities were reported by Kosikowska et al. [33] when KSL-W was used at concentrations of 1–25 μg/mL to treat HaCaT human keratinocytes for 72 h.

The different results observed in our study could—at least in part—be explained by the fact that, after adding the treatment, the final concentration of FBS in the culture medium was 5%. It may well be that the double concentration of FBS (10%) used in the work of He et al. [31] could have reduced or abolished the cytotoxic effects. This explanation is even truer for the results reported by Park et al. (2017) [32]. Indeed, our results show the absence of cytotoxicity for a KSL-W concentration of up to 112.5 μg/mL, when the peptide was assayed on fibroblast-like L929 cells with half the serum concentration. Moreover, after just 24 h of cell culture, the mean metabolic activities of MG63 cells and L929 cells exposed to KSL-W concentrations lower than 100 μg/mL appeared slightly increased both in the presence and, more interestingly, absence of serum (Figure 3). Such a low increase in cell metabolism with respect to the reference control could suggest greater cell growth over longer time periods of cell culture. Interestingly, it was observed that, as a trend, this property is also shared by the other two peptides when below the threshold of cytotoxic concentrations. 

Regarding KSL cytotoxicity, Concannon et al. [22] documented the ‘good’ cytocompatibility of this peptide, which was not found to affect the cell viability of human gingival fibroblasts up to a concentration of 1 mg/mL. In the present study, KSL was not found to alter the metabolic activity of murine fibroblast-like L929 cells cultured in the presence of serum, even at the highest concentration tested of 450 μg/mL. In our investigation, the same concentration of peptide proved—to some extent—to be cytotoxic in the absence of serum. Thus, serum concentration appears to be a key factor when studying the effects of peptides on eukaryotic cells. The differences in the effects observed without serum are useful for interpreting the impact/interference of components such as serum proteases and anionic substances on the stability of AMPs, rather than to predict real in vivo toxicity. Indeed, serum components may be expected to be present in varying concentrations in physiologic fluids and the interstitial milieu.

Furthermore, as we have demonstrated clearly in this work, in addition to the culture conditions, cytotoxic effects are largely influenced by the cell line in use. Here, rapidly growing secondary cell lines, such as L929 and MG63 cells, were less affected by higher peptide concentrations than hMSCs primary cells. Fast cell growth could play a role in the susceptibility of cells. In addition, it should be noted that the serum itself is needed for sustained cell growth, and the total absence of FBS could also alter basal cell behavior. 

For the correct evaluation of the selectivity, both IC_50_ and MIC should probably be ascertained using the same culture medium that more closely simulates the in vivo environment, avoiding conditions in which the biologically active fraction of the peptide may differ. In this regard, many efforts have been made to identify an appropriate culture medium that includes blood components or artificially simulated physiologic fluids for testing minimal inhibitory concentrations in physiologically relevant conditions [34,35]. Nevertheless, the formulations of such ideal media are uniquely optimized for testing the microbiological properties of a putative antibacterial agent and cannot be applied ‘tout court’ for testing its cytocompatibility as well. Moreover, something that is often overlooked in the experimental schemes is that tissue cells themselves, with their electronegatively charged surfaces, can compete with, sequester, and reduce the bioactive fraction of free polycationic peptides in a solution. Future testing conditions should contemplate co-culture conditions in which MBC and IC_50_ are assessed in a single assay, where the measure of the S.I. could be more correctly estimated exactly under the same circumstances and with the same exposure to the bioactive agent.

The present study explored the cytocompatibility and selectivity of three antimicrobial peptides. Lack of selectivity is indeed a main limitation for most candidate antimicrobial peptides. Together, our past [27] and present observations contribute to defining the biological properties of KSL, KSL-W, and Dadapin-1 under different scenarios, including different cell lines and culture conditions. They suggest that these molecules possess interesting antibacterial and cytocompatibility properties and warrant further investigations into the potential roles they could play in the production of bioactive anti-infective materials in orthopedics. Certainly, these are not the only requirements that AMPs must fulfill to enter clinical use. While Dadapin-1 is a peptide whose discovery was rather recent, KSL-W has already entered initial clinical safety trials (combined phase 1/2a) to be used as an antiplaque agent [36]. The long regulatory pathways for the approval of clinical use of new bioactive biomaterials have, to date, favored the use of bioactive compounds already approved for local and systemic uses, such as conventional antibiotics or materials that have long been used in orthopedic surgery and are intrinsically antibacterial, such as silver. However, the spread of antimicrobial resistance among most bacterial clinical isolates, as well as the limited efficacy of current antibacterial solutions in preventing implant infections, underscore the need for new solutions to be found, and AMPs could certainly play a role. Other limitations that may be obstacles to the clinical use of AMPs are related to their high costs, low yield of production, and the ecological impact of large-scale production. Indeed, the currently widely used solid-phase peptide synthesis (SPPS) to chemically synthesize AMPs involves high production costs and low synthesis yields, making use of toxic solvents that may impact the environment. Statistical methodologies could help estimate optimal conditions for the production of AMPs. In this connection, response surface methodology using a Box–Behnken design turned out to be useful for planning large-scale production of AMPs from *L. plantarum* [37]. Nonetheless, advancements in greener technologies for scaling up production and abating upstream and downstream production costs are advancing and could offer new unprecedented perspectives on this front [38,39].

## 4. Materials and Methods

### 4.1. AMPs 

The synthetic peptides KSL (aminoacidic sequence: KKVVFKVKFK), KSL-W (aminoacidic sequence: KKVVFWVKFK), and Dadapin-1, consisting of 23 amino acids (aminoacidic sequence: GLLRASSKWGRKYYVDLAGCAKA), were purchased from GenScript (Shanghai, China) and ProteoGenix Sas (Schiltigheim, France), with a degree of purity >98%. Upon arrival, the peptides were stored at −80 °C for long-term storage. For the experimental work, the peptides were diluted to a concentration of 1 mg/mL in sterile deionized water (as recommended by the suppliers). Peptide aliquots were stored at −80 °C and, once thawed, were used entirely within a day.

### 4.2. Cytotoxicity Testing 

#### 4.2.1. Eucaryotic Cell Lines

The cytotoxicity of the test AMPs was assayed on three different mammalian cell lines, namely, the human osteosarcoma cell line MG63 (ATCC, Rockville, MD, USA); the murine immortalized fibroblast line L929 (ATCC, Rockville, MD, USA), and bone marrow-derived, human mesenchymal cells (hMSCs, BioWhittaker Inc., Walkersville, MD, USA). Experiments were performed both in the presence and absence of fetal bovine serum (FBS).

#### 4.2.2. Cell Culture

MG63 and L929 cells were thawed from frozen stocks and routinely cultured DMEM growth medium (GIBCO, Thermo Fisher Scientific, Waltham, MA, USA), supplemented with 10% fetal bovine serum (FBS, Sigma Aldrich, Milan, Italy), and penicillin/streptomycin (10,000 U/mL penicillin, 10 mg/mL streptomycin, Sigma-Aldrich, Milan, Italy), under standard culture conditions (i.e., at 37 °C, in a humidified atmosphere with 5% CO_2_). Every 2–3 days, cells were regularly sub-cultured using a Trypsin-EDTA solution (Sigma-Aldrich, Milan, Italy) or TrypLE Select 1X (GIBCO, Thermo Fisher Scientific, Waltham, MA, USA) to detach the adhered cells. Conversely, hMSCs were thawed from frozen stock and cultured in Alfa-MEM medium (Lonza, Euroclone S.p.A, Pero, MI, Italy) supplemented with 10% FBS and penicillin/streptomycin. The medium was replaced every 2–3 days. Cells were sub-cultured using stable Glutamine Stable 100× 200 mM (Lonza), once a week. 

#### 4.2.3. Bioluminescent ATP Assay for Cell Metabolic Activity

The effects of the AMPs were assayed via a bioluminescent ATP assay. A similar protocol was adopted for the different cell lines. For the experiments conducted using the medium supplemented with FBS, 96-well tissue-culture treated plates (96-Well, Nunclon Delta White, Life Technologies, Monza, Italy) were prepared by adding to each well 100 μL of a suspension of cells in complete medium at a concentration of 5 × 10^4^ cells/mL. After 1 day of incubation at 37 °C, under standard cell culture conditions, 96-well plates were treated with serial dilutions of the test AMPs by adding 100 µL of each treatment to 100 µL of medium in each well. The initial dilution was prepared by adding 1 volume of 10× Dulbecco’s Phosphate-Buffered Saline (DPBS, 14200-059, Life Technologies, Monza, Italy) to 9 volumes of the stock solution of each AMP in water (1 mg/mL), and further dilutions were made in 1× DPBS covering a range of 900–0.44 µg of peptides/mL. After the 1:1 dilution, the final test concentrations were in the range of 450–0.22 µg/mL. Triplicate wells were used for each dilution and the test was replicated in at least 3 independent experiments. The reference control was obtained by treating the cells with 1× DPBS. The cytotoxicity tests included the medium control, consisting of a medium fully supplemented with FBS and antibiotics, and a positive control solution, consisting of 0.5% Tween 20 (Sigma-Aldrich, Milan, Italy) in 1× DPBS. All these controls were tested in quadruplicate wells. To assay the cytotoxicity in the absence of FBS, after the first day of incubation of the plate, the medium was removed, and the wells were washed once with 100 µL of DPBS prior to replacing the old medium with 100 µL of a fresh FBS-free medium and adding 100 µL of each treatment to the wells.

Thereafter, the cell cultures were treated for 24 h at 37 °C. At the end of the incubation period, the 96-well plates were processed as follows: a volume of 100 μL of the medium was removed from each well and replaced with 100 μL of the solution of the Cell-Titer Glo 2.0 Assay (Promega, Milano, Italy). After 10 min in the dark, the luminescence of the microplates was read by the Modulus II Multifunction Plate Reader (Turner BioSystems, Sunnyvale, CA, USA). The blank was subtracted from all readings, and the luminescence values were normalized, with the mean of the reference control set to 100%. The criteria for the validation of the plates were as follows: Regardless of the cell line used, the mean percent metabolic activity of the positive control was expected to be lower than 5%. For both secondary cell lines, MG63 and L929, the medium control was expected to produce a mean percent metabolic activity in the range of 90–120% (105% ± 15%) in the presence of FBS and 80–110% (95% ± 15%) in the absence of the serum. For hMSCs, in view of the greater variability of the behaviors of primary cell lines, the medium control was expected to produce a mean percent metabolic activity in the range of 85–125% (105% ± 20%) in the presence of FBS and 80–110% (95% ± 15%) in the absence of serum. 

#### 4.2.4. Cytomorphology Evaluation of MG63 Cells

To assess the effects of the peptides on the cell morphology, MG63 cells were cultured on glass coverslips. Microscope round cover glasses (Ø 15 mm, Superior Marienfeld, Lauda-Königshofen, Germany) were sterilized by autoclaving and one coverslip was added to each well of a 12-well plate (CL301, Clearline, Biosigma S.p.A., Cona, Italy). MG63 cells were cultured as described above and seeded at a concentration of 4.5 × 10^4^ cells/well, with a volume of 1 mL in each well. Then, the cells were cultured under standard culture conditions at 37 °C. After 24 h, the medium was removed and replaced with 500 µL of fresh medium. Treatments were performed in duplicate wells by adding 500 µL of 1× DPBS (reference control); 500 µL of fresh medium (negative control); Dadapin-1 at a concentration of 450 µg/mL (the highest concentration tested); KSL at a concentration of 225 µg/mL (the first concentration causing a statistically significant decrease in cell metabolism); KSL-W at a concentration of 112.5 µg/mL (the first concentration causing a statistically significant decrease in cell metabolism), and a non-cytotoxic concentration of 56.3 µg/mL. The plates were further incubated for 24 h at 37 °C, after which, the cells seeded onto glass coverslips were fixed for 10 min in 2% formaldehyde (CAS. N. 30525-89-4 Darmstadt, Germany) at RT. After three washes in DPBS, cells were permeabilized for 10 min in PBT (D-PBS 1× + 0.1% Triton X100), then incubated in a blocking solution (1x DPBS + 5% BSA) for 30 min. After three washes, cells were incubated with 4 µg/mL of Phalloidin-FITC (Sigma-Aldrich, Darmstadt, Germany; P5282) in 1× D-PBS for 45 min at RT. The nuclei were stained with 5 µg/mL of Hoechst 33342 (Life Technologies, Monza, Italy) in 1× D-PBS for 10 min. After washing in DPBS, coverslips were mounted in ProLong Diamond Antifade Mountant (Life Technologies, Monza, Italy, P36961), and imaged at 400× magnification with a Nikon Eclipse TE2000-U inverted epifluorescence microscope equipped with a calibrated color camera (Nikon DS-Vi1-U3 CCD).

### 4.3. Statistics

The cytotoxicity data from the ATP bioluminescence assay were used both to determine the first concentrations of each peptide causing a reduction in cell activity and to estimate the inhibitory concentration (IC_50_) that resulted in a 50% reduction in metabolic activity. In the first case, the results are presented as the mean ± standard deviations of at least three experiments performed in triplicate. The Shapiro–Wilk test was performed using GraphPad software (ver. 10.4) to evaluate the normal distribution of the quantitative variables (information is provided in Table S1). The results of the treated cell cultures normalized with the reference control set to 100% were analyzed using one-way ANOVA followed by the Bonferroni/Dunn test (significance level: 5%) using GraphPad software in cases where there was a normal distribution and homogeneity of variances. When the contrary was found, the Kruskal–Wallis test was performed using GraphPad software, with each treatment compared to the reference control. 

Conversely, to assess the IC_50_ values of the three peptides, the Quest Graph™ IC50 Calculator, AAT Bioquest, Inc. (Pleasanton, CA, USA) (https://www.aatbio.com/tools/ic50-calculator (accessed on 25 November 2024)) online resource was used, as described earlier in [28]. The IC_50_ value was extrapolated for each single experiment. Thereafter, for each peptide and testing condition (with and w/o serum), the mean IC_50_ value and the interval of confidence were calculated by StatView (version 5.0.1, Sas Institute Inc., Cary, NC, USA). 

IC_50_ values obtained for the peptides were then used to extrapolate the selectivity index (S.I.). For this purpose, the minimal inhibitory concentrations (MICs) of the three peptides obtained for four relevant clinical orthopedic isolates were taken from another recent study [27], in which the same peptides were characterized for their antimicrobial properties. MICs were tested via the dilution method in Mueller Hinton Broth II (MHB II), assessing antibacterial activity in both undiluted (100% MHB II) and diluted (20% MHB II) media. The selectivity was extrapolated from the formula S.I. = IC_50_/MIC. 

## 5. Conclusions

Overall, our findings provide a thorough and detailed characterization of the cytocompatibility and in vitro performances of KSL, KSL-W, and Dadapin-1. KSL-W and KSL emerge as peptides with great potential for the production of anti-infective coatings—the former for its stable activity, which is less influenced by the interference of polyanionic substances, the latter for its high selectivity, and both for their appreciable cytocompatibility profiles.

Dadapin-1 shows selectivity just slightly lower than that expressed by KSL-W, as well as minimal toxicity. 

The data that emerged on the varying sensitivities of different cell types to AMPs highlight the importance of extending cytotoxicological studies of AMPs to a broad panel of cell lines, including cell phenotypes that appear more vulnerable such as hMSCs. The emerging trend of pro-proliferative and metabolism-enhancing effects by AMPs at sub-cytotoxic concentrations on eukaryotic cells suggests that they could be common traits across different peptides. This has some interesting implications for the production of multifunctional biomaterials. All these aspects deserve to be explored in greater depth along the path toward applications in the orthopedic field.

## Figures and Tables

**Table 1:** MIC values in µg/mL used to calculate the I.S. (from [27]).

Bacterial Strain	MICs in 100% MHB II			MICs in 20% MHB II		
	KSL	KSL-W	Dadapin-1	KSL	KSL-W	Dadapin-1
*S. aureus* ATCC25923	31.25	15.63	-	1.95	3.91	15.63
*S. epidermidis cra*4034	3.91	3.91	500	0.98	1.95	7.81
*E. coli cra*4038	7.81	3.91	-	1.95	3.91	31.25
*P. aeruginosa cra*3840	125	31.25	-	3.91	7.81	31.25

Legend: undiluted MHB II (nominal value: 100%; final concentration in the assay: 50%); diluted MHB II (nominal value: 20%; final concentration in the assay: 10%).

**Table 2:** S.I. values under different test conditions.

AMPs	IC_50_ *	MICs in 100% MHB II				MICs in 20% MHB II			
		SA	SE	EC	PA	SA	SE	EC	PA
S.I. values based on IC_50_ values of hMSCs cultured without FBS									
Dadapin-1	256.3 (176.7–336.0)	-	0.5 (0.4–0.7)	-	-	16.4 (11.3–21.5)	32.8 (22.6–43.0)	8.2 (5.7–10.8)	8.2 (5.7–10.8)
KSL	133.7 (120.1–147.2)	4.3 (3.8–4.7)	34.2 (30.7–37.6)	17.1 (15.4–18.8)	1.1 (1.0–1.2)	68.6 (61.6–75.5)	136.4 (122.6–150.2)	68.6 (61.6–75.5)	34.2 (30.7–37.6)
KSL-W	73.1 (60.2–86.0)	4.7 (3.9–5.5)	18.7 (15.4–22.0)	18.7 (15.4–22.0)	2.3 (1.9–2.8)	18.7 (15.4–22.0)	37.5 (30.9–44.1)	18.7 (15.4–22.0)	9.4 (7.7–11.0)
S.I. values based on IC_50_ values of hMSCs cultured with FBS									
Dadapin-1	404.7 (342.5–466.9)	-	0.8 (0.7–0.9)	-	-	25.9 (21.9–29.9)	51.8 (43.9–59.8)	13.0 (11.0–14.9)	13.0 (11.0–14.9)
KSL	207.4 (195.0–219.8)	6.6 (6.2–7.0)	53.0 (49.9–56.2)	26.6 (25.0–28.1)	1.7 (1.6–1.8)	106.4 (100.0–112.7)	211.6 (199.0 -224.3)	106.4 (100.0–112.7)	53.0 (49.9–56.2)
KSL-W	110.5 (99.2–121.7)	7.1 (6.3–7.8)	28.3 (25.4–31.1)	28.3 (25.4–31.1)	3.5 (3.2–3.9)	28.3 (25.4–31.1)	56.7 (50.9–62.4)	28.3 (25.4–31.1)	14.1 (12.7–15.6)

Legend: * IC_50_ for the corresponding AMP tested on hMSCs; in brackets is reported the confidence interval (C.I.) for IC_50_ and S.I.; SA, *S. aureus* ATCC25923; SE, *S. epidermidis cra*4034; EC, *E. coli cra*4038; PA, *P. aeruginosa cra*3840.

## Data Availability

The experimental data are presented in detail as supplementary materials together with all statistical analyses. Further insights into the data presented in this study are available upon request from the corresponding authors.

## Supplementary Materials

The following supporting information can be downloaded at https://www.mdpi.com/article/10.3390/ijms252413241/s1.
