# Supplementary material for: Assessing Cytotoxicity, Proteolytic Stability, and Selectivity of Antimicrobial Peptides: Implications for Orthopedic Applications
Source: Int J Mol Sci. 2024 Dec 10;25(24):13241. doi: 10.3390/ijms252413241 (PMC11678430; doi:10.3390/ijms252413241)
Supplement: Supplementary file 1 [file ijms-25-13241-s001.zip › ijms-3304986-supplementary.pdf]

Article

Assessing Cytotoxicity, Proteolytic Stability, and Selectivity of Antimicrobial Peptides: Implications for Orthopedic Applications

Davide Campoccia [1,*], Giulia Bottau [1,†], Andrea De Donno [1,†], Gloria Bua [1], Stefano Ravaioli [1], Eleonora Capponi [1], Giovanna Sotgiu [2], Chiara Bellotti [3], Silvia Costantini [4,5,‡] and Carla Renata Arciola [4,5,*]

[1] Laboratorio di Patologia delle Infezioni Associate all'Impianto, IRCCS Istituto Ortopedico Rizzoli, Via di Barbiano 1/10, 40136 Bologna, Italy; giulia.bottau@ior.it (G.B.); andrea.dedonno@ior.it (A.D.D.); gloria.bua@ior.it (G.B.); stefano.ravaioli@ior.it (S.R.); eleonora.capponi@ior.it (E.C.)
[2] Institute for Organic Synthesis and Photoreactivity (ISOF), National Research Council, Via Gobetti 101, 40129 Bologna, Italy; giovanna.sotgiu@isof.cnr.it
[3] Osteoncology, Bone and Soft Tissue Sarcomas and Innovative Therapies Unit, IRCCS Istituto Ortopedico Rizzoli, Via di Barbiano 1/10, 40136 Bologna, Italy; chiara.bellotti@ior.it
[4] Department of Medical and Surgical Sciences (DIMEC), University of Bologna, Via San Giacomo 14, 40126 Bologna, Italy; silvia.costantini4@studio.unibo.it
[5] Laboratory of Immunorheumatology and Tissue Regeneration, Laboratory of Pathology of Implant Infections, IRCCS Istituto Ortopedico Rizzoli, Via di Barbiano 1/10, 40136 Bologna, Italy
* Correspondence: davide.campoccia@ior.it (D.C.); carlarenata.arciola@ior.it (C.R.A.)
† These authors contributed equally to this work.
‡ This author is a medical resident of the Postgraduate Medical School in Clinical Pathology and Clinical Biochemistry of the University of Bologna.

Citation: To be added by editorial staff during production.

Academic Editor: Firstname Lastname

Received: date
Accepted: date
Published: date

Publisher's Note: MDPI stays neutral with regard to jurisdictional claims in published maps and institutional affiliations.

Copyright: © 2022 by the authors. Submitted for possible open access publication under the terms and conditions of the Creative Commons Attribution (CC BY) license (https://creativecommons.org/licenses/by/4.0/).

Table S1. Analysis of normal distribution of the cytotoxicity data by Shapiro-Wilk test

Shapiro-Wilk test	Reference Control	Medium Control	Positive Control	450 µg/mL	225 µg/mL	112.5 µg/mL
**Dadapin-1 tested on L929 Cells with FBS**						
W	0.9408	0.9686	0.9119	0.9364	0.9832	0.9351
P value	0.5081	0.8956	0.2258	0.5444	0.9787	0.5309
Passed normality test (alpha=0.05)?	Yes	Yes	Yes	Yes	Yes	Yes
P value summary	ns	ns	ns	ns	ns	ns
**Dadapin-1 tested on L929 Cells without FBS**						
W	0.9373	0.9119	0.969	0.7898	0.9096	0.9256
P value	0.3171	0.1247	0.8216	0.0072	0.2111	0.3355
Passed normality test (alpha=0.05)?	Yes	Yes	Yes	**No**	Yes	Yes
P value summary	ns	ns	ns	**	ns	ns
**Dadapin-1 tested on hMSCs Cells with FBS**						
W	0.9576	0.8932	0.9305	0.9632	0.9538	0.9819
P value	0.7493	0.1296	0.385	0.8311	0.7313	0.9732
Passed normality test (alpha=0.05)?	Yes	Yes	Yes	Yes	Yes	Yes
P value summary	ns	ns	ns	ns	ns	ns
**Dadapin-1 tested on hMSCs Cells without FBS**						
W	0.9576	0.9675	0.8541	0.8579	0.9615	0.9395
P value	0.7491	0.8825	0.0413	0.0909	0.8142	0.5771
Passed normality test (alpha=0.05)?	Yes	Yes	**No**	Yes	Yes	Yes

P value summary	ns	ns	*	ns	ns	ns
**KSL tested on MG63 Cells with FBS**						
W	0.9473	0.9536	0.8473	0.909	0.9653	0.8971
P value	0.5979	0.6898	0.034	0.3087	0.8514	0.2357
Passed normality test (alpha=0.05)?	Yes	Yes	**No**	Yes	Yes	Yes
P value summary	ns	ns	*	ns	ns	ns
**KSL tested on MG63 Cells without FBS**						
W	0.9811	0.951	0.9364	0.8385	0.9306	0.9806
P value	0.9718	0.505	0.3077	0.0265	0.3867	0.9859
Passed normality test (alpha=0.05)?	Yes	Yes	Yes	**No**	Yes	Yes
P value summary	ns	ns	ns	*	ns	ns
**KSL tested on L929 Cells with FBS**						
W	0.9364	0.9422	0.8543	0.9231	0.9739	0.9512
P value	0.4531	0.5272	0.0415	0.4186	0.9257	0.7033
Passed normality test (alpha=0.05)?	Yes	Yes	**No**	Yes	Yes	Yes
P value summary	ns	ns	*	ns	ns	ns
**KSL tested on L929 Cells without FBS**						
W	0.9616	0.8936	0.8955	0.9609	0.8534	0.9397
P value	0.8061	0.1313	0.1389	0.8079	0.0811	0.5785
Passed normality test (alpha=0.05)?	Yes	Yes	Yes	Yes	Yes	Yes
P value summary	ns	ns	ns	ns	ns	ns

	**Reference Control**	**Medium Control**	**Positive Control**	**225 µg/mL**	**112.5 µg/mL**	**56.3 µg/mL**
**KSL tested on hMSCs Cells with FBS**						

W	0.8775	0.7535	0.8649	0.9433	0.9655	0.9481
P value	0.0814	0.0029	0.0563	0.6167	0.854	0.6693
Passed normality test (alpha=0.05)?	Yes	**No**	Yes	Yes	Yes	Yes
P value summary	ns	**	ns	ns	ns	ns
**KSL tested on hMSCs Cells without FBS**						
W	0.9189	0.9085	0.9634	0.9372	0.9204	0.8495
P value	0.1622	0.1102	0.7237	0.4626	0.2896	0.0362
Passed normality test (alpha=0.05)?	Yes	Yes	Yes	Yes	Yes	**No**
P value summary	ns	ns	ns	ns	ns	*
**KSL-W tested on MG63 Cells with FBS**						
W	0.9473	0.9536	0.8473	0.5089	0.8528	0.9913
P value	0.5979	0.6898	0.034	<0.0001	0.0799	0.9976
Passed normality test (alpha=0.05)?	Yes	Yes	**No**	**No**	Yes	Yes
P value summary	ns	ns	*	****	ns	ns
**KSL-W tested on MG63 Cells without FBS**						
W	0.9811	0.951	0.9364	0.91	0.9455	0.9257
P value	0.9718	0.505	0.3077	0.2133	0.573	0.3364
Passed normality test (alpha=0.05)?	Yes	Yes	Yes	Yes	Yes	Yes
P value summary	ns	ns	ns	ns	ns	ns
**KSL-W tested on L929 Cells with FBS**						
W	0.9364	0.9422	0.8519	0.8708	0.9477	0.8829
P value	0.4529	0.5272	0.0387	0.1255	0.6648	0.1686
Passed normality test (alpha=0.05)?	Yes	Yes	**No**	Yes	Yes	Yes
P value summary	ns	ns	*	ns	ns	ns
**KSL-W tested on L929 Cells without FBS**						
W	0.9616	0.8937	0.8992	0.9335	0.8915	0.84
P value	0.8061	0.1316	0.1547	0.5149	0.2065	0.0578
Passed normality test (alpha=0.05)?	Yes	Yes	Yes	Yes	Yes	Yes

	Reference Control	Medium Control	Positive Control	112.5 µg/mL	56.3 µg/mL	28.1 µg/mL
P value summary	ns	ns	ns	ns	ns	ns
**KSL-W tested on hMSCs Cells with FBS**						
W	0.8775	0.7535	0.8719	0.908	0.8713	0.8412
P value	0.0813	0.0029	0.069	0.3021	0.127	0.0596
Passed normality test (alpha=0.05)?	Yes	**No**	Yes	Yes	Yes	Yes
P value summary	ns	**	ns	ns	ns	ns
**KSL-W tested on hMSCs Cells without FBS**						
W	0.9189	0.9085	0.9634	0.9162	0.9089	0.8739
P value	0.1622	0.1102	0.7237	0.256	0.2063	0.0732
Passed normality test (alpha=0.05)?	Yes	Yes	Yes	Yes	Yes	Yes
P value summary	ns	ns	ns	ns	ns	ns

Table S2. Choice of parametric or non-parametric statistical analysis driven by the Shapiro-Wilk Test outcomes

Data set		ANOVA	KRUSKAL-WALLIS
Dadapin-1	L929 with FBS	X	
	L929 w/o FBS		X
	hMSC with FBS	X	
	hMSC w/o FBS		X
KSL	MG63 with FBS		X
	MG63 w/o FBS		X
	L929 with FBS		X
	L929 w/o FBS	X	
	hMSC with FBS		X
	hMSC w/o FBS		X
KSL-W	MG63 with FBS		X
	MG63 w/o FBS	X	
	L929 with FBS		X
	L929 w/o FBS	X	
	hMSC with FBS		X
	hMSC w/o FBS	X	

Table S3. Parametric and non-parametric statistical analysis of cytotoxicity data

Dadapin-1 tested on L929 Cells with FBS

Bonferroni's multiple comparisons test	Mean Diff.	95.00% CI of diff.	Below threshold?	Summary	Adjusted P Value	
Reference Control vs. Medium Control	-7.816	-15.59 to -0.04365	Yes	*	0.0476	A-B
Reference Control vs. Positive Control	99.95	92.18 to 107.7	Yes	****	<0.0001	A-C
Reference Control vs. 450	18.7	10.31 to 27.09	Yes	****	<0.0001	A-D
Reference Control vs. 225	-7.458	-15.85 to 0.9364	No	ns	0.1288	A-E
Reference Control vs. 112,5	-9.949	-18.34 to -1.555	Yes	**	0.0091	A-F

Dadapin-1 tested on L929 Cells without FBS

Dunn's multiple comparisons test	Mean rank diff.	Significant?	Summary	Adjusted P Value	A-?	
Reference Control vs. Medium Control	1.313	No	ns	>0.9999	B	Medium Control
Reference Control vs. Positive Control	32.31	Yes	***	0.0009	C	Positive Control
Reference Control vs. 450	-10.85	No	ns	>0.9999	D	450
Reference Control vs. 225	-22.94	No	ns	0.069	E	225
Reference Control vs. 112,5	-22.85	No	ns	0.0707	F	112.5

Test details	Mean rank 1	Mean rank 2	Mean rank diff.	n1	n2	Z
Reference Control vs. Medium Control	40.81	39.5	1.313	16	16	0.1522
Reference Control vs. Positive Control	40.81	8.5	32.31	16	16	3.747
Reference Control vs. 450	40.81	51.67	-10.85	16	12	1.165
Reference Control vs. 225	40.81	63.75	-22.94	16	12	2.462
Reference Control vs. 112,5	40.81	63.67	-22.85	16	12	2.453

Dadapin-1 tested on hMSCs Cells with FBS

Bonferroni's multiple comparisons test	Mean Diff.	95.00% CI of diff.	Below threshold?	Summary	Adjusted P Value	
Reference Control vs. Medium Control	-9.345	-18.94 to 0.2548	No	ns	0.063	A-B
Reference Control vs. Positive Control	99.95	90.35 to 109.5	Yes	****	<0.0001	A-C
Reference Control vs. 450	57.85	47.49 to 68.22	Yes	****	<0.0001	A-D
Reference Control vs. 225	8.542	-1.826 to 18.91	No	ns	0.216	A-E
Reference Control vs. 112,5	-7.52	-17.89 to 2.848	No	ns	0.4538	A-F

Dadapin-1 tested on hMSCs Cells without FBS

Dunn's multiple comparisons test	Mean rank diff.	Significant?	Summary	Adjusted P Value	A-?	
Reference Control vs. Medium Control	-3.5	No	ns	>0.9999	B	Medium Control
Reference Control vs. Positive Control	35.75	Yes	****	<0.0001	C	Positive Control
Reference Control vs. 450	25.25	Yes	**	0.0089	D	450
Reference Control vs. 225	16.25	No	ns	0.2219	E	225
Reference Control vs. 112,5	-12.75	No	ns	0.5735	F	112.5

Test details	Mean rank 1	Mean rank 2	Mean rank diff.	n1	n2	Z
Reference Control vs. Medium Control	42.25	45.75	-3.5	12	12	0.4677
Reference Control vs. Positive Control	42.25	6.5	35.75	12	12	4.777
Reference Control vs. 450	42.25	17	25.25	12	9	3.124
Reference Control vs. 225	42.25	26	16.25	12	9	2.01
Reference Control vs. 112,5	42.25	55	-12.75	12	9	1.577

KSL tested on MG63 Cells with FBS

Dunn's multiple comparisons test	Mean rank diff.	Significant?	Summary	Adjusted P Value	A-?

Reference Control vs. Medium Control	-6.5	No	ns	>0.9999	B	Medium Control
Reference Control vs. Positive Control	34.75	Yes	****	<0.0001	C	Positive Control
Reference Control vs. 450,00	24.25	Yes	*	0.0135	D	450
Reference Control vs. 225,00	13.03	No	ns	0.5351	E	225
Reference Control vs. 112,50	-10.19	No	ns	>0.9999	F	112.5
Test details	Mean rank 1	Mean rank 2	Mean rank diff.	n1	n2	Z
Reference Control vs. Medium Control	41.25	47.75	-6.5	12	12	0.8686
Reference Control vs. Positive Control	41.25	6.5	34.75	12	12	4.644
Reference Control vs. 450,00	41.25	17	24.25	12	9	3
Reference Control vs. 225,00	41.25	28.22	13.03	12	9	1.612
Reference Control vs. 112,50	41.25	51.44	-10.19	12	9	1.261

KSL tested on MG63 Cells without FBS

Dunn's multiple comparisons test	Mean rank diff.	Significant?	Summary	Adjusted P Value	A-?	
Reference Control vs. Medium Control	0.8125	No	ns	>0.9999	B	Medium Control
Reference Control vs. Positive Control	46.5	Yes	****	<0.0001	C	Positive Control
Reference Control vs. KSL 450,00	32.5	Yes	**	0.0024	D	KSL 450,00
Reference Control vs. KSL 225,00	10.67	No	ns	>0.9999	E	KSL 225,00
Reference Control vs. KSL 112,50	-18.75	No	ns	0.2206	F	KSL 112,50
Test details	Mean rank 1	Mean rank 2	Mean rank diff.	n1	n2	Z
Reference Control vs. Medium Control	55	54.19	0.8125	16	16	0.09421
Reference Control vs. Positive Control	55	8.5	46.5	16	16	5.392
Reference Control vs. KSL 450,00	55	22.5	32.5	16	12	3.489
Reference Control vs. KSL 225,00	55	44.33	10.67	16	12	1.145
Reference Control vs. KSL 112,50	55	73.75	-18.75	16	12	2.013

KSL tested on L929 Cells with FBS

Dunn's multiple comparisons test	Mean rank diff.	Significant?	Summary	Adjusted P Value	A-?	
Reference Control vs. Medium Control	-9.333	No	ns	>0.9999	B	Medium Control
Reference Control vs. Positive Control	20.75	Yes	*	0.0278	C	Positive Control
Reference Control vs. KSL 450,00	-4.528	No	ns	>0.9999	D	KSL 450,00
Reference Control vs. KSL 225,00	-22.53	Yes	*	0.0266	E	KSL 225,00
Reference Control vs. KSL 112,50	-21.42	Yes	*	0.0403	F	KSL 112,50

Test details	Mean rank 1	Mean rank 2	Mean rank diff.	n1	n2	Z
Reference Control vs. Medium Control	27.25	36.58	-9.333	12	12	1.247
Reference Control vs. Positive Control	27.25	6.5	20.75	12	12	2.773
Reference Control vs. KSL 450,00	27.25	31.78	-4.528	12	9	0.5602
Reference Control vs. KSL 225,00	27.25	49.78	-22.53	12	9	2.787
Reference Control vs. KSL 112,50	27.25	48.67	-21.42	12	9	2.65

KSL tested on L929 Cells without FBS

Bonferroni's multiple comparisons test	Mean Diff.	95.00% CI of diff.	Below threshold?	Summary	Adjusted P Value	
Reference Control vs. Medium Control	11.25	-3.680 to 26.17	No	ns	0.3692	A-B
Reference Control vs. Positive Control	99.85	84.93 to 114.8	Yes	****	<0.0001	A-C
Reference Control vs. KSL 450,00	72.01	55.88 to 88.13	Yes	****	<0.0001	A-D
Reference Control vs. KSL 225,00	-7.681	-23.80 to 8.441	No	ns	>0.9999	A-E
Reference Control vs. KSL 112,50	-16.6	-32.73 to -0.4820	Yes	*	0.0384	A-F

KSL tested on hMSCs Cells with FBS

Dunn's multiple comparisons test	Mean rank diff.	Significant?	Summary	Adjusted P Value	A-?	
Reference Control vs. Medium Control	-18.67	No	ns	0.0631	B	Medium Control
Reference Control vs. Positive Control	26.92	Yes	**	0.0016	C	Positive Control

Reference Control vs. KSL 225	16.42	No	ns	0.2112	D	KSL 225
Reference Control vs. KSL 112,5	-10.81	No	ns	0.9063	E	KSL 112,5
Reference Control vs. KSL 56,3	-6.694	No	ns	>0.9999	F	KSL 56,3
Test details	Mean rank 1	Mean rank 2	Mean rank diff.	n1	n2	Z
Reference Control vs. Medium Control	33.42	52.08	-18.67	12	12	2.494
Reference Control vs. Positive Control	33.42	6.5	26.92	12	12	3.597
Reference Control vs. KSL 225	33.42	17	16.42	12	9	2.031
Reference Control vs. KSL 112,5	33.42	44.22	-10.81	12	9	1.337
Reference Control vs. KSL 56,3	33.42	40.11	-6.694	12	9	0.8282

KSL tested on hMSCs Cells without FBS

Dunn's multiple comparisons test	Mean rank diff.	Significant?	Summary	Adjusted P Value	A-?	
Reference Control vs. Medium Control	4.438	No	ns	>0.9999	B	Medium Control
Reference Control vs. Positive Control	48.19	Yes	****	<0.0001	C	Positive Control
Reference Control vs. KSL 225,00	34.19	Yes	**	0.0012	D	KSL 225,00
Reference Control vs. KSL 112.5	6.604	No	ns	>0.9999	E	KSL 112.5
Reference Control vs. KSL 56,3	-11.65	No	ns	>0.9999	F	KSL 56,3
Test details	Mean rank 1	Mean rank 2	Mean rank diff.	n1	n2	Z
Reference Control vs. Medium Control	56.69	52.25	4.438	16	16	0.5145
Reference Control vs. Positive Control	56.69	8.5	48.19	16	16	5.588
Reference Control vs. KSL 225,00	56.69	22.5	34.19	16	12	3.67
Reference Control vs. KSL 112.5	56.69	50.08	6.604	16	12	0.709
Reference Control vs. KSL 56,3	56.69	68.33	-11.65	16	12	1.25

KSL-W tested on MG63 Cells with FBS

Dunn's multiple comparisons test	Mean rank diff.	Significant?	Summary	Adjusted P Value	A-?	

Reference Control vs. Medium Control	-6.583	No	ns	>0.9999	B	Medium Control
Reference Control vs. Positive Control	35.58	Yes	****	<0.0001	C	Positive Control
Reference Control vs. KSL-W 225	25.28	Yes	**	0.0088	D	KSL-W 225
Reference Control vs. KSL-W 112,5	13.39	No	ns	0.4882	E	KSL-W 112,5
Reference Control vs. KSL-W 56,3	-6.167	No	ns	>0.9999	F	KSL-W 56,3
Test details	Mean rank 1	Mean rank 2	Mean rank diff.	n1	n2	Z
Reference Control vs. Medium Control	42.17	48.75	-6.583	12	12	0.8797
Reference Control vs. Positive Control	42.17	6.583	35.58	12	12	4.755
Reference Control vs. KSL-W 225	42.17	16.89	25.28	12	9	3.127
Reference Control vs. KSL-W 112,5	42.17	28.78	13.39	12	9	1.656
Reference Control vs. KSL-W 56,3	42.17	48.33	-6.167	12	9	0.7629

KSL-W tested on MG63 Cells without FBS

Bonferroni's multiple comparisons test	Mean Diff.	95.00% CI of diff.	Below threshold?	Summary	Adjusted P Value	
Reference Control vs. Medium Control	0.2971	-7.156 to 7.750	No	ns	>0.9999	A-B
Reference Control vs. Positive Control	99.78	92.33 to 107.2	Yes	****	<0.0001	A-C
Reference Control vs. KSL-W 225	99.72	91.67 to 107.8	Yes	****	<0.0001	A-D
Reference Control vs. KSL-W 112,5	49.02	40.97 to 57.07	Yes	****	<0.0001	A-E
Reference Control vs. KSL-W 56,3	-13.47	-21.52 to -5.416	Yes	****	<0.0001	A-F

KSL-W tested on L929 Cells with FBS

Dunn's multiple comparisons test	Mean rank diff.	Significant?	Summary	Adjusted P Value	A-?	
Reference Control vs. Medium Control	-9.917	No	ns	0.9256	B	Medium Control
Reference Control vs. Positive Control	30.75	Yes	***	0.0002	C	Positive Control
Reference Control vs. KSL-W 225	20.83	Yes	*	0.0498	D	KSL-W 225
Reference Control vs. KSL-W 112,5	6.167	No	ns	>0.9999	E	KSL-W 112,5

Reference Control vs. KSL-W 56,3	-16.28	No	ns	0.2201	F	KSL-W 56,3
Test details	Mean rank 1	Mean rank 2	Mean rank diff.	n1	n2	Z
Reference Control vs. Medium Control	37.5	47.42	-9.917	12	12	1.325
Reference Control vs. Positive Control	37.5	6.75	30.75	12	12	4.109
Reference Control vs. KSL-W 225	37.5	16.67	20.83	12	9	2.577
Reference Control vs. KSL-W 112,5	37.5	31.33	6.167	12	9	0.7629
Reference Control vs. KSL-W 56,3	37.5	53.78	-16.28	12	9	2.014

KSL-W tested on L929 Cells without FBS

Bonferroni's multiple comparisons test	Mean Diff.	95.00% CI of diff.	Below threshold?	Summary	Adjusted P Value	
Reference Control vs. Medium Control	11.25	1.102 to 21.39	Yes	*	0.0187	A-B
Reference Control vs. Positive Control	99.85	89.71 to 110.0	Yes	****	<0.0001	A-C
Reference Control vs. KSL-W 225	99.57	88.61 to 110.5	Yes	****	<0.0001	A-D
Reference Control vs. KSL-W 112,5	81.92	70.96 to 92.88	Yes	****	<0.0001	A-E
Reference Control vs. KSL-W 56,3	-12.89	-23.85 to -1.930	Yes	**	0.0099	A-F

KSL-W tested on hMSCs Cells with FBS

Dunn's multiple comparisons test	Mean rank diff.	Significant?	Summary	Adjusted P Value	A-?	
Reference Control vs. Medium Control	-20.67	Yes	*	0.0288	B	Medium Control
Reference Control vs. Positive Control	24.08	Yes	**	0.0064	C	Positive Control
Reference Control vs. KSL-W 112,5	13.58	No	ns	0.4643	D	KSL-W 112,5
Reference Control vs. KSL-W 56,3	-11.86	No	ns	0.7113	E	KSL-W 56,3
Reference Control vs. KSL-W 28,1	-16.19	No	ns	0.2256	F	KSL-W 28,1
Test details	Mean rank 1	Mean rank 2	Mean rank diff.	n1	n2	Z
Reference Control vs. Medium Control	30.58	51.25	-20.67	12	12	2.762

Reference Control vs. Positive Control	30.58	6.5	24.08	12	12	3.218
Reference Control vs. KSL-W 112,5	30.58	17	13.58	12	9	1.681
Reference Control vs. KSL-W 56,3	30.58	42.44	-11.86	12	9	1.467
Reference Control vs. KSL-W 28,1	30.58	46.78	-16.19	12	9	2.004

KSL-W tested on hMSCs Cells without FBS

Bonferroni's multiple comparisons test	Mean Diff.	95.00% CI of diff.	Below threshold?	Summary	Adjusted P Value	
Reference Control vs. Medium Control	3.909	-10.22 to 18.03	No	ns	>0.9999	A-B
Reference Control vs. Positive Control	99.95	85.82 to 114.1	Yes	****	<0.0001	A-C
Reference Control vs. KSL-W 112,5	88.97	73.72 to 104.2	Yes	****	<0.0001	A-D
Reference Control vs. KSL-W 56,3	15.03	-0.2282 to 30.28	No	ns	0.0571	A-E
Reference Control vs. KSL-W 28,1	-7.701	-22.96 to 7.555	No	ns	>0.9999	A-F

Table S4. IC_{50} – One sample analysis – T-TEST – Confidence of interval

One Sample Analysis
Hypothesized Mean = 0

	Mean	DF	t-Value	P-Value	95% Lower	95% Upper
MG63 KSL with FBS	282,955	2	33,629	,0009	246,753	319,157
MG63 KSL w/o FBS	291,429	3	10,998	,0016	207,102	375,756
MG63 KSL-W with FBS	128,230	2	39,809	,0006	114,370	142,089
MG63 KSL-W w/o FBS	111,074	3	108,449	<,0001	107,814	114,333
L929 KSL w/o FBS	354,065	2	21,050	,0022	281,694	426,436
L929 KSL-W with FBS	136,053	2	48,919	,0004	124,087	148,020
L929 KSL-W w/o FBS	87,011	2	26,430	,0014	72,846	101,176
hMSCs Dadapin-1 with FBS	404,694	2	27,989	,0013	342,482	466,906
hMSCs Dadapin-1 w/o FBS	256,347	2	13,850	,0052	176,711	335,983
hMSCs KSL with FBS	207,439	2	71,974	,0002	195,038	219,840
hMSCs KSL w/o FBS	133,665	3	31,341	<,0001	120,092	147,238
hMSCs KSL-W with FBS	110,454	2	42,337	,0006	99,229	121,679
hMSCs KSL-W w/o FBS	73,081	3	17,990	,0004	60,153	86,009

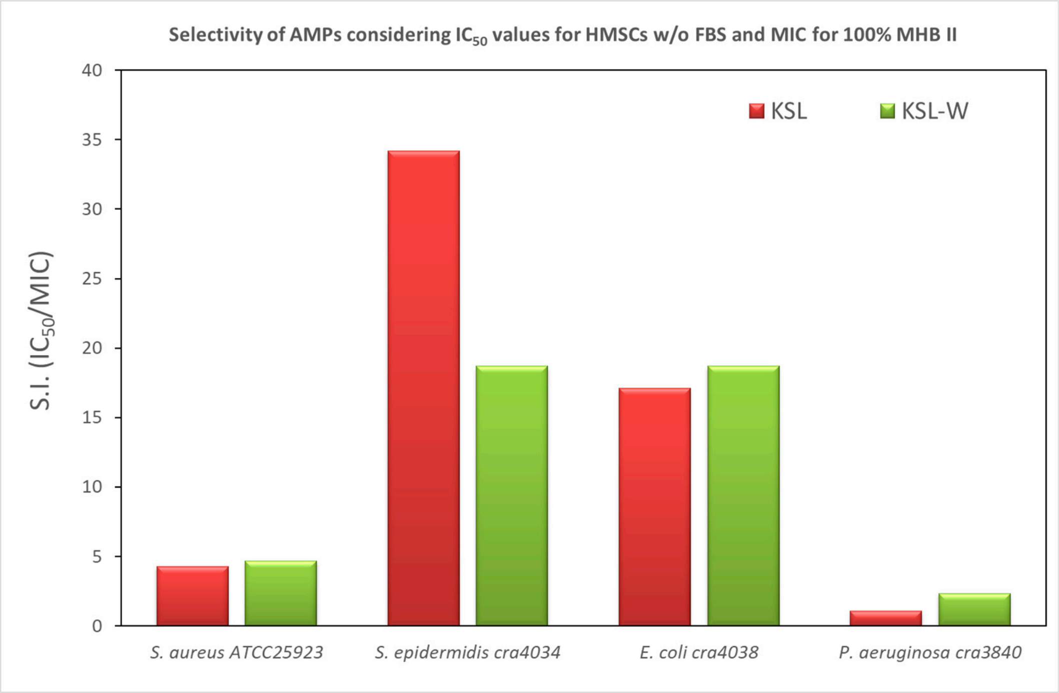

Figure S1. This graph reports the S.I. values of KSL and KSL-W for 4 representative bacterial strains considering the most stringent and challenging experimental conditions: the IC_{50} value of the most sensitive cell line hMSCs w/o FBS and with MIC tested in undiluted MHB II.

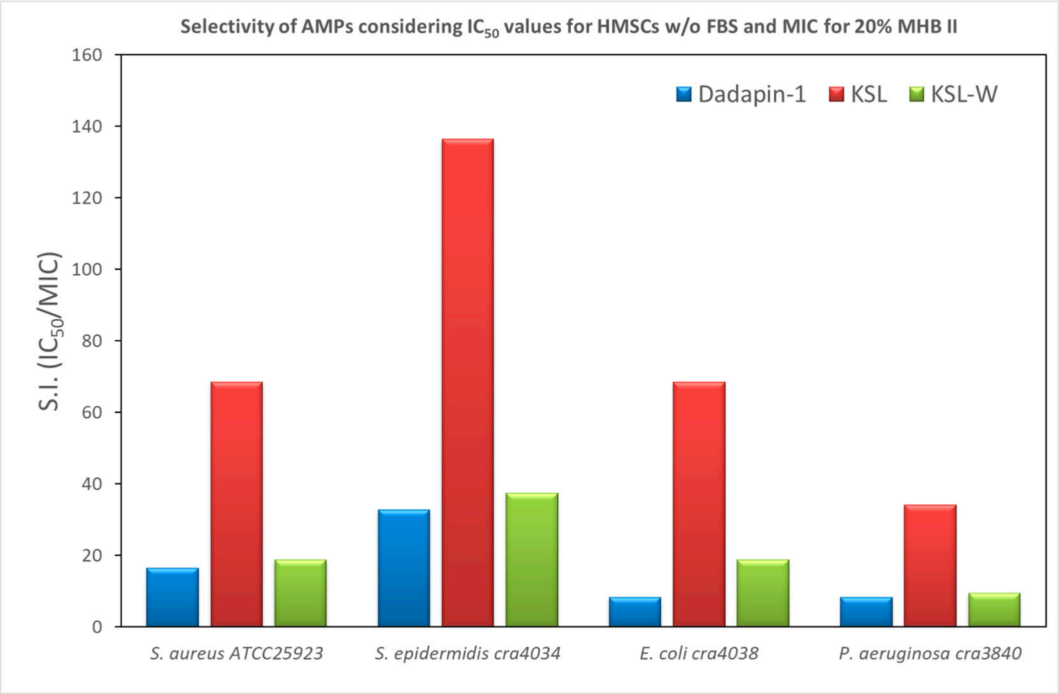

Figure S2. This graph reports the S.I. values of Dadapin-1, KSL and KSL-W for 4 representative bacterial strains considering the most stringent IC value of hMSCs w/o FBS, but with MIC tested in 20% MHB II.

ROW AND ELABORATED DATA FROM CYTOTOXICITY ANALYSIS.

Dadapin-1 tested on L929 in the presence of FBS

Well	Row	Dadapin-1 [µg/mL]	Date	Sample (RLU)	Blank (RLU)	Sample-Blank	Cell activity (%)
1	A	450.0	20/01/2023	9855840	502.5	9855337.5	82.45939
1	B	450.0	20/01/2023	11803700	502.5	11803197.5	98.75709
1	C	450.0	20/01/2023	9171670	502.5	9171167.5	76.73495
2	A	225.0	20/01/2023	12216200	502.5	12215697.5	102.20846
2	B	225.0	20/01/2023	14523700	502.5	14523197.5	121.51526
2	C	225.0	20/01/2023	13242400	502.5	13241897.5	110.79466
3	A	112.5	20/01/2023	12919400	502.5	12918897.5	108.09212
3	B	112.5	20/01/2023	14424200	502.5	14423697.5	120.68275
3	C	112.5	20/01/2023	13301300	502.5	13300797.5	111.28747
4	A	56.3	20/01/2023	13125200	502.5	13124697.5	109.81405
4	B	56.3	20/01/2023	14369900	502.5	14369397.5	120.22842
4	C	56.3	20/01/2023	13864200	502.5	13863697.5	115.99724
5	A	28.1	20/01/2023	13876200	502.5	13875697.5	116.09765
5	B	28.1	20/01/2023	14851900	502.5	14851397.5	124.26131
5	C	28.1	20/01/2023	14180900	502.5	14180397.5	118.64706
6	A	14.1	20/01/2023	14490500	502.5	14489997.5	121.23748
6	B	14.1	20/01/2023	15297300	502.5	15296797.5	127.98796
6	C	14.1	20/01/2023	14884200	502.5	14883697.5	124.53156
7	A	7.0	20/01/2023	14854800	502.5	14854297.5	124.28557
7	B	7.0	20/01/2023	15064600	502.5	15064097.5	126.04096
7	C	7.0	20/01/2023	14343600	502.5	14343097.5	120.00837

8	A	3.5	20/01/2023	14788500	502.5	14787997.5	123.73084
8	B	3.5	20/01/2023	15814700	502.5	15814197.5	132.31703
8	C	3.5	20/01/2023	14657100	502.5	14656597.5	122.63142
9	A	1.8	20/01/2023	14494000	502.5	14493497.5	121.26676
9	B	1.8	20/01/2023	15453100	502.5	15452597.5	129.29153
9	C	1.8	20/01/2023	15148100	502.5	15147597.5	126.73960
10	A	0.9	20/01/2023	14553700	502.5	14553197.5	121.76627
10	B	0.9	20/01/2023	15403700	502.5	15403197.5	128.87820
10	C	0.9	20/01/2023	14519200	502.5	14518697.5	121.47761
11	A	0.4	20/01/2023	14359600	502.5	14359097.5	120.14224
11	B	0.4	20/01/2023	15265100	502.5	15264597.5	127.71854
11	C	0.4	20/01/2023	15015400	502.5	15014897.5	125.62931
12	A	0.2	20/01/2023	15291500	502.5	15290997.5	127.93943
12	B	0.2	20/01/2023	14925300	502.5	14924797.5	124.87544
12	C	0.2	20/01/2023	14572000	502.5	14571497.5	121.91939
9	H	Blank	20/01/2023	948			
10	H	Blank	20/01/2023	310			
11	H	Blank	20/01/2023	360			
12	H	Blank	20/01/2023	392			
1	A	450	27/01/2023	9229870	577.5	9229292.5	77.33662
1	B	450	27/01/2023	10105300	577.5	10104722.5	84.67227
1	C	450	27/01/2023	10700300	577.5	10699722.5	89.65805
2	A	225	27/01/2023	11862300	577.5	11861722.5	99.39500
2	B	225	27/01/2023	13136100	577.5	13135522.5	110.06878
2	C	225	27/01/2023	13571900	577.5	13571322.5	113.72055
3	A	112.5	27/01/2023	11250400	577.5	11249822.5	94.26760
3	B	112.5	27/01/2023	13426200	577.5	13425622.5	112.49966
3	C	112.5	27/01/2023	14446500	577.5	14445922.5	121.04924

4	A	56.25	27/01/2023	12848600	577.5	12848022.5	107.65968
4	B	56.25	27/01/2023	13857400	577.5	13856822.5	116.11289
4	C	56.25	27/01/2023	13426800	577.5	13426222.5	112.50469
5	A	28.1	27/01/2023	12619000	577.5	12618422.5	105.73575
5	B	28.1	27/01/2023	13387700	577.5	13387122.5	112.17705
5	C	28.1	27/01/2023	13814200	577.5	13813622.5	115.75090
6	A	14.1	27/01/2023	13223800	577.5	13223222.5	110.80366
6	B	14.1	27/01/2023	14332800	577.5	14332222.5	120.09649
6	C	14.1	27/01/2023	15031100	577.5	15030522.5	125.94788
7	A	7.0	27/01/2023	12978100	577.5	12977522.5	108.74482
7	B	7.0	27/01/2023	15162900	577.5	15162322.5	127.05230
7	C	7.0	27/01/2023	14785500	577.5	14784922.5	123.88988
8	A	3.5	27/01/2023	13089200	577.5	13088622.5	109.67578
8	B	3.5	27/01/2023	14324900	577.5	14324322.5	120.03030
8	C	3.5	27/01/2023	14896600	577.5	14896022.5	124.82084
9	A	1.8	27/01/2023	12978700	577.5	12978122.5	108.74985
9	B	1.8	27/01/2023	14545500	577.5	14544922.5	121.87881
9	C	1.8	27/01/2023	15196700	577.5	15196122.5	127.33552
10	A	0.9	27/01/2023	13665500	577.5	13664922.5	114.50487
10	B	0.9	27/01/2023	13487900	577.5	13487322.5	113.01668
10	C	0.9	27/01/2023	14528900	577.5	14528322.5	121.73971
11	A	0.4	27/01/2023	12893900	577.5	12893322.5	108.03927
11	B	0.4	27/01/2023	14597400	577.5	14596822.5	122.31370
11	C	0.4	27/01/2023	14966100	577.5	14965522.5	125.40322
12	A	0.2	27/01/2023	12941400	577.5	12940822.5	108.43729
12	B	0.2	27/01/2023	14730000	577.5	14729422.5	123.42482
12	C	0.2	27/01/2023	14160100	577.5	14159522.5	118.64936
9	H	Blank	27/01/2023	1122			

10	H	Blank	27/01/2023	318			
11	H	Blank	27/01/2023	358			
12	H	Blank	27/01/2023	512			
1	A	450	01/02/2023	9576790	565.5	9576224.5	71.93727
1	B	450	01/02/2023	10532500	565.5	10531934.5	79.11663
1	C	450	01/02/2023	9455600	565.5	9455034.5	71.02688
2	A	225	01/02/2023	12496900	565.5	12496334.5	93.87334
2	B	225	01/02/2023	14579400	565.5	14578834.5	109.51723
2	C	225	01/02/2023	14115400	565.5	14114834.5	106.03163
3	A	112.5	01/02/2023	13844000	565.5	13843434.5	103.99285
3	B	112.5	01/02/2023	14602300	565.5	14601734.5	109.68926
3	C	112.5	01/02/2023	14375200	565.5	14374634.5	107.98326
4	A	56.25	01/02/2023	13363100	565.5	13362534.5	100.38030
4	B	56.25	01/02/2023	15562900	565.5	15562334.5	116.90535
4	C	56.25	01/02/2023	14972500	565.5	14971934.5	112.47022
5	A	28.1	01/02/2023	14740200	565.5	14739634.5	110.72517
5	B	28.1	01/02/2023	16179900	565.5	16179334.5	121.54030
5	C	28.1	01/02/2023	15707300	565.5	15706734.5	117.99009
6	A	14.1	01/02/2023	13796400	565.5	13795834.5	103.63528
6	B	14.1	01/02/2023	15832800	565.5	15832234.5	118.93286
6	C	14.1	01/02/2023	15701600	565.5	15701034.5	117.94727
7	A	7.0	01/02/2023	15179500	565.5	15178934.5	114.02522
7	B	7.0	01/02/2023	16597000	565.5	16596434.5	124.67358
7	C	7.0	01/02/2023	15897100	565.5	15896534.5	119.41588
8	A	3.5	01/02/2023	14179200	565.5	14178634.5	106.51090
8	B	3.5	01/02/2023	14448500	565.5	14447934.5	108.53390
8	C	3.5	01/02/2023	15559100	565.5	15558534.5	116.87680
9	A	1.8	01/02/2023	14347100	565.5	14346534.5	107.77218

9	B	1.8	01/02/2023	14906000	565.5	14905434.5	111.97067
9	C	1.8	01/02/2023	15256900	565.5	15256334.5	114.60666
10	A	0.9	01/02/2023	14280400	565.5	14279834.5	107.27112
10	B	0.9	01/02/2023	14602700	565.5	14602134.5	109.69226
10	C	0.9	01/02/2023	15279000	565.5	15278434.5	114.77267
11	A	0.4	01/02/2023	13022700	565.5	13022134.5	97.82319
11	B	0.4	01/02/2023	14719800	565.5	14719234.5	110.57192
11	C	0.4	01/02/2023	14483900	565.5	14483334.5	108.79983
12	A	0.2	01/02/2023	12153500	565.5	12152934.5	91.29370
12	B	0.2	01/02/2023	13918400	565.5	13917834.5	104.55175
12	C	0.2	01/02/2023	13772700	565.5	13772134.5	103.45724
9	H	Blank	01/02/2023	1204			
10	H	Blank	01/02/2023	344			
11	H	Blank	01/02/2023	316			
12	H	Blank	01/02/2023	398			
1	G	**Reference Control**	20/01/2023	11129700	502.5	11129197.5	93.11774
2	G		20/01/2023	11814300	502.5	11813797.5	98.84578
3	G		20/01/2023	12277700	502.5	12277197.5	102.72303
4	G		20/01/2023	12587300	502.5	12586797.5	105.31345
1	G		27/01/2023	10760700	577.5	10760122.5	90.16417
2	G		27/01/2023	11638200	577.5	11637622.5	97.51716
3	G		27/01/2023	12300800	577.5	12300222.5	103.06940
4	G		27/01/2023	13038300	577.5	13037722.5	109.24926
1	G		01/02/2023	12349500	565.5	12348934.5	92.76606
2	G		01/02/2023	13490900	565.5	13490334.5	101.34034
3	G		01/02/2023	13697500	565.5	13696934.5	102.89233
4	G		01/02/2023	13712000	565.5	13711434.5	103.00126

5 G	**Medium Control**	20/01/2023	13034800	502.5	13034297.5	109.05767
6 G		20/01/2023	12590500	502.5	12589997.5	105.34022
7 G		20/01/2023	12382700	502.5	12382197.5	103.60157
8 G		20/01/2023	12960800	502.5	12960297.5	108.43851
5 G		27/01/2023	12460300	577.5	12459722.5	104.40593
6 G		27/01/2023	13120500	577.5	13119922.5	109.93806
7 G		27/01/2023	12896600	577.5	12896022.5	108.06189
8 G		27/01/2023	12581500	577.5	12580922.5	105.42152
5 G		01/02/2023	13573800	565.5	13573234.5	101.96309
6 G		01/02/2023	14818600	565.5	14818034.5	111.31412
7 G		01/02/2023	14976900	565.5	14976334.5	112.50328
8 G		01/02/2023	15141800	565.5	15141234.5	113.74202
9 G	**Positive Control**	20/01/2023	8885	502.5	8382.7	0.07014
10 G		20/01/2023	5845	502.5	5342.9	0.04470
11 G		20/01/2023	6084	502.5	5581.0	0.04670
12 G		20/01/2023	4265	502.5	3762.2	0.03148
9 G		27/01/2023	8915	577.5	8337.7	0.06987
10 G		27/01/2023	6368	577.5	5790.1	0.04852
11 G		27/01/2023	5939	577.5	5361.9	0.04493
12 G		27/01/2023	4505	577.5	3927.3	0.03291
9 G		01/02/2023	9167	565.5	8601.9	0.06462
10 G		01/02/2023	5251	565.5	4685.6	0.03520
11 G		01/02/2023	5775	565.5	5209.9	0.03914
12 G		01/02/2023	3923	565.5	3357.1	0.02522

Dadapin-1 tested on L929 in the absence of FBS

Well	Row	Dadapin-1 [µg/mL]	Date	Sample (RLU)	Blank (RLU)	Sample - Blank	Cell activity (%)
1	A	450.0	22/12/2022	7006070	225.5	7005844.5	106.75134
1	B	450.0	22/12/2022	10207800	225.5	10207574.5	155.53761
1	C	450.0	22/12/2022	7050120	225.5	7049894.5	107.42255
2	A	225.0	22/12/2022	9711900	225.5	9711674.5	147.98134
2	B	225.0	22/12/2022	8673500	225.5	8673274.5	132.15876
2	C	225.0	22/12/2022	8050710	225.5	8050484.5	122.66901
3	A	112.5	22/12/2022	9850460	225.5	9850234.5	150.09265
3	B	112.5	22/12/2022	8713990	225.5	8713764.5	132.77572
3	C	112.5	22/12/2022	7880340	225.5	7880114.5	120.07300
4	A	56.3	22/12/2022	10581100	225.5	10580874.5	161.22575
4	B	56.3	22/12/2022	10444700	225.5	10444474.5	159.14736
4	C	56.3	22/12/2022	7723340	225.5	7723114.5	117.68072
5	A	28.1	22/12/2022	10520200	225.5	10519974.5	160.29779
5	B	28.1	22/12/2022	11232500	225.5	11232274.5	171.15144
5	C	28.1	22/12/2022	8024220	225.5	8023994.5	122.26537
6	A	14.1	22/12/2022	10010000	225.5	10009774.5	152.52363
6	B	14.1	22/12/2022	10510300	225.5	10510074.5	160.14694
6	C	14.1	22/12/2022	8479030	225.5	8478804.5	129.19552
7	A	7.0	22/12/2022	8896660	225.5	8896434.5	135.55915
7	B	7.0	22/12/2022	11285900	225.5	11285674.5	171.96512
7	C	7.0	22/12/2022	8368760	225.5	8368534.5	127.51529
8	A	3.5	22/12/2022	9601190	225.5	9600964.5	146.29440
8	B	3.5	22/12/2022	9240880	225.5	9240654.5	140.80419

8	C	3.5	22/12/2022	7912770	225.5	7912544.5	120.56716
9	A	1.8	22/12/2022	8883830	225.5	8883604.5	135.36365
9	B	1.8	22/12/2022	8699180	225.5	8698954.5	132.55005
9	C	1.8	22/12/2022	9702000	225.5	9701774.5	147.83049
10	A	0.9	22/12/2022	8435320	225.5	8435094.5	128.52950
10	B	0.9	22/12/2022	8449560	225.5	8449334.5	128.74648
10	C	0.9	22/12/2022	8088420	225.5	8088194.5	123.24362
11	A	0.4	22/12/2022	7920210	225.5	7919984.5	120.68052
11	B	0.4	22/12/2022	5827930	225.5	5827704.5	88.79947
11	C	0.4	22/12/2022	7614480	225.5	7614254.5	116.02197
12	A	0.2	22/12/2022	5828070	225.5	5827844.5	88.80160
12	B	0.2	22/12/2022	4641570	225.5	4641344.5	70.72235
12	C	0.2	22/12/2022	7612680	225.5	7612454.5	115.99454
9	H	Blank	22/12/2022	602			
10	H	Blank	22/12/2022	92			
11	H	Blank	22/12/2022	106			
12	H	Blank	22/12/2022	102			
1	A	450	23/12/2022	8851650	302.0	8851348.0	97.69462
1	B	450	23/12/2022	8968380	302.0	8968078.0	98.98300
1	C	450	23/12/2022	10175700	302.0	10175398.0	112.30850
2	A	225	23/12/2022	10154500	302.0	10154198.0	112.07451
2	B	225	23/12/2022	9801200	302.0	9800898.0	108.17505
2	C	225	23/12/2022	10319600	302.0	10319298.0	113.89676
3	A	112.5	23/12/2022	10697000	302.0	10696698.0	118.06223
3	B	112.5	23/12/2022	9373610	302.0	9373308.0	103.45563
3	C	112.5	23/12/2022	9889260	302.0	9888958.0	109.14699
4	A	56.25	23/12/2022	11605200	302.0	11604898.0	128.08626
4	B	56.25	23/12/2022	10613300	302.0	10612998.0	117.13841

4	C	56.25	23/12/2022	10559300	302.0	10558998.0	116.54240
5	A	28.1	23/12/2022	11902200	302.0	11901898.0	131.36433
5	B	28.1	23/12/2022	10049300	302.0	10048998.0	110.91339
5	C	28.1	23/12/2022	11202800	302.0	11202498.0	123.64487
6	A	14.1	23/12/2022	12061000	302.0	12060698.0	133.11705
6	B	14.1	23/12/2022	11861200	302.0	11860898.0	130.91180
6	C	14.1	23/12/2022	12708900	302.0	12708598.0	140.26809
7	A	7.0	23/12/2022	11657300	302.0	11656998.0	128.66131
7	B	7.0	23/12/2022	10377000	302.0	10376698.0	114.53030
7	C	7.0	23/12/2022	12101800	302.0	12101498.0	133.56737
8	A	3.5	23/12/2022	12278900	302.0	12278598.0	135.52207
8	B	3.5	23/12/2022	10469700	302.0	10469398.0	115.55346
8	C	3.5	23/12/2022	12297300	302.0	12296998.0	135.72515
9	A	1.8	23/12/2022	12060100	302.0	12059798.0	133.10711
9	B	1.8	23/12/2022	10983600	302.0	10983298.0	121.22550
9	C	1.8	23/12/2022	11871200	302.0	11870898.0	131.02218
10	A	0.9	23/12/2022	11818400	302.0	11818098.0	130.43941
10	B	0.9	23/12/2022	9280060	302.0	9279758.0	102.42309
10	C	0.9	23/12/2022	11590200	302.0	11589898.0	127.92071
11	A	0.4	23/12/2022	10862800	302.0	10862498.0	119.89220
11	B	0.4	23/12/2022	8497960	302.0	8497658.0	93.79085
11	C	0.4	23/12/2022	11203300	302.0	11202998.0	123.65039
12	A	0.2	23/12/2022	10807900	302.0	10807598.0	119.28626
12	B	0.2	23/12/2022	9575250	302.0	9574948.0	105.68118
12	C	0.2	23/12/2022	10872700	302.0	10872398.0	120.00147
9	H	Blank	23/12/2022	726			
10	H	Blank	23/12/2022	104			
11	H	Blank	23/12/2022	128			

12	H	Blank	23/12/2022	250			
1	A	450.0	15/02/2023	7475810	2920.4	7472889.6	84.49689
1	B	450.0	15/02/2023	8707180	2920.4	8704259.6	98.42014
1	C	450.0	15/02/2023	8672030	2920.4	8669109.6	98.02270
2	A	225.0	15/02/2023	7687660	2920.4	7684739.6	86.89230
2	B	225.0	15/02/2023	9733040	2920.4	9730119.6	110.01967
2	C	225.0	15/02/2023	9070190	2920.4	9067269.6	102.52474
3	A	112.5	15/02/2023	8326980	2920.4	8324059.6	94.12117
3	B	112.5	15/02/2023	9483160	2920.4	9480239.6	107.19424
3	C	112.5	15/02/2023	10593000	2920.4	10590079.6	119.74334
4	A	56.3	15/02/2023	8233120	2920.4	8230199.6	93.05989
4	B	56.3	15/02/2023	9225470	2920.4	9222549.6	104.28051
4	C	56.3	15/02/2023	10015900	2920.4	10012979.6	113.21800
5	A	28.1	15/02/2023	6199040	2920.4	6196119.6	70.06029
5	B	28.1	15/02/2023	8294990	2920.4	8292069.6	93.75946
5	C	28.1	15/02/2023	9728670	2920.4	9725749.6	109.97025
6	A	14.1	15/02/2023	6078630	2920.4	6075709.6	68.69880
6	B	14.1	15/02/2023	8612210	2920.4	8609289.6	97.34630
6	C	14.1	15/02/2023	9568750	2920.4	9565829.6	108.16202
7	A	7.0	15/02/2023	7021610	2920.4	7018689.6	79.36119
7	B	7.0	15/02/2023	8316090	2920.4	8313169.6	93.99804
7	C	7.0	15/02/2023	10288900	2920.4	10285979.6	116.30484
8	A	3.5	15/02/2023	7393130	2920.4	7390209.6	83.56201
8	B	3.5	15/02/2023	8316440	2920.4	8313519.6	94.00199
8	C	3.5	15/02/2023	10142900	2920.4	10139979.6	114.65400
9	A	1.8	15/02/2023	6255440	2920.4	6252519.6	70.69801
9	B	1.8	15/02/2023	7909130	2920.4	7906209.6	89.39649
9	C	1.8	15/02/2023	11266900	2920.4	11263979.6	127.36321

10	A	0.9	15/02/2023	6414640	2920.4	6411719.6	72.49811
10	B	0.9	15/02/2023	9822590	2920.4	9819669.6	111.03222
10	C	0.9	15/02/2023	9839710	2920.4	9836789.6	111.22580
11	A	0.4	15/02/2023	7833090	2920.4	7830169.6	88.53670
11	B	0.4	15/02/2023	9417430	2920.4	9414509.6	106.45103
11	C	0.4	15/02/2023	10519400	2920.4	10516479.6	118.91114
12	A	0.2	15/02/2023	8167900	2920.4	8164979.6	92.32243
12	B	0.2	15/02/2023	7791520	2920.4	7788599.6	88.06666
12	C	0.2	15/02/2023	10564000	2920.4	10561079.6	119.41543
1	H	Blank	15/02/2023	2406			
2	H	Blank	15/02/2023	3346			
3	H	Blank	15/02/2023	2928			
4	H	Blank	15/02/2023	3000			
1	A	450.0	22/02/2023	8670310	297.5	8670012.5	103.61796
1	B	450.0	22/02/2023	9825610	297.5	9825312.5	117.42530
1	C	450.0	22/02/2023	9020230	297.5	9019932.5	107.79996
2	A	225.0	22/02/2023	9205410	297.5	9205112.5	110.01310
2	B	225.0	22/02/2023	8805140	297.5	8804842.5	105.22935
2	C	225.0	22/02/2023	9193910	297.5	9193612.5	109.87566
3	A	112.5	22/02/2023	8384160	297.5	8383862.5	100.19809
3	B	112.5	22/02/2023	9948470	297.5	9948172.5	118.89364
3	C	112.5	22/02/2023	8915230	297.5	8914932.5	106.54507
4	A	56.3	22/02/2023	7293960	297.5	7293662.5	87.16878
4	B	56.3	22/02/2023	11280900	297.5	11280602.5	134.81791
4	C	56.3	22/02/2023	8754520	297.5	8754222.5	104.62438
5	A	28.1	22/02/2023	8869420	297.5	8869122.5	105.99758
5	B	28.1	22/02/2023	8731600	297.5	8731302.5	104.35045
5	C	28.1	22/02/2023	8337810	297.5	8337512.5	99.64415

6	A	14.1	22/02/2023	8408470	297.5	8408172.5	100.48863
6	B	14.1	22/02/2023	8900070	297.5	8899772.5	106.36389
6	C	14.1	22/02/2023	8946140	297.5	8945842.5	106.91449
7	A	7.0	22/02/2023	8085620	297.5	8085322.5	96.63015
7	B	7.0	22/02/2023	9419970	297.5	9419672.5	112.57737
7	C	7.0	22/02/2023	8543530	297.5	8543232.5	102.10277
8	A	3.5	22/02/2023	7776810	297.5	7776512.5	92.93947
8	B	3.5	22/02/2023	7758200	297.5	7757902.5	92.71705
8	C	3.5	22/02/2023	7996570	297.5	7996272.5	95.56589
9	A	1.8	22/02/2023	8383870	297.5	8383572.5	100.19463
9	B	1.8	22/02/2023	8497330	297.5	8497032.5	101.55062
9	C	1.8	22/02/2023	8771850	297.5	8771552.5	104.83149
10	A	0.9	22/02/2023	7547380	297.5	7547082.5	90.19748
10	B	0.9	22/02/2023	8879590	297.5	8879292.5	106.11913
10	C	0.9	22/02/2023	9555840	297.5	9555542.5	114.20120
11	A	0.4	22/02/2023	8790840	297.5	8790542.5	105.05845
11	B	0.4	22/02/2023	8543730	297.5	8543432.5	102.10516
11	C	0.4	22/02/2023	9330100	297.5	9329802.5	111.50331
12	A	0.2	22/02/2023	8536330	297.5	8536032.5	102.01672
12	B	0.2	22/02/2023	8709230	297.5	8708932.5	104.08310
12	C	0.2	22/02/2023	9145240	297.5	9144942.5	109.29399
9	H	Blank	22/02/2023	560			
10	H	Blank	22/02/2023	146			
11	H	Blank	22/02/2023	172			
12	H	Blank	22/02/2023	312			
1	G	**Reference Control**	22/12/2022	6049770	225.5	6049544.5	92.17975
2	G		22/12/2022	6558380	225.5	6558154.5	99.92968
3	G		22/12/2022	6922620	225.5	6922394.5	105.47977

4 G		22/12/2022	6721210	225.5	6720984.5	102.41080
1 G		23/12/2022	8283860	302.0	8283558.0	91.42777
2 G		23/12/2022	8793720	302.0	8793418.0	97.05523
3 G		23/12/2022	9080510	302.0	9080208.0	100.22061
4 G		23/12/2022	10084000	302.0	10083698.0	111.29639
1 G		15/02/2023	8910860	2920.4	8907939.6	100.72318
2 G		15/02/2023	9187730	2920.4	9184809.6	103.85378
3 G		15/02/2023	8653820	2920.4	8650899.6	97.81679
4 G		15/02/2023	8635200	2920.4	8632279.6	97.60625
1 G		22/02/2023	9308570	297.5	9308272.5	111.24600
2 G		22/02/2023	8189040	297.5	8188742.5	97.86615
3 G		22/02/2023	7974150	297.5	7973852.5	95.29794
4 G		22/02/2023	7998580	297.5	7998282.5	95.58991
5 G	**Medium Control**	22/12/2022	6158990	225.5	6158764.5	93.84399
6 G		22/12/2022	6976830	225.5	6976604.5	106.30580
7 G		22/12/2022	6869690	225.5	6869464.5	104.67326
8 G		22/12/2022	8355960	225.5	8355734.5	127.32025
5 G		23/12/2022	9046660	302.0	9046358.0	99.84700
6 G		23/12/2022	9744760	302.0	9744458.0	107.55211
7 G		23/12/2022	9602390	302.0	9602088.0	105.98073
8 G		23/12/2022	9348470	302.0	9348168.0	103.17815
5 G		15/02/2023	7533430	2920.4	7530509.6	85.14840
6 G		15/02/2023	7636450	2920.4	7633529.6	86.31326
7 G		15/02/2023	8916250	2920.4	8913329.6	100.78412
8 G		15/02/2023	8308180	2920.4	8305259.6	93.90860
5 G		22/02/2023	7275780	297.5	7275482.5	86.95151
6 G		22/02/2023	8484160	297.5	8483862.5	101.39322
7 G		22/02/2023	7260380	297.5	7260082.5	86.76746

8	G		22/02/2023	6928450	297.5	6928152.5	82.80046
9	G	**Positive Control**	22/12/2022	11862	225.5	11636.3	0.17731
10	G		22/12/2022	11950	225.5	11724.4	0.17865
11	G		22/12/2022	9676	225.5	9450.3	0.14400
12	G		22/12/2022	8605	225.5	8379.5	0.12768
9	G		23/12/2022	14489	302.0	14186.6	0.15658
10	G		23/12/2022	21593	302.0	21291.1	0.23500
11	G		23/12/2022	13868	302.0	13565.9	0.14973
12	G		23/12/2022	11588	302.0	11285.5	0.12456
9	G		15/02/2023	28804	2920.4	25883.6	0.29267
10	G		15/02/2023	21158	2920.4	18237.9	0.20622
11	G		15/02/2023	18456	2920.4	15535.6	0.17566
12	G		15/02/2023	18706	2920.4	15785.9	0.17849
9	G		22/02/2023	6712	297.5	6414.3	0.07666
10	G		22/02/2023	9205	297.5	8908.0	0.10646
11	G		22/02/2023	3817	297.5	3519.1	0.04206
12	G		22/02/2023	3412	297.5	3115.0	0.03723

Dadapin-1 tested on hMSCs in the presence of FBS

Well	Row	Dadapin-1 [µg/mL]	Date	Sample (RLU)	Blank (RLU)	Sample-Blank	Cell activity (%)
1	A	450.0	24/05/2023	2363050	456.5	2362593.5	41.75196
1	B	450.0	24/05/2023	2346110	456.5	2345653.5	41.45259
1	C	450.0	24/05/2023	2462530	456.5	2462073.5	43.50998
2	A	225.0	24/05/2023	5020560	456.5	5020103.5	88.71571
2	B	225.0	24/05/2023	5487100	456.5	5486643.5	96.96045

2	C	225.0	24/05/2023	5318900	456.5	5318443.5	93.98800
3	A	112.5	24/05/2023	5960870	456.5	5960413.5	105.33295
3	B	112.5	24/05/2023	6183450	456.5	6182993.5	109.26640
3	C	112.5	24/05/2023	6470240	456.5	6469783.5	114.33458
4	A	56.3	24/05/2023	6109650	456.5	6109193.5	107.96220
4	B	56.3	24/05/2023	6651790	456.5	6651333.5	117.54295
4	C	56.3	24/05/2023	6573380	456.5	6572923.5	116.15728
5	A	28.1	24/05/2023	5841250	456.5	5840793.5	103.21901
5	B	28.1	24/05/2023	6399400	456.5	6398943.5	113.08269
5	C	28.1	24/05/2023	6208310	456.5	6207853.5	109.70573
6	A	14.1	24/05/2023	5804170	456.5	5803713.5	102.56373
6	B	14.1	24/05/2023	6360800	456.5	6360343.5	112.40055
6	C	14.1	24/05/2023	6256680	456.5	6256223.5	110.56053
7	A	7.0	24/05/2023	5692280	456.5	5691823.5	100.58640
7	B	7.0	24/05/2023	6346170	456.5	6345713.5	112.14201
7	C	7.0	24/05/2023	6314130	456.5	6313673.5	111.57579
8	A	3.5	24/05/2023	5490750	456.5	5490293.5	97.02495
8	B	3.5	24/05/2023	6214100	456.5	6213643.5	109.80805
8	C	3.5	24/05/2023	5784470	456.5	5784013.5	102.21559
9	A	1.8	24/05/2023	5711160	456.5	5710703.5	100.92005
9	B	1.8	24/05/2023	6342800	456.5	6342343.5	112.08245
9	C	1.8	24/05/2023	6220610	456.5	6220153.5	109.92310
10	A	0.9	24/05/2023	5651430	456.5	5650973.5	99.86450
10	B	0.9	24/05/2023	6488780	456.5	6488323.5	114.66222
10	C	0.9	24/05/2023	6468390	456.5	6467933.5	114.30189
11	A	0.4	24/05/2023	5330900	456.5	5330443.5	94.20006
11	B	0.4	24/05/2023	6341960	456.5	6341503.5	112.06761
11	C	0.4	24/05/2023	5856940	456.5	5856483.5	103.49629

12	A	0.2	24/05/2023	5386340	456.5	5385883.5	95.17981
12	B	0.2	24/05/2023	5933380	456.5	5932923.5	104.84714
12	C	0.2	24/05/2023	6006710	456.5	6006253.5	106.14304
9	H	Blank	24/05/2023	806			
10	H	Blank	24/05/2023	332			
11	H	Blank	24/05/2023	344			
12	H	Blank	24/05/2023	344			
1	A	450	25/05/2023	1733720	419.0	1733301.0	33.49027
1	B	450	25/05/2023	1916110	419.0	1915691.0	37.01435
1	C	450	25/05/2023	1645000	419.0	1644581.0	31.77605
2	A	225	25/05/2023	3905860	419.0	3905441.0	75.45964
2	B	225	25/05/2023	4167660	419.0	4167241.0	80.51806
2	C	225	25/05/2023	3813620	419.0	3813201.0	73.67741
3	A	112.5	25/05/2023	4820540	419.0	4820121.0	93.13279
3	B	112.5	25/05/2023	5203800	419.0	5203381.0	100.53801
3	C	112.5	25/05/2023	5043370	419.0	5042951.0	97.43824
4	A	56.25	25/05/2023	4698240	419.0	4697821.0	90.76975
4	B	56.25	25/05/2023	5320180	419.0	5319761.0	102.78667
4	C	56.25	25/05/2023	4776070	419.0	4775651.0	92.27355
5	A	28.1	25/05/2023	4746670	419.0	4746251.0	91.70550
5	B	28.1	25/05/2023	4981680	419.0	4981261.0	96.24628
5	C	28.1	25/05/2023	4749890	419.0	4749471.0	91.76771
6	A	14.1	25/05/2023	4357900	419.0	4357481.0	84.19381
6	B	14.1	25/05/2023	4932120	419.0	4931701.0	95.28870
6	C	14.1	25/05/2023	4396830	419.0	4396411.0	84.94600
7	A	7.0	25/05/2023	4649530	419.0	4649111.0	89.82859
7	B	7.0	25/05/2023	5145100	419.0	5144681.0	99.40383
7	C	7.0	25/05/2023	4707830	419.0	4707411.0	90.95504

8	A	3.5	25/05/2023	4333330	419.0	4332911.0	83.71908
8	B	3.5	25/05/2023	4685620	419.0	4685201.0	90.52591
8	C	3.5	25/05/2023	4649090	419.0	4648671.0	89.82009
9	A	1.8	25/05/2023	4419070	419.0	4418651.0	85.37572
9	B	1.8	25/05/2023	5268250	419.0	5267831.0	101.78329
9	C	1.8	25/05/2023	4742110	419.0	4741691.0	91.61739
10	A	0.9	25/05/2023	4631150	419.0	4630731.0	89.47346
10	B	0.9	25/05/2023	5283150	419.0	5282731.0	102.07119
10	C	0.9	25/05/2023	4673910	419.0	4673491.0	90.29965
11	A	0.4	25/05/2023	4200530	419.0	4200111.0	81.15316
11	B	0.4	25/05/2023	4739450	419.0	4739031.0	91.56599
11	C	0.4	25/05/2023	4471860	419.0	4471441.0	86.39571
12	A	0.2	25/05/2023	4080570	419.0	4080151.0	78.83533
12	B	0.2	25/05/2023	4485030	419.0	4484611.0	86.65017
12	C	0.2	25/05/2023	3930320	419.0	3929901.0	75.93225
9	H	Blank	25/05/2023	646			
10	H	Blank	25/05/2023	298			
11	H	Blank	25/05/2023	300			
12	H	Blank	25/05/2023	432			
1	A	450	26/05/2023	2447940	382.5	2447557.5	45.86653
1	B	450	26/05/2023	2851070	382.5	2850687.5	53.42108
1	C	450	26/05/2023	2723530	382.5	2723147.5	51.03101
2	A	225	26/05/2023	5260140	382.5	5259757.5	98.56636
2	B	225	26/05/2023	6110750	382.5	6110367.5	114.50656
2	C	225	26/05/2023	5375380	382.5	5374997.5	100.72593
3	A	112.5	26/05/2023	5666250	382.5	5665867.5	106.17675
3	B	112.5	26/05/2023	6698850	382.5	6698467.5	125.52738
3	C	112.5	26/05/2023	6186960	382.5	6186577.5	115.93471

4	A	56.25	26/05/2023	5397420	382.5	5397037.5	101.13895
4	B	56.25	26/05/2023	6009870	382.5	6009487.5	112.61609
4	C	56.25	26/05/2023	6228470	382.5	6228087.5	116.71259
5	A	28.1	26/05/2023	5729010	382.5	5728627.5	107.35286
5	B	28.1	26/05/2023	5626650	382.5	5626267.5	105.43466
5	C	28.1	26/05/2023	5952780	382.5	5952397.5	111.54624
6	A	14.1	26/05/2023	5419390	382.5	5419007.5	101.55066
6	B	14.1	26/05/2023	5825930	382.5	5825547.5	109.16911
6	C	14.1	26/05/2023	6033120	382.5	6032737.5	113.05179
7	A	7.0	26/05/2023	5508650	382.5	5508267.5	103.22337
7	B	7.0	26/05/2023	5520500	382.5	5520117.5	103.44544
7	C	7.0	26/05/2023	6128570	382.5	6128187.5	114.84050
8	A	3.5	26/05/2023	5873930	382.5	5873547.5	110.06862
8	B	3.5	26/05/2023	6374670	382.5	6374287.5	119.45234
8	C	3.5	26/05/2023	6050260	382.5	6049877.5	113.37299
9	A	1.8	26/05/2023	5633120	382.5	5632737.5	105.55590
9	B	1.8	26/05/2023	5868030	382.5	5867647.5	109.95805
9	C	1.8	26/05/2023	5927600	382.5	5927217.5	111.07438
10	A	0.9	26/05/2023	5530270	382.5	5529887.5	103.62852
10	B	0.9	26/05/2023	5988100	382.5	5987717.5	112.20813
10	C	0.9	26/05/2023	5854540	382.5	5854157.5	109.70525
11	A	0.4	26/05/2023	5591680	382.5	5591297.5	104.77933
11	B	0.4	26/05/2023	6145590	382.5	6145207.5	115.15945
11	C	0.4	26/05/2023	5962710	382.5	5962327.5	111.73233
12	A	0.2	26/05/2023	5447090	382.5	5446707.5	102.06975
12	B	0.2	26/05/2023	6251570	382.5	6251187.5	117.14548
12	C	0.2	26/05/2023	5764690	382.5	5764307.5	108.02149
9	H	Blank	26/05/2023	738			

10	H	Blank	26/05/2023	208			
11	H	Blank	26/05/2023	268			
12	H	Blank	26/05/2023	316			
1	G	**Reference Control**	24/05/2023	5229020	456.5	5228563.5	92.39963
2	G		24/05/2023	5625030	456.5	5624573.5	99.39796
3	G		24/05/2023	5803640	456.5	5803183.5	102.55437
4	G		24/05/2023	5978700	456.5	5978243.5	105.64804
1	G		25/05/2023	4885370	419.0	4884951.0	94.38541
2	G		25/05/2023	5253760	419.0	5253341.0	101.50332
3	G		25/05/2023	5225210	419.0	5224791.0	100.95169
4	G		25/05/2023	5339480	419.0	5339061.0	103.15958
1	G		26/05/2023	4770750	382.5	4770367.5	89.39533
2	G		26/05/2023	4917180	382.5	4916797.5	92.13939
3	G		26/05/2023	5752100	382.5	5751717.5	107.78556
4	G		26/05/2023	5906540	382.5	5906157.5	110.67972
5	G	**Medium Control**	24/05/2023	6299950	456.5	6299493.5	111.32520
6	G		24/05/2023	6215460	456.5	6215003.5	109.83209
7	G		24/05/2023	6327390	456.5	6326933.5	111.81012
8	G		24/05/2023	6436730	456.5	6436273.5	113.74239
5	G		25/05/2023	5365910	419.0	5365491.0	103.67025
6	G		25/05/2023	5498870	419.0	5498451.0	106.23926
7	G		25/05/2023	5310440	419.0	5310021.0	102.59847
8	G		25/05/2023	5304820	419.0	5304401.0	102.48989
5	G		26/05/2023	6136390	382.5	6136007.5	114.98704
6	G		26/05/2023	5845030	382.5	5844647.5	109.52704
7	G		26/05/2023	6076740	382.5	6076357.5	113.86922
8	G		26/05/2023	5979410	382.5	5979027.5	112.04528

9	G	**Positive Control**	24/05/2023	4969	456.5	4512.5	0.07975
10	G		24/05/2023	3567	456.5	3110.0	0.05496
11	G		24/05/2023	3282	456.5	2825.9	0.04994
12	G		24/05/2023	2658	456.5	2201.8	0.03891
9	G		25/05/2023	3985	419.0	3565.6	0.06889
10	G		25/05/2023	2696	419.0	2277.3	0.04400
11	G		25/05/2023	2596	419.0	2177.3	0.04207
12	G		25/05/2023	1914	419.0	1495.1	0.02889
9	G		26/05/2023	4313	382.5	3930.3	0.07365
10	G		26/05/2023	3018	382.5	2635.9	0.04940
11	G		26/05/2023	2738	382.5	2355.8	0.04415
12	G		26/05/2023	2078	382.5	1695.7	0.03178

Dadapin-1 tested on hMSCs in the absence of FBS

Well	Row	Dadapin-1 [µg/mL]	Date	Sample (RLU)	Blank (RLU)	Sample-Blank	Cell activity (%)
1	A	450.0	31/05/2023	292336	149.5	292186.5	7.47643
1	B	450.0	31/05/2023	318836	149.5	318686.5	8.15451
1	C	450.0	31/05/2023	422434	149.5	422284.5	10.80536
2	A	225.0	31/05/2023	1997220	149.5	1997070.5	51.10076
2	B	225.0	31/05/2023	2138410	149.5	2138260.5	54.71351
2	C	225.0	31/05/2023	2179770	149.5	2179620.5	55.77183
3	A	112.5	31/05/2023	3768240	149.5	3768090.5	96.41738
3	B	112.5	31/05/2023	4100740	149.5	4100590.5	104.92534
3	C	112.5	31/05/2023	4404790	149.5	4404640.5	112.70533

4	A	56.3	31/05/2023	4065570	149.5	4065420.5	104.02542
4	B	56.3	31/05/2023	4584650	149.5	4584500.5	117.30757
4	C	56.3	31/05/2023	4386950	149.5	4386800.5	112.24885
5	A	28.1	31/05/2023	4197360	149.5	4197210.5	107.39764
5	B	28.1	31/05/2023	4705640	149.5	4705490.5	120.40344
5	C	28.1	31/05/2023	4701340	149.5	4701190.5	120.29341
6	A	14.1	31/05/2023	4348130	149.5	4347980.5	111.25552
6	B	14.1	31/05/2023	4660660	149.5	4660510.5	119.25250
6	C	14.1	31/05/2023	4760220	149.5	4760070.5	121.80003
7	A	7.0	31/05/2023	4091130	149.5	4090980.5	104.67944
7	B	7.0	31/05/2023	4682400	149.5	4682250.5	119.80878
7	C	7.0	31/05/2023	5026660	149.5	5026510.5	128.61766
8	A	3.5	31/05/2023	4120610	149.5	4120460.5	105.43377
8	B	3.5	31/05/2023	4454440	149.5	4454290.5	113.97577
8	C	3.5	31/05/2023	4683440	149.5	4683290.5	119.83539
9	A	1.8	31/05/2023	3784840	149.5	3784690.5	96.84214
9	B	1.8	31/05/2023	4776100	149.5	4775950.5	122.20636
9	C	1.8	31/05/2023	4568590	149.5	4568440.5	116.89662
10	A	0.9	31/05/2023	3903820	149.5	3903670.5	99.88658
10	B	0.9	31/05/2023	4570030	149.5	4569880.5	116.93347
10	C	0.9	31/05/2023	4325070	149.5	4324920.5	110.66547
11	A	0.4	31/05/2023	4161960	149.5	4161810.5	106.49183
11	B	0.4	31/05/2023	4830880	149.5	4830730.5	123.60807
11	C	0.4	31/05/2023	4517350	149.5	4517200.5	115.58550
12	A	0.2	31/05/2023	3862220	149.5	3862070.5	98.82213
12	B	0.2	31/05/2023	4679930	149.5	4679780.5	119.74558
12	C	0.2	31/05/2023	4241980	149.5	4241830.5	108.53937
9	H	Blank	31/05/2023	334			

10	H	Blank	31/05/2023	82			
11	H	Blank	31/05/2023	82			
12	H	Blank	31/05/2023	100			
1	A	450	07/06/2023	1473090	149.5	1472940.5	32.51571
1	B	450	07/06/2023	1346160	149.5	1346010.5	29.71368
1	C	450	07/06/2023	1249220	149.5	1249070.5	27.57369
2	A	225	07/06/2023	2914570	149.5	2914420.5	64.33691
2	B	225	07/06/2023	3386370	149.5	3386220.5	74.75207
2	C	225	07/06/2023	3185050	149.5	3184900.5	70.30786
3	A	112.5	07/06/2023	4627150	149.5	4627000.5	102.14275
3	B	112.5	07/06/2023	5276700	149.5	5276550.5	116.48180
3	C	112.5	07/06/2023	4998420	149.5	4998270.5	110.33867
4	A	56.25	07/06/2023	4980000	149.5	4979850.5	109.93204
4	B	56.25	07/06/2023	5579980	149.5	5579830.5	123.17682
4	C	56.25	07/06/2023	5494300	149.5	5494150.5	121.28540
5	A	28.1	07/06/2023	5757250	149.5	5757100.5	127.09012
5	B	28.1	07/06/2023	5784640	149.5	5784490.5	127.69477
5	C	28.1	07/06/2023	5404490	149.5	5404340.5	119.30281
6	A	14.1	07/06/2023	4975190	149.5	4975040.5	109.82586
6	B	14.1	07/06/2023	6113010	149.5	6112860.5	134.94365
6	C	14.1	07/06/2023	5281660	149.5	5281510.5	116.59130
7	A	7.0	07/06/2023	5209530	149.5	5209380.5	114.99900
7	B	7.0	07/06/2023	5783320	149.5	5783170.5	127.66563
7	C	7.0	07/06/2023	5487840	149.5	5487690.5	121.14280
8	A	3.5	07/06/2023	4808230	149.5	4808080.5	106.14015
8	B	3.5	07/06/2023	5460600	149.5	5460450.5	120.54146
8	C	3.5	07/06/2023	5454770	149.5	5454620.5	120.41276
9	A	1.8	07/06/2023	5175710	149.5	5175560.5	114.25241

9	B	1.8	07/06/2023	5765250	149.5	5765100.5	127.26672
9	C	1.8	07/06/2023	5047880	149.5	5047730.5	111.43052
10	A	0.9	07/06/2023	4751870	149.5	4751720.5	104.89599
10	B	0.9	07/06/2023	5769650	149.5	5769500.5	127.36386
10	C	0.9	07/06/2023	5303440	149.5	5303290.5	117.07210
11	A	0.4	07/06/2023	4716490	149.5	4716340.5	104.11496
11	B	0.4	07/06/2023	5530190	149.5	5530040.5	122.07769
11	C	0.4	07/06/2023	5110940	149.5	5110790.5	112.82259
12	A	0.2	07/06/2023	4690600	149.5	4690450.5	103.54343
12	B	0.2	07/06/2023	5401760	149.5	5401610.5	119.24255
12	C	0.2	07/06/2023	5638620	149.5	5638470.5	124.47132
9	H	Blank	07/06/2023	354			
10	H	Blank	07/06/2023	52			
11	H	Blank	07/06/2023	82			
12	H	Blank	07/06/2023	110			
1	A	450	14/06/2023	1764110	288.5	1763821.5	26.15357
1	B	450	14/06/2023	1575170	288.5	1574881.5	23.35201
1	C	450	14/06/2023	1648300	288.5	1648011.5	24.43637
2	A	225	14/06/2023	4514360	288.5	4514071.5	66.93370
2	B	225	14/06/2023	4520740	288.5	4520451.5	67.02830
2	C	225	14/06/2023	5471860	288.5	5471571.5	81.13131
3	A	112.5	14/06/2023	7423780	288.5	7423491.5	110.07396
3	B	112.5	14/06/2023	7560940	288.5	7560651.5	112.10774
3	C	112.5	14/06/2023	7926380	288.5	7926091.5	117.52641
4	A	56.25	14/06/2023	7610950	288.5	7610661.5	112.84928
4	B	56.25	14/06/2023	8301750	288.5	8301461.5	123.09232
4	C	56.25	14/06/2023	8031170	288.5	8030881.5	119.08021
5	A	28.1	14/06/2023	7869290	288.5	7869001.5	116.67989

5	B	28.1	14/06/2023	7705340	288.5	7705051.5	114.24887
5	C	28.1	14/06/2023	8684450	288.5	8684161.5	128.76691
6	A	14.1	14/06/2023	7777890	288.5	7777601.5	115.32463
6	B	14.1	14/06/2023	8006380	288.5	8006091.5	118.71263
6	C	14.1	14/06/2023	7855430	288.5	7855141.5	116.47438
7	A	7.0	14/06/2023	7847330	288.5	7847041.5	116.35427
7	B	7.0	14/06/2023	8198120	288.5	8197831.5	121.55571
7	C	7.0	14/06/2023	8144760	288.5	8144471.5	120.76450
8	A	3.5	14/06/2023	7998710	288.5	7998421.5	118.59890
8	B	3.5	14/06/2023	8210860	288.5	8210571.5	121.74462
8	C	3.5	14/06/2023	7982620	288.5	7982331.5	118.36032
9	A	1.8	14/06/2023	7793880	288.5	7793591.5	115.56173
9	B	1.8	14/06/2023	7817030	288.5	7816741.5	115.90499
9	C	1.8	14/06/2023	7980360	288.5	7980071.5	118.32681
10	A	0.9	14/06/2023	7602930	288.5	7602641.5	112.73036
10	B	0.9	14/06/2023	7480460	288.5	7480171.5	110.91440
10	C	0.9	14/06/2023	7902470	288.5	7902181.5	117.17188
11	A	0.4	14/06/2023	7590770	288.5	7590481.5	112.55005
11	B	0.4	14/06/2023	6965420	288.5	6965131.5	103.27750
11	C	0.4	14/06/2023	7726710	288.5	7726421.5	114.56574
12	A	0.2	14/06/2023	7460050	288.5	7459761.5	110.61177
12	B	0.2	14/06/2023	6855800	288.5	6855511.5	101.65208
12	C	0.2	14/06/2023	7247690	288.5	7247401.5	107.46294
9	H	Blank	14/06/2023	602			
10	H	Blank	14/06/2023	122			
11	H	Blank	14/06/2023	102			
12	H	Blank	14/06/2023	328			
1	G	**Reference Control**	31/05/2023	3653110	149.5	3652960.5	93.47145

2 G		31/05/2023	3886160	149.5	3886010.5	99.43470
3 G		31/05/2023	3933510	149.5	3933360.5	100.64629
4 G		31/05/2023	4160230	149.5	4160080.5	106.44757
1 G		07/06/2023	4306980	149.5	4306830.5	95.07487
2 G		07/06/2023	4541750	149.5	4541600.5	100.25751
3 G		07/06/2023	4486010	149.5	4485860.5	99.02703
4 G		07/06/2023	4785600	149.5	4785450.5	105.64059
1 G		14/06/2023	6675910	288.5	6675621.5	98.98470
2 G		14/06/2023	6429780	288.5	6429491.5	95.33514
3 G		14/06/2023	6864080	288.5	6863791.5	101.77485
4 G		14/06/2023	7007760	288.5	7007471.5	103.90531
5 G	**Medium Control**	31/05/2023	3969620	149.5	3969470.5	101.57026
6 G		31/05/2023	3748850	149.5	3748700.5	95.92123
7 G		31/05/2023	4383210	149.5	4383060.5	112.15315
8 G		31/05/2023	4000900	149.5	4000750.5	102.37065
5 G		07/06/2023	4788280	149.5	4788130.5	105.69975
6 G		07/06/2023	4753530	149.5	4753380.5	104.93263
7 G		07/06/2023	4889220	149.5	4889070.5	107.92804
8 G		07/06/2023	4675520	149.5	4675370.5	103.21053
5 G		14/06/2023	6741140	288.5	6740851.5	99.95192
6 G		14/06/2023	6401710	288.5	6401421.5	94.91892
7 G		14/06/2023	6698220	288.5	6697931.5	99.31551
8 G		14/06/2023	6490870	288.5	6490581.5	96.24097
9 G	**Positive Control**	31/05/2023	2942	149.5	2792.8	0.07146
10 G		31/05/2023	2018	149.5	1868.7	0.04782
11 G		31/05/2023	2126	149.5	1976.7	0.05058

12	G		31/05/2023	1754	149.5	1604.6	0.04106
9	G		07/06/2023	3637	149.5	3487.0	0.07698
10	G		07/06/2023	2456	149.5	2306.7	0.05092
11	G		07/06/2023	2342	149.5	2192.7	0.04841
12	G		07/06/2023	1758	149.5	1608.6	0.03551
9	G		14/06/2023	5363	288.5	5074.7	0.07525
10	G		14/06/2023	3691	288.5	3402.1	0.05044
11	G		14/06/2023	3629	288.5	3340.0	0.04953
12	G		14/06/2023	2802	288.5	2513.8	0.03727

KSL tested on MG63 in the presence of FBS

Well	Row	KSL [µg/mL]	Date	Sample (RLU)	Blank (RLU)	Sample-Blank	Cell activity (%)
1	A	450.0	11/11/2022	2169240	490.0	2168750.0	19.19011
1	B	450.0	11/11/2022	1595850	490.0	1595360.0	14.11649
1	C	450.0	11/11/2022	1547050	490.0	1546560.0	13.68469
2	A	225.0	11/11/2022	7491660	490.0	7491170.0	66.28537
2	B	225.0	11/11/2022	9533770	490.0	9533280.0	84.35491
2	C	225.0	11/11/2022	8671990	490.0	8671500.0	76.72948
3	A	112.5	11/11/2022	11037500	490.0	11037010.0	97.66062
3	B	112.5	11/11/2022	10768900	490.0	10768410.0	95.28392
3	C	112.5	11/11/2022	12122300	490.0	12121810.0	107.25944
4	A	56.3	11/11/2022	10937500	490.0	10937010.0	96.77577
4	B	56.3	11/11/2022	12932500	490.0	12932010.0	114.42847
4	C	56.3	11/11/2022	12290700	490.0	12290210.0	108.74952
5	A	28.1	11/11/2022	12313700	490.0	12313210.0	108.95303

5	B	28.1	11/11/2022	12606000	490.0	12605510.0	111.53944
5	C	28.1	11/11/2022	13154300	490.0	13153810.0	116.39106
6	A	14.1	11/11/2022	12708500	490.0	12708010.0	112.44641
6	B	14.1	11/11/2022	13662200	490.0	13661710.0	120.88519
6	C	14.1	11/11/2022	13361500	490.0	13361010.0	118.22446
7	A	7.0	11/11/2022	12882000	490.0	12881510.0	113.98162
7	B	7.0	11/11/2022	13787700	490.0	13787210.0	121.99567
7	C	7.0	11/11/2022	14360700	490.0	14360210.0	127.06585
8	A	3.5	11/11/2022	12624100	490.0	12623610.0	111.69960
8	B	3.5	11/11/2022	13721700	490.0	13721210.0	121.41168
8	C	3.5	11/11/2022	13567900	490.0	13567410.0	120.05078
9	A	1.8	11/11/2022	12324400	490.0	12323910.0	109.04771
9	B	1.8	11/11/2022	13304500	490.0	13304010.0	117.72010
9	C	1.8	11/11/2022	13025900	490.0	13025410.0	115.25491
10	A	0.9	11/11/2022	10953600	490.0	10953110.0	96.91823
10	B	0.9	11/11/2022	12146200	490.0	12145710.0	107.47092
10	C	0.9	11/11/2022	13145900	490.0	13145410.0	116.31673
11	A	0.4	11/11/2022	11366300	490.0	11365810.0	100.57000
11	B	0.4	11/11/2022	12505900	490.0	12505410.0	110.65371
11	C	0.4	11/11/2022	12756800	490.0	12756310.0	112.87379
12	A	0.2	11/11/2022	11300700	490.0	11300210.0	99.98954
12	B	0.2	11/11/2022	11601900	490.0	11601410.0	102.65469
12	C	0.2	11/11/2022	12528500	490.0	12528010.0	110.85368
9	H	Blank	11/11/2022	1040			
10	H	Blank	11/11/2022	268			
11	H	Blank	11/11/2022	232			
12	H	Blank	11/11/2022	420			
1	A	450	16/11/2022	1534950	533.0	1534417.0	14.28060

1	B	450	16/11/2022	1204300	533.0	1203767.0	11.20329
1	C	450	16/11/2022	1493780	533.0	1493247.0	13.89744
2	A	225	16/11/2022	8923560	533.0	8923027.0	83.04535
2	B	225	16/11/2022	8329130	533.0	8328597.0	77.51307
2	C	225	16/11/2022	9036600	533.0	9036067.0	84.09739
3	A	112.5	16/11/2022	12079600	533.0	12079067.0	112.41816
3	B	112.5	16/11/2022	12929600	533.0	12929067.0	120.32899
3	C	112.5	16/11/2022	12501100	533.0	12500567.0	116.34100
4	A	56.25	16/11/2022	12032800	533.0	12032267.0	111.98260
4	B	56.25	16/11/2022	12992300	533.0	12991767.0	120.91253
4	C	56.25	16/11/2022	12369900	533.0	12369367.0	115.11995
5	A	28.1	16/11/2022	12151900	533.0	12151367.0	113.09105
5	B	28.1	16/11/2022	13056300	533.0	13055767.0	121.50817
5	C	28.1	16/11/2022	13119200	533.0	13118667.0	122.09357
6	A	14.1	16/11/2022	11931800	533.0	11931267.0	111.04261
6	B	14.1	16/11/2022	12515200	533.0	12514667.0	116.47223
6	C	14.1	16/11/2022	12369400	533.0	12368867.0	115.11529
7	A	7.0	16/11/2022	12433700	533.0	12433167.0	115.71372
7	B	7.0	16/11/2022	12063300	533.0	12062767.0	112.26646
7	C	7.0	16/11/2022	12515900	533.0	12515367.0	116.47875
8	A	3.5	16/11/2022	12309400	533.0	12308867.0	114.55688
8	B	3.5	16/11/2022	12898800	533.0	12898267.0	120.04234
8	C	3.5	16/11/2022	12965500	533.0	12964967.0	120.66311
9	A	1.8	16/11/2022	12026000	533.0	12025467.0	111.91932
9	B	1.8	16/11/2022	11434700	533.0	11434167.0	106.41617
9	C	1.8	16/11/2022	12881400	533.0	12880867.0	119.88040
10	A	0.9	16/11/2022	11704200	533.0	11703667.0	108.92437
10	B	0.9	16/11/2022	13577300	533.0	13576767.0	126.35705

10	C	0.9	16/11/2022	12566200	533.0	12565667.0	116.94688
11	A	0.4	16/11/2022	11913900	533.0	11913367.0	110.87602
11	B	0.4	16/11/2022	12484700	533.0	12484167.0	116.18837
11	C	0.4	16/11/2022	13061900	533.0	13061367.0	121.56029
12	A	0.2	16/11/2022	11782700	533.0	11782167.0	109.65496
12	B	0.2	16/11/2022	12064600	533.0	12064067.0	112.27856
12	C	0.2	16/11/2022	12317300	533.0	12316767.0	114.63040
9	H	Blank	16/11/2022	1088			
10	H	Blank	16/11/2022	292			
11	H	Blank	16/11/2022	380			
12	H	Blank	16/11/2022	372			
1	A	450	18/11/2022	1894500	573.5	1893926.5	14.15602
1	B	450	18/11/2022	1421900	573.5	1421326.5	10.62360
1	C	450	18/11/2022	2286430	573.5	2285856.5	17.08547
2	A	225	18/11/2022	12860100	573.5	12859526.5	96.11760
2	B	225	18/11/2022	11848500	573.5	11847926.5	88.55646
2	C	225	18/11/2022	12504800	573.5	12504226.5	93.46193
3	A	112.5	18/11/2022	15975300	573.5	15974726.5	119.40193
3	B	112.5	18/11/2022	16405900	573.5	16405326.5	122.62042
3	C	112.5	18/11/2022	15246300	573.5	15245726.5	113.95307
4	A	56.25	18/11/2022	15737300	573.5	15736726.5	117.62302
4	B	56.25	18/11/2022	15523900	573.5	15523326.5	116.02798
4	C	56.25	18/11/2022	15802900	573.5	15802326.5	118.11334
5	A	28.1	18/11/2022	15756200	573.5	15755626.5	117.76428
5	B	28.1	18/11/2022	16194100	573.5	16193526.5	121.03734
5	C	28.1	18/11/2022	15023200	573.5	15022626.5	112.28553
6	A	14.1	18/11/2022	15636400	573.5	15635826.5	116.86885
6	B	14.1	18/11/2022	16536400	573.5	16535826.5	123.59583

6	C	14.1	18/11/2022	15468400	573.5	15467826.5	115.61314
7	A	7.0	18/11/2022	16373200	573.5	16372626.5	122.37601
7	B	7.0	18/11/2022	15700800	573.5	15700226.5	117.35020
7	C	7.0	18/11/2022	14980100	573.5	14979526.5	111.96338
8	A	3.5	18/11/2022	15492800	573.5	15492226.5	115.79552
8	B	3.5	18/11/2022	15720100	573.5	15719526.5	117.49446
8	C	3.5	18/11/2022	15305100	573.5	15304526.5	114.39257
9	A	1.8	18/11/2022	15016100	573.5	15015526.5	112.23246
9	B	1.8	18/11/2022	16360500	573.5	16359926.5	122.28108
9	C	1.8	18/11/2022	15481000	573.5	15480426.5	115.70732
10	A	0.9	18/11/2022	16608200	573.5	16607626.5	124.13250
10	B	0.9	18/11/2022	14676400	573.5	14675826.5	109.69340
10	C	0.9	18/11/2022	15306500	573.5	15305926.5	114.40303
11	A	0.4	18/11/2022	14430200	573.5	14429626.5	107.85319
11	B	0.4	18/11/2022	15276600	573.5	15276026.5	114.17955
11	C	0.4	18/11/2022	15278200	573.5	15277626.5	114.19151
12	A	0.2	18/11/2022	14256000	573.5	14255426.5	106.55115
12	B	0.2	18/11/2022	14421000	573.5	14420426.5	107.78443
12	C	0.2	18/11/2022	14570200	573.5	14569626.5	108.89961
9	H	Blank	18/11/2022	1148			
10	H	Blank	18/11/2022	268			
11	H	Blank	18/11/2022	328			
12	H	Blank	18/11/2022	550			
1	G	**Reference Control**	11/11/2022	9561430	490.0	9560940.0	84.59966
2	G		11/11/2022	11409400	490.0	11408910.0	100.95137
3	G		11/11/2022	11662700	490.0	11662210.0	103.19268
4	G		11/11/2022	12574000	490.0	12573510.0	111.25629
1	G		16/11/2022	9439390	533.0	9438857.0	87.84610

2 G		16/11/2022	10912300	533.0	10911767.0	101.55427
3 G		16/11/2022	11467300	533.0	11466767.0	106.71957
4 G		16/11/2022	11162200	533.0	11161667.0	103.88005
1 G		18/11/2022	12161500	573.5	12160926.5	90.89596
2 G		18/11/2022	11021100	573.5	11020526.5	82.37212
3 G		18/11/2022	14298700	573.5	14298126.5	106.87031
4 G		18/11/2022	16036800	573.5	16036226.5	119.86161
5 G	**Medium Control**	11/11/2022	12373000	490.0	12372510.0	109.47775
6 G		11/11/2022	12572800	490.0	12572310.0	111.24567
7 G		11/11/2022	12951300	490.0	12950810.0	114.59482
8 G		11/11/2022	13098700	490.0	13098210.0	115.89908
5 G		16/11/2022	10636600	533.0	10636067.0	98.98837
6 G		16/11/2022	9561570	533.0	9561037.0	88.98322
7 G		16/11/2022	9133550	533.0	9133017.0	84.99969
8 G		16/11/2022	12942000	533.0	12941467.0	120.44440
5 G		18/11/2022	16838000	573.5	16837426.5	125.85012
6 G		18/11/2022	13500300	573.5	13499726.5	100.90272
7 G		18/11/2022	15933100	573.5	15932526.5	119.08651
8 G		18/11/2022	13558200	573.5	13557626.5	101.33549
9 G	**Positive Control**	11/11/2022	7780	490.0	7290.5	0.06451
10 G		11/11/2022	4691	490.0	4200.9	0.03717
11 G		11/11/2022	4597	490.0	4106.9	0.03634
12 G		11/11/2022	3286	490.0	2796.4	0.02474
9 G		16/11/2022	7378	533.0	6845.2	0.06371
10 G		16/11/2022	4399	533.0	3865.8	0.03598
11 G		16/11/2022	5121	533.0	4588.1	0.04270

12	G		16/11/2022	3358	533.0	2825.4	0.02630
9	G		18/11/2022	8701	573.5	8127.6	0.06075
10	G		18/11/2022	5417	573.5	4843.7	0.03620
11	G		18/11/2022	5299	573.5	4725.6	0.03532
12	G		18/11/2022	3737	573.5	3163.1	0.02364

KSL tested on MG63 in the absence of FBS

Well	Row	KSL [μg/mL]	Date	Sample (RLU)	Blank (RLU)	Sample - Blank	Cell activity (%)
1	A	450.0	03/02/2023	806119	310.0	805809.0	8.91909
1	B	450.0	03/02/2023	872201	310.0	871891.0	9.65052
1	C	450.0	03/02/2023	896438	310.0	896128.0	9.91878
2	A	225.0	03/02/2023	6917200	310.0	6916890.0	76.55952
2	B	225.0	03/02/2023	6725420	310.0	6725110.0	74.43680
2	C	225.0	03/02/2023	7565100	310.0	7564790.0	83.73079
3	A	112.5	03/02/2023	9599060	310.0	9598750.0	106.24365
3	B	112.5	03/02/2023	8507060	310.0	8506750.0	94.15686
3	C	112.5	03/02/2023	10061700	310.0	10061390.0	111.36438
4	A	56.3	03/02/2023	9214910	310.0	9214600.0	101.99169
4	B	56.3	03/02/2023	8712690	310.0	8712380.0	96.43288
4	C	56.3	03/02/2023	9680720	310.0	9680410.0	107.14750
5	A	28.1	03/02/2023	8720900	310.0	8720590.0	96.52375
5	B	28.1	03/02/2023	9008480	310.0	9008170.0	99.70682
5	C	28.1	03/02/2023	9864400	310.0	9864090.0	109.18056
6	A	14.1	03/02/2023	8910650	310.0	8910340.0	98.62399
6	B	14.1	03/02/2023	8873080	310.0	8872770.0	98.20815
6	C	14.1	03/02/2023	9491580	310.0	9491270.0	105.05401

7	A	7.0	03/02/2023	9558570	310.0	9558260.0	105.79549
7	B	7.0	03/02/2023	8903130	310.0	8902820.0	98.54076
7	C	7.0	03/02/2023	9533390	310.0	9533080.0	105.51678
8	A	3.5	03/02/2023	9331990	310.0	9331680.0	103.28759
8	B	3.5	03/02/2023	9130390	310.0	9130080.0	101.05618
8	C	3.5	03/02/2023	9534010	310.0	9533700.0	105.52365
9	A	1.8	03/02/2023	8815630	310.0	8815320.0	97.57227
9	B	1.8	03/02/2023	8416210	310.0	8415900.0	93.15129
9	C	1.8	03/02/2023	9250940	310.0	9250630.0	102.39049
10	A	0.9	03/02/2023	9077970	310.0	9077660.0	100.47597
10	B	0.9	03/02/2023	8978290	310.0	8977980.0	99.37267
10	C	0.9	03/02/2023	9295540	310.0	9295230.0	102.88414
11	A	0.4	03/02/2023	8731550	310.0	8731240.0	96.64163
11	B	0.4	03/02/2023	8386490	310.0	8386180.0	92.82233
11	C	0.4	03/02/2023	9373840	310.0	9373530.0	103.75081
12	A	0.2	03/02/2023	9088950	310.0	9088640.0	100.59750
12	B	0.2	03/02/2023	8329880	310.0	8329570.0	92.19575
12	C	0.2	03/02/2023	9133420	310.0	9133110.0	101.08972
9	H	Blank	03/02/2023	726			
10	H	Blank	03/02/2023	122			
11	H	Blank	03/02/2023	124			
12	H	Blank	03/02/2023	268			
1	A	450	08/02/2023	402672	2661.3	400010.7	5.12972
1	B	450	08/02/2023	308893	2661.3	306231.7	3.92710
1	C	450	08/02/2023	431002	2661.3	428340.7	5.49302
2	A	225	08/02/2023	4806620	2661.3	4803958.7	61.60575
2	B	225	08/02/2023	6086110	2661.3	6083448.7	78.01387
2	C	225	08/02/2023	5544290	2661.3	5541628.7	71.06560

3	A	112.5	08/02/2023	8273440	2661.3	8270778.7	106.06410
3	B	112.5	08/02/2023	8914190	2661.3	8911528.7	114.28105
3	C	112.5	08/02/2023	8605250	2661.3	8602588.7	110.31921
4	A	56.25	08/02/2023	8345640	2661.3	8342978.7	106.98999
4	B	56.25	08/02/2023	8917930	2661.3	8915268.7	114.32901
4	C	56.25	08/02/2023	8831190	2661.3	8828528.7	113.21666
5	A	28.1	08/02/2023	8247250	2661.3	8244588.7	105.72824
5	B	28.1	08/02/2023	8786180	2661.3	8783518.7	112.63945
5	C	28.1	08/02/2023	9069690	2661.3	9067028.7	116.27517
6	A	14.1	08/02/2023	8822790	2661.3	8820128.7	113.10894
6	B	14.1	08/02/2023	9321520	2661.3	9318858.7	119.50463
6	C	14.1	08/02/2023	8093350	2661.3	8090688.7	103.75463
7	A	7.0	08/02/2023	8794260	2661.3	8791598.7	112.74307
7	B	7.0	08/02/2023	8527490	2661.3	8524828.7	109.32202
7	C	7.0	08/02/2023	8648940	2661.3	8646278.7	110.87949
8	A	3.5	08/02/2023	8363910	2661.3	8361248.7	107.22428
8	B	3.5	08/02/2023	8917760	2661.3	8915098.7	114.32683
8	C	3.5	08/02/2023	8112430	2661.3	8109768.7	103.99931
9	A	1.8	08/02/2023	8100710	2661.3	8098048.7	103.84901
9	B	1.8	08/02/2023	8225830	2661.3	8223168.7	105.45355
9	C	1.8	08/02/2023	7979080	2661.3	7976418.7	102.28924
10	A	0.9	08/02/2023	9012950	2661.3	9010288.7	115.54754
10	B	0.9	08/02/2023	8189750	2661.3	8187088.7	104.99086
10	C	0.9	08/02/2023	7596370	2661.3	7593708.7	97.38138
11	A	0.4	08/02/2023	8509220	2661.3	8506558.7	109.08773
11	B	0.4	08/02/2023	8591550	2661.3	8588888.7	110.14352
11	C	0.4	08/02/2023	8119510	2661.3	8116848.7	104.09010
12	A	0.2	08/02/2023	8415400	2661.3	8412738.7	107.88458

12	B	0.2	08/02/2023	8219710	2661.3	8217048.7	105.37506
12	C	0.2	08/02/2023	7831950	2661.3	7829288.7	100.40245
1	H	Blank	08/02/2023	1976			
2	H	Blank	08/02/2023	3092			
3	H	Blank	08/02/2023	2938			
4	H	Blank	08/02/2023	2638			
1	A	450.0	10/02/2023	261430	357.5	261072.5	3.66065
1	B	450.0	10/02/2023	552850	357.5	552492.5	7.74683
1	C	450.0	10/02/2023	364656	357.5	364298.5	5.10805
2	A	225.0	10/02/2023	6827300	357.5	6826942.5	95.72464
2	B	225.0	10/02/2023	6296120	357.5	6295762.5	88.27665
2	C	225.0	10/02/2023	5355900	357.5	5355542.5	75.09326
3	A	112.5	10/02/2023	8699790	357.5	8699432.5	121.97994
3	B	112.5	10/02/2023	9126800	357.5	9126442.5	127.96730
3	C	112.5	10/02/2023	9662200	357.5	9661842.5	135.47447
4	A	56.3	10/02/2023	8529280	357.5	8528922.5	119.58912
4	B	56.3	10/02/2023	9295260	357.5	9294902.5	130.32938
4	C	56.3	10/02/2023	8781970	357.5	8781612.5	123.13224
5	A	28.1	10/02/2023	8474090	357.5	8473732.5	118.81527
5	B	28.1	10/02/2023	9789410	357.5	9789052.5	137.25815
5	C	28.1	10/02/2023	8853920	357.5	8853562.5	124.14109
6	A	14.1	10/02/2023	8353540	357.5	8353182.5	117.12496
6	B	14.1	10/02/2023	9242290	357.5	9241932.5	129.58666
6	C	14.1	10/02/2023	8598550	357.5	8598192.5	120.56039
7	A	7.0	10/02/2023	8732490	357.5	8732132.5	122.43845
7	B	7.0	10/02/2023	9474520	357.5	9474162.5	132.84289
7	C	7.0	10/02/2023	8748900	357.5	8748542.5	122.66854
8	A	3.5	10/02/2023	8322960	357.5	8322602.5	116.69618

8	B	3.5	10/02/2023	9085110	357.5	9084752.5	127.38274
8	C	3.5	10/02/2023	8688410	357.5	8688052.5	121.82037
9	A	1.8	10/02/2023	8031210	357.5	8030852.5	112.60538
9	B	1.8	10/02/2023	8735600	357.5	8735242.5	122.48205
9	C	1.8	10/02/2023	9272110	357.5	9271752.5	130.00478
10	A	0.9	10/02/2023	8485180	357.5	8484822.5	118.97077
10	B	0.9	10/02/2023	8772420	357.5	8772062.5	122.99833
10	C	0.9	10/02/2023	8886120	357.5	8885762.5	124.59258
11	A	0.4	10/02/2023	8405530	357.5	8405172.5	117.85395
11	B	0.4	10/02/2023	8922280	357.5	8921922.5	125.09961
11	C	0.4	10/02/2023	8870060	357.5	8869702.5	124.36740
12	A	0.2	10/02/2023	8612850	357.5	8612492.5	120.76090
12	B	0.2	10/02/2023	8639820	357.5	8639462.5	121.13907
12	C	0.2	10/02/2023	8937040	357.5	8936682.5	125.30656
9	H	Blank	10/02/2023	1044			
10	H	Blank	10/02/2023	118			
11	H	Blank	10/02/2023	84			
12	H	Blank	10/02/2023	184			
1	A	450.0	19/04/2024	1044310	254.5	1044055.5	15.86696
1	B	450.0	19/04/2024	1358820	254.5	1358565.5	20.64670
1	C	450.0	19/04/2024	1352690	254.5	1352435.5	20.55354
2	A	225.0	19/04/2024	7005920	254.5	7005665.5	106.46810
2	B	225.0	19/04/2024	7980630	254.5	7980375.5	121.28119
2	C	225.0	19/04/2024	7370990	254.5	7370735.5	112.01623
3	A	112.5	19/04/2024	7360010	254.5	7359755.5	111.84936
3	B	112.5	19/04/2024	7921120	254.5	7920865.5	120.37679
3	C	112.5	19/04/2024	7892460	254.5	7892205.5	119.94123
4	A	56.3	19/04/2024	7132300	254.5	7132045.5	108.38875

4	B	56.3	19/04/2024	8604730	254.5	8604475.5	130.76590
4	C	56.3	19/04/2024	8116090	254.5	8115835.5	123.33983
5	A	28.1	19/04/2024	7230150	254.5	7229895.5	109.87582
5	B	28.1	19/04/2024	8401300	254.5	8401045.5	127.67429
5	C	28.1	19/04/2024	7822300	254.5	7822045.5	118.87498
6	A	14.1	19/04/2024	6854970	254.5	6854715.5	104.17405
6	B	14.1	19/04/2024	7634840	254.5	7634585.5	116.02607
6	C	14.1	19/04/2024	7912590	254.5	7912335.5	120.24715
7	A	7.0	19/04/2024	6779740	254.5	6779485.5	103.03075
7	B	7.0	19/04/2024	7754910	254.5	7754655.5	117.85082
7	C	7.0	19/04/2024	7663540	254.5	7663285.5	116.46223
8	A	3.5	19/04/2024	7227560	254.5	7227305.5	109.83646
8	B	3.5	19/04/2024	7412640	254.5	7412385.5	112.64920
8	C	3.5	19/04/2024	7748710	254.5	7748455.5	117.75660
9	A	1.8	19/04/2024	6622160	254.5	6621905.5	100.63594
9	B	1.8	19/04/2024	7760870	254.5	7760615.5	117.94140
9	C	1.8	19/04/2024	7601430	254.5	7601175.5	115.51832
10	A	0.9	19/04/2024	7705660	254.5	7705405.5	117.10235
10	B	0.9	19/04/2024	7700910	254.5	7700655.5	117.03016
10	C	0.9	19/04/2024	7481400	254.5	7481145.5	113.69418
11	A	0.4	19/04/2024	6611560	254.5	6611305.5	100.47484
11	B	0.4	19/04/2024	7650930	254.5	7650675.5	116.27060
11	C	0.4	19/04/2024	7520170	254.5	7519915.5	114.28338
12	A	0.2	19/04/2024	7479620	254.5	7479365.5	113.66712
12	B	0.2	19/04/2024	7982110	254.5	7981855.5	121.30368
12	C	0.2	19/04/2024	7869410	254.5	7869155.5	119.59093
9	H	Blank	19/04/2024	478			
10	H	Blank	19/04/2024	148			

11	H	Blank	19/04/2024	130			
12	H	Blank	19/04/2024	262			
1	G	**Reference Control**	03/02/2023	9270520	310.0	9270210.0	102.60721
2	G		03/02/2023	8686740	310.0	8686430.0	96.14565
3	G		03/02/2023	9199430	310.0	9199120.0	101.82035
4	G		03/02/2023	8983180	310.0	8982870.0	99.42679
1	G		08/02/2023	7886050	2661.3	7883388.7	101.09622
2	G		08/02/2023	7812290	2661.3	7809628.7	100.15033
3	G		08/02/2023	8036210	2661.3	8033548.7	103.02187
4	G		08/02/2023	7467720	2661.3	7465058.7	95.73158
1	G		10/02/2023	7159270	357.5	7158912.5	100.37939
2	G		10/02/2023	6586440	357.5	6586082.5	92.34740
3	G		10/02/2023	7149200	357.5	7148842.5	100.23819
4	G		10/02/2023	7633940	357.5	7633582.5	107.03502
1	G		19/04/2024	5769240	254.5	5768985.5	87.67375
2	G		19/04/2024	6218160	254.5	6217905.5	94.49618
3	G		19/04/2024	6974500	254.5	6974245.5	105.99060
4	G		19/04/2024	7359360	254.5	7359105.5	111.83948
5	G	**Medium Control**	03/02/2023	9163880	310.0	9163570.0	101.42687
6	G		03/02/2023	9113030	310.0	9112720.0	100.86403
7	G		03/02/2023	8924720	310.0	8924410.0	98.77973
8	G		03/02/2023	9271100	310.0	9270790.0	102.61363
5	G		08/02/2023	7710800	2661.3	7708138.7	98.84883
6	G		08/02/2023	7177000	2661.3	7174338.7	92.00340
7	G		08/02/2023	7640920	2661.3	7638258.7	97.95269
8	G		08/02/2023	7407250	2661.3	7404588.7	94.95611
5	G		10/02/2023	7278620	357.5	7278262.5	102.05287

6 G		10/02/2023	7280820	357.5	7280462.5	102.08371
7 G		10/02/2023	7649660	357.5	7649302.5	107.25544
8 G		10/02/2023	7060490	357.5	7060132.5	98.99434
5 G		19/04/2024	7213680	254.5	7213425.5	109.62552
6 G		19/04/2024	6478530	254.5	6478275.5	98.45313
7 G		19/04/2024	6635090	254.5	6634835.5	100.83244
8 G		19/04/2024	5823810	254.5	5823555.5	88.50307
9 G	**Positive Control**	03/02/2023	24098	310.0	23787.8	0.26329
10 G		03/02/2023	13920	310.0	13609.9	0.15064
11 G		03/02/2023	11457	310.0	11147.4	0.12338
12 G		03/02/2023	8191	310.0	7880.7	0.08723
9 G		08/02/2023	15446	2661.3	12784.5	0.16395
10 G		08/02/2023	10861	2661.3	8199.5	0.10515
11 G		08/02/2023	17599	2661.3	14937.4	0.19156
12 G		08/02/2023	15266	2661.3	12604.2	0.16164
9 G		10/02/2023	12949	357.5	12591.4	0.17655
10 G		10/02/2023	26320	357.5	25962.9	0.36404
11 G		10/02/2023	17344	357.5	16986.8	0.23818
12 G		10/02/2023	12428	357.5	12070.8	0.16925
9 G		19/04/2024	18819	254.5	18564.0	0.28213
10 G		19/04/2024	25549	254.5	25294.2	0.38441
11 G		19/04/2024	21092	254.5	20837.7	0.31668
12 G		19/04/2024	21737	254.5	21482.9	0.32648

KSL tested on L929 in the presence of FBS

Well	Row	KSL [µg/mL]	Date	Sample (RLU)	Blank (RLU)	Sample-Blank	Cell activity (%)
1	A	450.0	03/02/2023	12015100	498.0	12014602.0	98.30547
1	B	450.0	03/02/2023	12350700	498.0	12350202.0	101.05141
1	C	450.0	03/02/2023	13128400	498.0	13127902.0	107.41468
2	A	225.0	03/02/2023	13480400	498.0	13479902.0	110.29480
2	B	225.0	03/02/2023	13559800	498.0	13559302.0	110.94447
2	C	225.0	03/02/2023	14224600	498.0	14224102.0	116.38397
3	A	112.5	03/02/2023	13064800	498.0	13064302.0	106.89429
3	B	112.5	03/02/2023	13648800	498.0	13648302.0	111.67268
3	C	112.5	03/02/2023	14093300	498.0	14092802.0	115.30965
4	A	56.3	03/02/2023	13400900	498.0	13400402.0	109.64432
4	B	56.3	03/02/2023	13296600	498.0	13296102.0	108.79092
4	C	56.3	03/02/2023	14138400	498.0	14137902.0	115.67867
5	A	28.1	03/02/2023	12732900	498.0	12732402.0	104.17863
5	B	28.1	03/02/2023	13823300	498.0	13822802.0	113.10047
5	C	28.1	03/02/2023	12832100	498.0	12831602.0	104.99030
6	A	14.1	03/02/2023	13152300	498.0	13151802.0	107.61023
6	B	14.1	03/02/2023	14060200	498.0	14059702.0	115.03882
6	C	14.1	03/02/2023	13442000	498.0	13441502.0	109.98061
7	A	7.0	03/02/2023	13024300	498.0	13023802.0	106.56292
7	B	7.0	03/02/2023	13776500	498.0	13776002.0	112.71754
7	C	7.0	03/02/2023	12904000	498.0	12903502.0	105.57860
8	A	3.5	03/02/2023	12917200	498.0	12916702.0	105.68661
8	B	3.5	03/02/2023	13609500	498.0	13609002.0	111.35112
8	C	3.5	03/02/2023	13285200	498.0	13284702.0	108.69764

9	A	1.8	03/02/2023	13448500	498.0	13448002.0	110.03379
9	B	1.8	03/02/2023	13613900	498.0	13613402.0	111.38712
9	C	1.8	03/02/2023	13331200	498.0	13330702.0	109.07402
10	A	0.9	03/02/2023	12814600	498.0	12814102.0	104.84712
10	B	0.9	03/02/2023	13453000	498.0	13452502.0	110.07061
10	C	0.9	03/02/2023	13136000	498.0	13135502.0	107.47686
11	A	0.4	03/02/2023	12821500	498.0	12821002.0	104.90357
11	B	0.4	03/02/2023	13236000	498.0	13235502.0	108.29508
11	C	0.4	03/02/2023	14063400	498.0	14062902.0	115.06500
12	A	0.2	03/02/2023	12978800	498.0	12978302.0	106.19063
12	B	0.2	03/02/2023	12872800	498.0	12872302.0	105.32332
12	C	0.2	03/02/2023	13057700	498.0	13057202.0	106.83620
9	H	Blank	03/02/2023	1040			
10	H	Blank	03/02/2023	270			
11	H	Blank	03/02/2023	340			
12	H	Blank	03/02/2023	342			
1	A	450	08/02/2023	13992500	538.0	13991962.0	106.89261
1	B	450	08/02/2023	14311700	538.0	14311162.0	109.33117
1	C	450	08/02/2023	14452000	538.0	14451462.0	110.40300
2	A	225	08/02/2023	14732800	538.0	14732262.0	112.54819
2	B	225	08/02/2023	16185300	538.0	16184762.0	123.64467
2	C	225	08/02/2023	15966300	538.0	15965762.0	121.97160
3	A	112.5	08/02/2023	14836800	538.0	14836262.0	113.34271
3	B	112.5	08/02/2023	15532700	538.0	15532162.0	118.65908
3	C	112.5	08/02/2023	15035600	538.0	15035062.0	114.86145
4	A	56.25	08/02/2023	15064500	538.0	15063962.0	115.08224
4	B	56.25	08/02/2023	15634400	538.0	15633862.0	119.43603
4	C	56.25	08/02/2023	15753200	538.0	15752662.0	120.34361

5	A	28.1	08/02/2023	14781800	538.0	14781262.0	112.92253
5	B	28.1	08/02/2023	16085700	538.0	16085162.0	122.88377
5	C	28.1	08/02/2023	15238800	538.0	15238262.0	116.41381
6	A	14.1	08/02/2023	15410100	538.0	15409562.0	117.72247
6	B	14.1	08/02/2023	14663600	538.0	14663062.0	112.01953
6	C	14.1	08/02/2023	15236200	538.0	15235662.0	116.39395
7	A	7.0	08/02/2023	15528200	538.0	15527662.0	118.62471
7	B	7.0	08/02/2023	15642800	538.0	15642262.0	119.50020
7	C	7.0	08/02/2023	15897800	538.0	15897262.0	121.44829
8	A	3.5	08/02/2023	15107300	538.0	15106762.0	115.40921
8	B	3.5	08/02/2023	15571800	538.0	15571262.0	118.95779
8	C	3.5	08/02/2023	15771200	538.0	15770662.0	120.48112
9	A	1.8	08/02/2023	15556300	538.0	15555762.0	118.83938
9	B	1.8	08/02/2023	14706800	538.0	14706262.0	112.34956
9	C	1.8	08/02/2023	15833900	538.0	15833362.0	120.96012
10	A	0.9	08/02/2023	15003100	538.0	15002562.0	114.61317
10	B	0.9	08/02/2023	15336400	538.0	15335862.0	117.15944
10	C	0.9	08/02/2023	16117000	538.0	16116462.0	123.12289
11	A	0.4	08/02/2023	14788400	538.0	14787862.0	112.97295
11	B	0.4	08/02/2023	13722800	538.0	13722262.0	104.83222
11	C	0.4	08/02/2023	15602600	538.0	15602062.0	119.19309
12	A	0.2	08/02/2023	13777900	538.0	13777362.0	105.25316
12	B	0.2	08/02/2023	14780600	538.0	14780062.0	112.91336
12	C	0.2	08/02/2023	14916500	538.0	14915962.0	113.95158
9	H	Blank	08/02/2023	1152			
10	H	Blank	08/02/2023	296			
11	H	Blank	08/02/2023	296			
12	H	Blank	08/02/2023	408			

1	A	450	10/02/2023	12430000	559.0	12429441.0	90.34688
1	B	450	10/02/2023	12833900	559.0	12833341.0	93.28274
1	C	450	10/02/2023	13208600	559.0	13208041.0	96.00635
2	A	225	10/02/2023	13510300	559.0	13509741.0	98.19934
2	B	225	10/02/2023	14819600	559.0	14819041.0	107.71636
2	C	225	10/02/2023	14277400	559.0	14276841.0	103.77522
3	A	112.5	10/02/2023	13916400	559.0	13915841.0	101.15119
3	B	112.5	10/02/2023	14252600	559.0	14252041.0	103.59496
3	C	112.5	10/02/2023	14601100	559.0	14600541.0	106.12813
4	A	56.25	10/02/2023	12657900	559.0	12657341.0	92.00343
4	B	56.25	10/02/2023	14607900	559.0	14607341.0	106.17755
4	C	56.25	10/02/2023	14404800	559.0	14404241.0	104.70127
5	A	28.1	10/02/2023	13459100	559.0	13458541.0	97.82718
5	B	28.1	10/02/2023	13984300	559.0	13983741.0	101.64474
5	C	28.1	10/02/2023	14527100	559.0	14526541.0	105.59024
6	A	14.1	10/02/2023	13192800	559.0	13192241.0	95.89150
6	B	14.1	10/02/2023	13968700	559.0	13968141.0	101.53135
6	C	14.1	10/02/2023	14346200	559.0	14345641.0	104.27531
7	A	7.0	10/02/2023	13607100	559.0	13606541.0	98.90296
7	B	7.0	10/02/2023	14643700	559.0	14643141.0	106.43778
7	C	7.0	10/02/2023	14740800	559.0	14740241.0	107.14358
8	A	3.5	10/02/2023	14716600	559.0	14716041.0	106.96767
8	B	3.5	10/02/2023	13828200	559.0	13827641.0	100.51009
8	C	3.5	10/02/2023	15074100	559.0	15073541.0	109.56626
9	A	1.8	10/02/2023	14077600	559.0	14077041.0	102.32292
9	B	1.8	10/02/2023	13880200	559.0	13879641.0	100.88806
9	C	1.8	10/02/2023	15346800	559.0	15346241.0	111.54846
10	A	0.9	10/02/2023	13276100	559.0	13275541.0	96.49699

10	B	0.9	10/02/2023	14481100	559.0	14480541.0	105.25587
10	C	0.9	10/02/2023	15275900	559.0	15275341.0	111.03310
11	A	0.4	10/02/2023	13678000	559.0	13677441.0	99.41832
11	B	0.4	10/02/2023	14243300	559.0	14242741.0	103.52736
11	C	0.4	10/02/2023	15239100	559.0	15238541.0	110.76561
12	A	0.2	10/02/2023	12475600	559.0	12475041.0	90.67833
12	B	0.2	10/02/2023	14551100	559.0	14550541.0	105.76469
12	C	0.2	10/02/2023	14499400	559.0	14498841.0	105.38889
9	H	Blank	10/02/2023	1182			
10	H	Blank	10/02/2023	264			
11	H	Blank	10/02/2023	286			
12	H	Blank	10/02/2023	504			
1	G	**Reference Control**	03/02/2023	11310400	498.0	11309902.0	92.53950
2	G		03/02/2023	11959100	498.0	11958602.0	97.84727
3	G		03/02/2023	12523500	498.0	12523002.0	102.46529
4	G		03/02/2023	13095800	498.0	13095302.0	107.14794
1	G		08/02/2023	11525200	538.0	11524662.0	88.04350
2	G		08/02/2023	13004100	538.0	13003562.0	99.34166
3	G		08/02/2023	14083200	538.0	14082662.0	107.58552
4	G		08/02/2023	13748600	538.0	13748062.0	105.02932
1	G		10/02/2023	13761200	559.0	13760641.0	100.02308
2	G		10/02/2023	13552800	559.0	13552241.0	98.50826
3	G		10/02/2023	13844700	559.0	13844141.0	100.63002
4	G		10/02/2023	13873400	559.0	13872841.0	100.83864
5	G	**Medium Control**	03/02/2023	12525400	498.0	12524902.0	102.48083
6	G		03/02/2023	12654100	498.0	12653602.0	103.53388
7	G		03/02/2023	12642000	498.0	12641502.0	103.43487

8	G		03/02/2023	12273500	498.0	12273002.0	100.41975
5	G		08/02/2023	13769700	538.0	13769162.0	105.19052
6	G		08/02/2023	13893500	538.0	13892962.0	106.13630
7	G		08/02/2023	13704400	538.0	13703862.0	104.69165
8	G		08/02/2023	14693900	538.0	14693362.0	112.25101
5	G		10/02/2023	13681200	559.0	13680641.0	99.44158
6	G		10/02/2023	14123700	559.0	14123141.0	102.65801
7	G		10/02/2023	14947500	559.0	14946941.0	108.64603
8	G		10/02/2023	14410800	559.0	14410241.0	104.74488
9	G	**Positive Control**	03/02/2023	7977	498.0	7478.6	0.06119
10	G		03/02/2023	5337	498.0	4839.2	0.03959
11	G		03/02/2023	5737	498.0	5239.3	0.04287
12	G		03/02/2023	4309	498.0	3810.7	0.03118
9	G		08/02/2023	8833	538.0	8295.2	0.06337
10	G		08/02/2023	5761	538.0	5223.3	0.03990
11	G		08/02/2023	5987	538.0	5449.5	0.04163
12	G		08/02/2023	4553	538.0	4014.8	0.03067
9	G		10/02/2023	9612	559.0	9052.8	0.06580
10	G		10/02/2023	6236	559.0	5676.6	0.04126
11	G		10/02/2023	6690	559.0	6130.8	0.04456
12	G		10/02/2023	4781	559.0	4221.9	0.03069

KSL tested on L929 in the absence of FBS

Well	Row	KSL [µg/mL]	Date	Sample (RLU)	Blank (RLU)	Sample-Blank	Cell activity (%)
1	A	450.0	15/02/2023	1743530	260.5	1743269.5	24.13826

1	B	450.0	15/02/2023	2050020	260.5	2049759.5	28.38208
1	C	450.0	15/02/2023	2474890	260.5	2474629.5	34.26506
2	A	225.0	15/02/2023	8246050	260.5	8245789.5	114.17567
2	B	225.0	15/02/2023	8478650	260.5	8478389.5	117.39638
2	C	225.0	15/02/2023	9879160	260.5	9878899.5	136.78860
3	A	112.5	15/02/2023	9699830	260.5	9699569.5	134.30550
3	B	112.5	15/02/2023	10247900	260.5	10247639.5	141.89438
3	C	112.5	15/02/2023	10001900	260.5	10001639.5	138.48813
4	A	56.3	15/02/2023	9320530	260.5	9320269.5	129.05351
4	B	56.3	15/02/2023	9752220	260.5	9751959.5	135.03092
4	C	56.3	15/02/2023	9907910	260.5	9907649.5	137.18669
5	A	28.1	15/02/2023	8666490	260.5	8666229.5	119.99732
5	B	28.1	15/02/2023	10066500	260.5	10066239.5	139.38261
5	C	28.1	15/02/2023	9383340	260.5	9383079.5	129.92321
6	A	14.1	15/02/2023	10251500	260.5	10251239.5	141.94422
6	B	14.1	15/02/2023	10676100	260.5	10675839.5	147.82347
6	C	14.1	15/02/2023	10285300	260.5	10285039.5	142.41224
7	A	7.0	15/02/2023	9918780	260.5	9918519.5	137.33720
7	B	7.0	15/02/2023	10229400	260.5	10229139.5	141.63821
7	C	7.0	15/02/2023	10868800	260.5	10868539.5	150.49169
8	A	3.5	15/02/2023	9946000	260.5	9945739.5	137.71410
8	B	3.5	15/02/2023	10653100	260.5	10652839.5	147.50499
8	C	3.5	15/02/2023	9921000	260.5	9920739.5	137.36794
9	A	1.8	15/02/2023	9717050	260.5	9716789.5	134.54394
9	B	1.8	15/02/2023	10644500	260.5	10644239.5	147.38591
9	C	1.8	15/02/2023	10441400	260.5	10441139.5	144.57368
10	A	0.9	15/02/2023	9670770	260.5	9670509.5	133.90312
10	B	0.9	15/02/2023	10784100	260.5	10783839.5	149.31889

10	C	0.9	15/02/2023	10721300	260.5	10721039.5	148.44933
11	A	0.4	15/02/2023	9849620	260.5	9849359.5	136.37958
11	B	0.4	15/02/2023	10316200	260.5	10315939.5	142.84009
11	C	0.4	15/02/2023	9834080	260.5	9833819.5	136.16440
12	A	0.2	15/02/2023	9413040	260.5	9412779.5	130.33445
12	B	0.2	15/02/2023	10935100	260.5	10934839.5	151.40972
12	C	0.2	15/02/2023	10612300	260.5	10612039.5	146.94006
9	H	Blank	15/02/2023	486			
10	H	Blank	15/02/2023	114			
11	H	Blank	15/02/2023	110			
12	H	Blank	15/02/2023	332			
1	A	450	17/02/2023	1144520	231.5	1144288.5	14.10391
1	B	450	17/02/2023	2269590	231.5	2269358.5	27.97094
1	C	450	17/02/2023	2244320	231.5	2244088.5	27.65947
2	A	225	17/02/2023	5196650	231.5	5196418.5	64.04836
2	B	225	17/02/2023	8275510	231.5	8275278.5	101.99679
2	C	225	17/02/2023	8509970	231.5	8509738.5	104.88662
3	A	112.5	17/02/2023	6191750	231.5	6191518.5	76.31344
3	B	112.5	17/02/2023	8403260	231.5	8403028.5	103.57137
3	C	112.5	17/02/2023	8532860	231.5	8532628.5	105.16875
4	A	56.25	17/02/2023	8349010	231.5	8348778.5	102.90271
4	B	56.25	17/02/2023	9205970	231.5	9205738.5	113.46516
4	C	56.25	17/02/2023	9116850	231.5	9116618.5	112.36671
5	A	28.1	17/02/2023	6050300	231.5	6050068.5	74.57001
5	B	28.1	17/02/2023	9062520	231.5	9062288.5	111.69707
5	C	28.1	17/02/2023	9225160	231.5	9224928.5	113.70168
6	A	14.1	17/02/2023	6863580	231.5	6863348.5	84.59407
6	B	14.1	17/02/2023	8667000	231.5	8666768.5	106.82209

6	C	14.1	17/02/2023	9332630	231.5	9332398.5	115.02630
7	A	7.0	17/02/2023	7537750	231.5	7537518.5	92.90354
7	B	7.0	17/02/2023	8890970	231.5	8890738.5	109.58263
7	C	7.0	17/02/2023	8538760	231.5	8538528.5	105.24147
8	A	3.5	17/02/2023	7795520	231.5	7795288.5	96.08068
8	B	3.5	17/02/2023	9089390	231.5	9089158.5	112.02825
8	C	3.5	17/02/2023	8826960	231.5	8826728.5	108.79368
9	A	1.8	17/02/2023	8313600	231.5	8313368.5	102.46627
9	B	1.8	17/02/2023	9107240	231.5	9107008.5	112.24826
9	C	1.8	17/02/2023	8478740	231.5	8478508.5	104.50170
10	A	0.9	17/02/2023	8720360	231.5	8720128.5	107.47978
10	B	0.9	17/02/2023	9310540	231.5	9310308.5	114.75403
10	C	0.9	17/02/2023	8802190	231.5	8801958.5	108.48837
11	A	0.4	17/02/2023	8514730	231.5	8514498.5	104.94529
11	B	0.4	17/02/2023	9026460	231.5	9026228.5	111.25261
11	C	0.4	17/02/2023	8387450	231.5	8387218.5	103.37650
12	A	0.2	17/02/2023	8910490	231.5	8910258.5	109.82322
12	B	0.2	17/02/2023	11305900	231.5	11305668.5	139.34780
12	C	0.2	17/02/2023	8177290	231.5	8177058.5	100.78618
9	H	Blank	17/02/2023	472			
10	H	Blank	17/02/2023	120			
11	H	Blank	17/02/2023	86			
12	H	Blank	17/02/2023	248			
1	A	450	22/02/2023	1872730	333.0	1872397.0	22.28831
1	B	450	22/02/2023	3227360	333.0	3227027.0	38.41331
1	C	450	22/02/2023	2918090	333.0	2917757.0	34.73188
2	A	225	22/02/2023	8918210	333.0	8917877.0	106.15504
2	B	225	22/02/2023	9271680	333.0	9271347.0	110.36261

2	C	225	22/02/2023	9518380	333.0	9518047.0	113.29923
3	A	112.5	22/02/2023	9280410	333.0	9280077.0	110.46653
3	B	112.5	22/02/2023	10000600	333.0	10000267.0	119.03940
3	C	112.5	22/02/2023	10097400	333.0	10097067.0	120.19167
4	A	56.25	22/02/2023	8835790	333.0	8835457.0	105.17394
4	B	56.25	22/02/2023	9644990	333.0	9644657.0	114.80635
4	C	56.25	22/02/2023	9804000	333.0	9803667.0	116.69914
5	A	28.1	22/02/2023	8477490	333.0	8477157.0	100.90887
5	B	28.1	22/02/2023	9624830	333.0	9624497.0	114.56637
5	C	28.1	22/02/2023	9741540	333.0	9741207.0	115.95564
6	A	14.1	22/02/2023	9010040	333.0	9009707.0	107.24815
6	B	14.1	22/02/2023	9634590	333.0	9634257.0	114.68255
6	C	14.1	22/02/2023	9638800	333.0	9638467.0	114.73267
7	A	7.0	22/02/2023	9229930	333.0	9229597.0	109.86563
7	B	7.0	22/02/2023	9065570	333.0	9065237.0	107.90915
7	C	7.0	22/02/2023	9527520	333.0	9527187.0	113.40803
8	A	3.5	22/02/2023	8598710	333.0	8598377.0	102.35183
8	B	3.5	22/02/2023	10387300	333.0	10386967.0	123.64253
8	C	3.5	22/02/2023	9330100	333.0	9329767.0	111.05802
9	A	1.8	22/02/2023	8999810	333.0	8999477.0	107.12637
9	B	1.8	22/02/2023	9825510	333.0	9825177.0	116.95519
9	C	1.8	22/02/2023	9775700	333.0	9775367.0	116.36227
10	A	0.9	22/02/2023	9300250	333.0	9299917.0	110.70270
10	B	0.9	22/02/2023	9716200	333.0	9715867.0	115.65401
10	C	0.9	22/02/2023	10154400	333.0	10154067.0	120.87017
11	A	0.4	22/02/2023	9455270	333.0	9454937.0	112.54799
11	B	0.4	22/02/2023	10127800	333.0	10127467.0	120.55354
11	C	0.4	22/02/2023	9701170	333.0	9700837.0	115.47510

12	A	0.2	22/02/2023	9467900	333.0	9467567.0	112.69834
12	B	0.2	22/02/2023	9965640	333.0	9965307.0	118.62325
12	C	0.2	22/02/2023	9764030	333.0	9763697.0	116.22336
9	H	Blank	22/02/2023	710			
10	H	Blank	22/02/2023	154			
11	H	Blank	22/02/2023	144			
12	H	Blank	22/02/2023	324			
1	G	**Reference Control**	15/02/2023	6599570	260.5	6599309.5	91.37762
2	G		15/02/2023	7127740	260.5	7127479.5	98.69095
3	G		15/02/2023	6915800	260.5	6915539.5	95.75631
4	G		15/02/2023	8246010	260.5	8245749.5	114.17512
1	G		17/02/2023	7941030	231.5	7940798.5	97.87416
2	G		17/02/2023	8311510	231.5	8311278.5	102.44051
3	G		17/02/2023	8386730	231.5	8386498.5	103.36763
4	G		17/02/2023	7814750	231.5	7814518.5	96.31770
1	G		22/02/2023	7541020	333.0	7540687.0	89.76149
2	G		22/02/2023	8662790	333.0	8662457.0	103.11461
3	G		22/02/2023	9124790	333.0	9124457.0	108.61409
4	G		22/02/2023	8275950	333.0	8275617.0	98.50982
5	G	**Medium Control**	15/02/2023	5776650	260.5	5776389.5	79.98302
6	G		15/02/2023	6016700	260.5	6016439.5	83.30689
7	G		15/02/2023	5762360	260.5	5762099.5	79.78516
8	G		15/02/2023	5864800	260.5	5864539.5	81.20360
5	G		17/02/2023	7051710	231.5	7051478.5	86.91287
6	G		17/02/2023	7333650	231.5	7333418.5	90.38791
7	G		17/02/2023	7036480	231.5	7036248.5	86.72515
8	G		17/02/2023	6532390	231.5	6532158.5	80.51200

5	G		22/02/2023	8107880	333.0	8107547.0	96.50917
6	G		22/02/2023	8152610	333.0	8152277.0	97.04162
7	G		22/02/2023	8303890	333.0	8303557.0	98.84240
8	G		22/02/2023	8722690	333.0	8722357.0	103.82764
9	G	**Positive Control**	15/02/2023	18097	260.5	17836.9	0.24698
10	G		15/02/2023	16355	260.5	16094.5	0.22285
11	G		15/02/2023	10178	260.5	9917.7	0.13733
12	G		15/02/2023	7604	260.5	7343.9	0.10169
9	G		17/02/2023	11305	231.5	11073.7	0.13649
10	G		17/02/2023	11445	231.5	11213.9	0.13822
11	G		17/02/2023	8805	231.5	8573.7	0.10567
12	G		17/02/2023	7548	231.5	7316.8	0.09018
9	G		22/02/2023	14869	333.0	14536.1	0.17303
10	G		22/02/2023	13431	333.0	13098.4	0.15592
11	G		22/02/2023	12404	333.0	12071.3	0.14369
12	G		22/02/2023	7768	333.0	7435.5	0.08851

KSL tested on hMSCs in the presence of FBS

Well	Row	KSL [µg/mL]	Data	Sample (RLU)	Blank (RLU)	Sample-Blank	Cell activity (%)
1	A	450.0	24/05/2023	26505	568.0	25936.8	0.44122
1	B	450.0	24/05/2023	34681	568.0	34113.3	0.58032
1	C	450.0	24/05/2023	32377	568.0	31808.9	0.54112
2	A	225.0	24/05/2023	880012	568.0	879444.0	14.96067
2	B	225.0	24/05/2023	1838360	568.0	1837792.0	31.26361
2	C	225.0	24/05/2023	1541930	568.0	1541362.0	26.22089

3	A	112.5	24/05/2023	5100020	568.0	5099452.0	86.74936
3	B	112.5	24/05/2023	5538220	568.0	5537652.0	94.20380
3	C	112.5	24/05/2023	6363970	568.0	6363402.0	108.25105
4	A	56.3	24/05/2023	5187960	568.0	5187392.0	88.24535
4	B	56.3	24/05/2023	5697070	568.0	5696502.0	96.90607
4	C	56.3	24/05/2023	5698200	568.0	5697632.0	96.92530
5	A	28.1	24/05/2023	5755240	568.0	5754672.0	97.89563
5	B	28.1	24/05/2023	6057520	568.0	6056952.0	103.03787
5	C	28.1	24/05/2023	5816550	568.0	5815982.0	98.93861
6	A	14.1	24/05/2023	5360580	568.0	5360012.0	91.18187
6	B	14.1	24/05/2023	5598830	568.0	5598262.0	95.23486
6	C	14.1	24/05/2023	5899690	568.0	5899122.0	100.35295
7	A	7.0	24/05/2023	5000500	568.0	4999932.0	85.05637
7	B	7.0	24/05/2023	5912660	568.0	5912092.0	100.57359
7	C	7.0	24/05/2023	5672420	568.0	5671852.0	96.48674
8	A	3.5	24/05/2023	5495580	568.0	5495012.0	93.47843
8	B	3.5	24/05/2023	6163930	568.0	6163362.0	104.84807
8	C	3.5	24/05/2023	6043720	568.0	6043152.0	102.80311
9	A	1.8	24/05/2023	5908360	568.0	5907792.0	100.50044
9	B	1.8	24/05/2023	6222260	568.0	6221692.0	105.84035
9	C	1.8	24/05/2023	5902330	568.0	5901762.0	100.39786
10	A	0.9	24/05/2023	6492470	568.0	6491902.0	110.43703
10	B	0.9	24/05/2023	6353220	568.0	6352652.0	108.06817
10	C	0.9	24/05/2023	5769360	568.0	5768792.0	98.13584
11	A	0.4	24/05/2023	5026940	568.0	5026372.0	85.50615
11	B	0.4	24/05/2023	5261460	568.0	5260892.0	89.49569
11	C	0.4	24/05/2023	5461820	568.0	5461252.0	92.90412
12	A	0.2	24/05/2023	4587860	568.0	4587292.0	78.03674

12	B	0.2	24/05/2023	4960550	568.0	4959982.0	84.37676
12	C	0.2	24/05/2023	5513440	568.0	5512872.0	93.78225
9	H	Blank	24/05/2023	920			
10	H	Blank	24/05/2023	404			
11	H	Blank	24/05/2023	336			
12	H	Blank	24/05/2023	612			
1	A	450	25/05/2023	30027	462.0	29564.9	0.61015
1	B	450	25/05/2023	45043	462.0	44581.0	0.92004
1	C	450	25/05/2023	31479	462.0	31016.6	0.64011
2	A	225	25/05/2023	1562690	462.0	1562228.0	32.24050
2	B	225	25/05/2023	2189750	462.0	2189288.0	45.18146
2	C	225	25/05/2023	2275610	462.0	2275148.0	46.95339
3	A	112.5	25/05/2023	4851660	462.0	4851198.0	100.11665
3	B	112.5	25/05/2023	5951900	462.0	5951438.0	122.82287
3	C	112.5	25/05/2023	5701640	462.0	5701178.0	117.65813
4	A	56.25	25/05/2023	5110560	462.0	5110098.0	105.45971
4	B	56.25	25/05/2023	5608740	462.0	5608278.0	115.74090
4	C	56.25	25/05/2023	5335340	462.0	5334878.0	110.09861
5	A	28.1	25/05/2023	5123150	462.0	5122688.0	105.71953
5	B	28.1	25/05/2023	5670150	462.0	5669688.0	117.00825
5	C	28.1	25/05/2023	5352470	462.0	5352008.0	110.45213
6	A	14.1	25/05/2023	5279290	462.0	5278828.0	108.94187
6	B	14.1	25/05/2023	5494860	462.0	5494398.0	113.39070
6	C	14.1	25/05/2023	5407190	462.0	5406728.0	111.58141
7	A	7.0	25/05/2023	5543300	462.0	5542838.0	114.39038
7	B	7.0	25/05/2023	5777220	462.0	5776758.0	119.21791
7	C	7.0	25/05/2023	5398390	462.0	5397928.0	111.39980
8	A	3.5	25/05/2023	5495890	462.0	5495428.0	113.41196

8	B	3.5	25/05/2023	5834730	462.0	5834268.0	120.40477
8	C	3.5	25/05/2023	5369910	462.0	5369448.0	110.81204
9	A	1.8	25/05/2023	5206370	462.0	5205908.0	107.43699
9	B	1.8	25/05/2023	5652690	462.0	5652228.0	116.64792
9	C	1.8	25/05/2023	5409820	462.0	5409358.0	111.63569
10	A	0.9	25/05/2023	5279990	462.0	5279528.0	108.95632
10	B	0.9	25/05/2023	5636370	462.0	5635908.0	116.31112
10	C	0.9	25/05/2023	5438100	462.0	5437638.0	112.21932
11	A	0.4	25/05/2023	4758890	462.0	4758428.0	98.20211
11	B	0.4	25/05/2023	5487170	462.0	5486708.0	113.23200
11	C	0.4	25/05/2023	5343450	462.0	5342988.0	110.26598
12	A	0.2	25/05/2023	4733810	462.0	4733348.0	97.68452
12	B	0.2	25/05/2023	5228030	462.0	5227568.0	107.88399
12	C	0.2	25/05/2023	4923950	462.0	4923488.0	101.60854
9	H	Blank	25/05/2023	684			
10	H	Blank	25/05/2023	312			
11	H	Blank	25/05/2023	400			
12	H	Blank	25/05/2023	452			
1	A	450	26/05/2023	42339	379.5	41959.9	0.68966
1	B	450	26/05/2023	45059	379.5	44679.6	0.73437
1	C	450	26/05/2023	55351	379.5	54971.8	0.90353
2	A	225	26/05/2023	1981040	379.5	1980660.5	32.55470
2	B	225	26/05/2023	2549490	379.5	2549110.5	41.89790
2	C	225	26/05/2023	1737520	379.5	1737140.5	28.55213
3	A	112.5	26/05/2023	6302490	379.5	6302110.5	103.58327
3	B	112.5	26/05/2023	7004210	379.5	7003830.5	115.11694
3	C	112.5	26/05/2023	6946260	379.5	6945880.5	114.16446
4	A	56.25	26/05/2023	6334320	379.5	6333940.5	104.10644

4	B	56.25	26/05/2023	6535610	379.5	6535230.5	107.41490
4	C	56.25	26/05/2023	7056800	379.5	7056420.5	115.98133
5	A	28.1	26/05/2023	6535780	379.5	6535400.5	107.41769
5	B	28.1	26/05/2023	6360710	379.5	6360330.5	104.54019
5	C	28.1	26/05/2023	6819540	379.5	6819160.5	112.08165
6	A	14.1	26/05/2023	6417020	379.5	6416640.5	105.46572
6	B	14.1	26/05/2023	6859070	379.5	6858690.5	112.73138
6	C	14.1	26/05/2023	6889200	379.5	6888820.5	113.22661
7	A	7.0	26/05/2023	6387920	379.5	6387540.5	104.98743
7	B	7.0	26/05/2023	7098240	379.5	7097860.5	116.66245
7	C	7.0	26/05/2023	6964650	379.5	6964270.5	114.46672
8	A	3.5	26/05/2023	6738170	379.5	6737790.5	110.74423
8	B	3.5	26/05/2023	7007840	379.5	7007460.5	115.17661
8	C	3.5	26/05/2023	7090670	379.5	7090290.5	116.53802
9	A	1.8	26/05/2023	6437530	379.5	6437150.5	105.80283
9	B	1.8	26/05/2023	6940350	379.5	6939970.5	114.06732
9	C	1.8	26/05/2023	6751800	379.5	6751420.5	110.96826
10	A	0.9	26/05/2023	6432690	379.5	6432310.5	105.72328
10	B	0.9	26/05/2023	6933690	379.5	6933310.5	113.95786
10	C	0.9	26/05/2023	6920890	379.5	6920510.5	113.74747
11	A	0.4	26/05/2023	6614380	379.5	6614000.5	108.70959
11	B	0.4	26/05/2023	7128290	379.5	7127910.5	117.15636
11	C	0.4	26/05/2023	6695540	379.5	6695160.5	110.04356
12	A	0.2	26/05/2023	6145370	379.5	6144990.5	101.00081
12	B	0.2	26/05/2023	6937370	379.5	6936990.5	114.01834
12	C	0.2	26/05/2023	6620350	379.5	6619970.5	108.80771
9	H	Blank	26/05/2023	790			
10	H	Blank	26/05/2023	204			

11	H	Blank	26/05/2023	242			
12	H	Blank	26/05/2023	282			
1	G	**Reference Control**	24/05/2023	5212640	568.0	5212072.0	88.66519
2	G		24/05/2023	5780970	568.0	5780402.0	98.33334
3	G		24/05/2023	6308510	568.0	6307942.0	107.30759
4	G		24/05/2023	6213650	568.0	6213082.0	105.69388
1	G		25/05/2023	4242410	462.0	4241948.0	87.54325
2	G		25/05/2023	4760260	462.0	4759798.0	98.23038
3	G		25/05/2023	5185230	462.0	5184768.0	107.00071
4	G		25/05/2023	5196130	462.0	5195668.0	107.22566
1	G		26/05/2023	5703750	379.5	5703370.5	93.74221
2	G		26/05/2023	5958570	379.5	5958190.5	97.93051
3	G		26/05/2023	6230010	379.5	6229630.5	102.39197
4	G		26/05/2023	6445590	379.5	6445210.5	105.93531
5	G	**Medium Control**	24/05/2023	6663750	568.0	6663182.0	113.35076
6	G		24/05/2023	7895850	568.0	7895282.0	134.31063
7	G		24/05/2023	7013560	568.0	7012992.0	119.30155
8	G		24/05/2023	6883590	568.0	6883022.0	117.09057
5	G		25/05/2023	5357220	462.0	5356758.0	110.55015
6	G		25/05/2023	5482410	462.0	5481948.0	113.13376
7	G		25/05/2023	5229450	462.0	5228988.0	107.91330
8	G		25/05/2023	5208520	462.0	5208058.0	107.48136
5	G		26/05/2023	6552200	379.5	6551820.5	107.68758
6	G		26/05/2023	6566500	379.5	6566120.5	107.92262
7	G		26/05/2023	6717030	379.5	6716650.5	110.39677
8	G		26/05/2023	6794730	379.5	6794350.5	111.67387
9	G	**Positive Control**	24/05/2023	5493	568.0	4925.2	0.08379

10	G		24/05/2023	3525	568.0	2956.5	0.05029
11	G		24/05/2023	3216	568.0	2648.4	0.04505
12	G		24/05/2023	2658	568.0	2090.3	0.03556
9	G		25/05/2023	3891	462.0	3428.6	0.07076
10	G		25/05/2023	2614	462.0	2152.3	0.04442
11	G		25/05/2023	2526	462.0	2064.3	0.04260
12	G		25/05/2023	2058	462.0	1596.2	0.03294
9	G		26/05/2023	4823	379.5	4443.4	0.07303
10	G		26/05/2023	3146	379.5	2766.9	0.04548
11	G		26/05/2023	2908	379.5	2528.8	0.04156
12	G		26/05/2023	2166	379.5	1786.7	0.02937

KSL tested on hMSCs in the absence of FBS

Well	Row	KSL [ug/ml]	Date	Sample (RLU)	Blank (RLU)	Sample - Blank	Cell activity (%)
1	A	450.0	31/05/2023	2144	143.5	2000.7	0.04142
1	B	450.0	31/05/2023	3432	143.5	3289.0	0.06810
1	C	450.0	31/05/2023	3096	143.5	2952.9	0.06114
2	A	225.0	31/05/2023	11235	143.5	11091.7	0.22965
2	B	225.0	31/05/2023	10825	143.5	10681.3	0.22115
2	C	225.0	31/05/2023	15650	143.5	15506.5	0.32105
3	A	112.5	31/05/2023	2175470	143.5	2175326.5	45.03855
3	B	112.5	31/05/2023	2898940	143.5	2898796.5	60.01747
3	C	112.5	31/05/2023	3019550	143.5	3019406.5	62.51461
4	A	56.3	31/05/2023	4037570	143.5	4037426.5	83.59197
4	B	56.3	31/05/2023	4062930	143.5	4062786.5	84.11703

4	C	56.3	31/05/2023	4278000	143.5	4277856.5	88.56990
5	A	28.1	31/05/2023	3841040	143.5	3840896.5	79.52296
5	B	28.1	31/05/2023	4265080	143.5	4264936.5	88.30240
5	C	28.1	31/05/2023	4399540	143.5	4399396.5	91.08629
6	A	14.1	31/05/2023	3856070	143.5	3855926.5	79.83414
6	B	14.1	31/05/2023	3828390	143.5	3828246.5	79.26105
6	C	14.1	31/05/2023	4157840	143.5	4157696.5	86.08207
7	A	7.0	31/05/2023	3525220	143.5	3525076.5	72.98414
7	B	7.0	31/05/2023	3793670	143.5	3793526.5	78.54220
7	C	7.0	31/05/2023	4256290	143.5	4256146.5	88.12041
8	A	3.5	31/05/2023	3624220	143.5	3624076.5	75.03386
8	B	3.5	31/05/2023	3916330	143.5	3916186.5	81.08178
8	C	3.5	31/05/2023	4174350	143.5	4174206.5	86.42390
9	A	1.8	31/05/2023	3377710	143.5	3377566.5	69.93005
9	B	1.8	31/05/2023	3981290	143.5	3981146.5	82.42673
9	C	1.8	31/05/2023	3929340	143.5	3929196.5	81.35115
10	A	0.9	31/05/2023	3333240	143.5	3333096.5	69.00933
10	B	0.9	31/05/2023	3965520	143.5	3965376.5	82.10023
10	C	0.9	31/05/2023	3559320	143.5	3559176.5	73.69015
11	A	0.4	31/05/2023	3308290	143.5	3308146.5	68.49276
11	B	0.4	31/05/2023	3782830	143.5	3782686.5	78.31776
11	C	0.4	31/05/2023	3384620	143.5	3384476.5	70.07312
12	A	0.2	31/05/2023	3056900	143.5	3056756.5	63.28791
12	B	0.2	31/05/2023	3540600	143.5	3540456.5	73.30257
12	C	0.2	31/05/2023	3188120	143.5	3187976.5	66.00473
9	H	Blank	31/05/2023	350			
10	H	Blank	31/05/2023	70			
11	H	Blank	31/05/2023	58			

12	H	Blank	31/05/2023	96			
1	A	450	01/06/2023	4169	138.0	4030.7	0.08905
1	B	450	01/06/2023	6196	138.0	6057.6	0.13384
1	C	450	01/06/2023	5635	138.0	5497.3	0.12146
2	A	225	01/06/2023	123062	138.0	122924.0	2.71587
2	B	225	01/06/2023	204224	138.0	204086.0	4.50905
2	C	225	01/06/2023	171173	138.0	171035.0	3.77883
3	A	112.5	01/06/2023	3745260	138.0	3745122.0	82.74429
3	B	112.5	01/06/2023	5218530	138.0	5218392.0	115.29455
3	C	112.5	01/06/2023	5060260	138.0	5060122.0	111.79775
4	A	56.25	01/06/2023	5330620	138.0	5330482.0	117.77105
4	B	56.25	01/06/2023	5695350	138.0	5695212.0	125.82935
4	C	56.25	01/06/2023	5741360	138.0	5741222.0	126.84589
5	A	28.1	01/06/2023	4996740	138.0	4996602.0	110.39434
5	B	28.1	01/06/2023	5740960	138.0	5740822.0	126.83705
5	C	28.1	01/06/2023	5486540	138.0	5486402.0	121.21593
6	A	14.1	01/06/2023	5420490	138.0	5420352.0	119.75663
6	B	14.1	01/06/2023	5991010	138.0	5990872.0	132.36163
6	C	14.1	01/06/2023	5733940	138.0	5733802.0	126.68195
7	A	7.0	01/06/2023	5475000	138.0	5474862.0	120.96096
7	B	7.0	01/06/2023	5609590	138.0	5609452.0	123.93458
7	C	7.0	01/06/2023	5853510	138.0	5853372.0	129.32372
8	A	3.5	01/06/2023	5086380	138.0	5086242.0	112.37484
8	B	3.5	01/06/2023	5896240	138.0	5896102.0	130.26779
8	C	3.5	01/06/2023	5811440	138.0	5811302.0	128.39423
9	A	1.8	01/06/2023	5169980	138.0	5169842.0	114.22189
9	B	1.8	01/06/2023	5728540	138.0	5728402.0	126.56265
9	C	1.8	01/06/2023	6105390	138.0	6105252.0	134.88873

10	A	0.9	01/06/2023	5306530	138.0	5306392.0	117.23881
10	B	0.9	01/06/2023	5922010	138.0	5921872.0	130.83715
10	C	0.9	01/06/2023	5805730	138.0	5805592.0	128.26807
11	A	0.4	01/06/2023	5131190	138.0	5131052.0	113.36487
11	B	0.4	01/06/2023	5429640	138.0	5429502.0	119.95879
11	C	0.4	01/06/2023	5653390	138.0	5653252.0	124.90229
12	A	0.2	01/06/2023	4615800	138.0	4615662.0	101.97790
12	B	0.2	01/06/2023	5322430	138.0	5322292.0	117.59010
12	C	0.2	01/06/2023	5414080	138.0	5413942.0	119.61501
1	H	Blank	01/06/2023	340			
2	H	Blank	01/06/2023	64			
3	H	Blank	01/06/2023	80			
4	H	Blank	01/06/2023	68			
1	A	450.0	15/06/2023	8807	196.5	8610.7	0.16805
1	B	450.0	15/06/2023	6396	196.5	6199.2	0.12099
1	C	450.0	15/06/2023	9662	196.5	9465.3	0.18473
2	A	225.0	15/06/2023	69978	196.5	69781.7	1.36192
2	B	225.0	15/06/2023	108944	196.5	108747.5	2.12242
2	C	225.0	15/06/2023	149082	196.5	148885.5	2.90579
3	A	112.5	15/06/2023	4844580	196.5	4844383.5	94.54751
3	B	112.5	15/06/2023	5322530	196.5	5322333.5	103.87562
3	C	112.5	15/06/2023	5931600	196.5	5931403.5	115.76280
4	A	56.3	15/06/2023	6082410	196.5	6082213.5	118.70615
4	B	56.3	15/06/2023	6213420	196.5	6213223.5	121.26306
4	C	56.3	15/06/2023	7065260	196.5	7065063.5	137.88837
5	A	28.1	15/06/2023	5852680	196.5	5852483.5	114.22253
5	B	28.1	15/06/2023	6366120	196.5	6365923.5	124.24330
5	C	28.1	15/06/2023	7201880	196.5	7201683.5	140.55477

6	A	14.1	15/06/2023	6114500	196.5	6114303.5	119.33245
6	B	14.1	15/06/2023	6760830	196.5	6760633.5	131.94683
6	C	14.1	15/06/2023	6959600	196.5	6959403.5	135.82621
7	A	7.0	15/06/2023	6296900	196.5	6296703.5	122.89234
7	B	7.0	15/06/2023	6621120	196.5	6620923.5	129.22012
7	C	7.0	15/06/2023	6727230	196.5	6727033.5	131.29106
8	A	3.5	15/06/2023	6129200	196.5	6129003.5	119.61935
8	B	3.5	15/06/2023	6593130	196.5	6592933.5	128.67384
8	C	3.5	15/06/2023	6402800	196.5	6402603.5	124.95918
9	A	1.8	15/06/2023	6105540	196.5	6105343.5	119.15758
9	B	1.8	15/06/2023	6217580	196.5	6217383.5	121.34425
9	C	1.8	15/06/2023	6694040	196.5	6693843.5	130.64329
10	A	0.9	15/06/2023	5908490	196.5	5908293.5	115.31177
10	B	0.9	15/06/2023	6391170	196.5	6390973.5	124.73220
10	C	0.9	15/06/2023	6627080	196.5	6626883.5	129.33644
11	A	0.4	15/06/2023	5931050	196.5	5930853.5	115.75207
11	B	0.4	15/06/2023	6286850	196.5	6286653.5	122.69619
11	C	0.4	15/06/2023	6436660	196.5	6436463.5	125.62002
12	A	0.2	15/06/2023	6185960	196.5	6185763.5	120.72713
12	B	0.2	15/06/2023	6255990	196.5	6255793.5	122.09390
12	C	0.2	15/06/2023	6642860	196.5	6642663.5	129.64442
9	H	Blank	15/06/2023	472			
10	H	Blank	15/06/2023	64			
11	H	Blank	15/06/2023	78			
12	H	Blank	15/06/2023	172			
1	A	450.0	22/06/2023	6700	167.5	6532.3	0.11471
1	B	450.0	22/06/2023	8683	167.5	8515.6	0.14953
1	C	450.0	22/06/2023	6570	167.5	6402.3	0.11242

2	A	225.0	22/06/2023	166053	167.5	165885.5	2.91292
2	B	225.0	22/06/2023	103163	167.5	102995.5	1.80858
2	C	225.0	22/06/2023	150693	167.5	150525.5	2.64320
3	A	112.5	22/06/2023	5011690	167.5	5011522.5	88.00146
3	B	112.5	22/06/2023	5835700	167.5	5835532.5	102.47093
3	C	112.5	22/06/2023	5045760	167.5	5045592.5	88.59972
4	A	56.3	22/06/2023	6503640	167.5	6503472.5	114.19984
4	B	56.3	22/06/2023	6618010	167.5	6617842.5	116.20816
4	C	56.3	22/06/2023	6799360	167.5	6799192.5	119.39263
5	A	28.1	22/06/2023	6798910	167.5	6798742.5	119.38473
5	B	28.1	22/06/2023	6883620	167.5	6883452.5	120.87222
5	C	28.1	22/06/2023	7110070	167.5	7109902.5	124.84865
6	A	14.1	22/06/2023	6787870	167.5	6787702.5	119.19087
6	B	14.1	22/06/2023	7022400	167.5	7022232.5	123.30918
6	C	14.1	22/06/2023	6660090	167.5	6659922.5	116.94708
7	A	7.0	22/06/2023	7015730	167.5	7015562.5	123.19205
7	B	7.0	22/06/2023	6920790	167.5	6920622.5	121.52492
7	C	7.0	22/06/2023	6490160	167.5	6489992.5	113.96313
8	A	3.5	22/06/2023	6615720	167.5	6615552.5	116.16795
8	B	3.5	22/06/2023	6879480	167.5	6879312.5	120.79953
8	C	3.5	22/06/2023	6692880	167.5	6692712.5	117.52286
9	A	1.8	22/06/2023	6546500	167.5	6546332.5	114.95245
9	B	1.8	22/06/2023	6610790	167.5	6610622.5	116.08138
9	C	1.8	22/06/2023	6581230	167.5	6581062.5	115.56231
10	A	0.9	22/06/2023	6589260	167.5	6589092.5	115.70331
10	B	0.9	22/06/2023	6625080	167.5	6624912.5	116.33231
10	C	0.9	22/06/2023	6723650	167.5	6723482.5	118.06318
11	A	0.4	22/06/2023	6537670	167.5	6537502.5	114.79740

11	B	0.4	22/06/2023	7173810	167.5	7173642.5	125.96791
11	C	0.4	22/06/2023	6284140	167.5	6283972.5	110.34546
12	A	0.2	22/06/2023	6589210	167.5	6589042.5	115.70243
12	B	0.2	22/06/2023	6529080	167.5	6528912.5	114.64656
12	C	0.2	22/06/2023	6246930	167.5	6246762.5	109.69206
9	H	Blank	22/06/2023	386			
10	H	Blank	22/06/2023	92			
11	H	Blank	22/06/2023	66			
12	H	Blank	22/06/2023	126			
1	G	**Reference Control**	31/05/2023	3488430	143.5	3488286.5	72.22243
2	G		31/05/2023	3775550	143.5	3775406.5	78.16704
3	G		31/05/2023	5396030	143.5	5395886.5	111.71789
4	G		31/05/2023	6660250	143.5	6660106.5	137.89264
1	G		01/06/2023	4134710	138.0	4134572.0	91.34875
2	G		01/06/2023	4589200	138.0	4589062.0	101.39020
3	G		01/06/2023	4789830	138.0	4789692.0	105.82290
4	G		01/06/2023	4591370	138.0	4591232.0	101.43815
1	G		15/06/2023	4703750	196.5	4703553.5	91.79894
2	G		15/06/2023	5092760	196.5	5092563.5	99.39122
3	G		15/06/2023	4979100	196.5	4978903.5	97.17292
4	G		15/06/2023	5720200	196.5	5720003.5	111.63692
1	G		22/06/2023	5599540	167.5	5599372.5	98.32400
2	G		22/06/2023	5649900	167.5	5649732.5	99.20832
3	G		22/06/2023	6079320	167.5	6079152.5	106.74886
4	G		22/06/2023	5451180	167.5	5451012.5	95.71883
5	G	**Medium Control**	31/05/2023	4130120	143.5	4129976.5	85.50815
6	G		31/05/2023	4410490	143.5	4410346.5	91.31301

7 G		31/05/2023	4465900	143.5	4465756.5	92.46023
8 G		31/05/2023	4311130	143.5	4310986.5	89.25583
5 G		01/06/2023	4603120	138.0	4602982.0	101.69775
6 G		01/06/2023	4259770	138.0	4259632.0	94.11181
7 G		01/06/2023	4525110	138.0	4524972.0	99.97421
8 G		01/06/2023	4704630	138.0	4704492.0	103.94050
5 G		15/06/2023	4634030	196.5	4633833.5	90.43822
6 G		15/06/2023	4643900	196.5	4643703.5	90.63085
7 G		15/06/2023	4578360	196.5	4578163.5	89.35171
8 G		15/06/2023	4483650	196.5	4483453.5	87.50326
5 G		22/06/2023	5983270	167.5	5983102.5	105.06223
6 G		22/06/2023	6192210	167.5	6192042.5	108.73118
7 G		22/06/2023	6010370	167.5	6010202.5	105.53811
8 G		22/06/2023	5805770	167.5	5805602.5	101.94537
9 G	**Positive Control**	31/05/2023	3212	143.5	3068.9	0.06354
10 G		31/05/2023	1948	143.5	1804.7	0.03736
11 G		31/05/2023	1968	143.5	1824.7	0.03778
12 G		31/05/2023	1504	143.5	1360.6	0.02817
9 G		01/06/2023	3781	138.0	3642.6	0.08048
10 G		01/06/2023	2526	138.0	2388.3	0.05277
11 G		01/06/2023	2806	138.0	2668.3	0.05895
12 G		01/06/2023	2248	138.0	2110.2	0.04662
9 G		15/06/2023	4083	196.5	3886.2	0.07585
10 G		15/06/2023	2814	196.5	2617.8	0.05109
11 G		15/06/2023	3140	196.5	2943.9	0.05746
12 G		15/06/2023	2424	196.5	2227.7	0.04348

9	G		22/06/2023	4927	167.5	4759.5	0.08358
10	G		22/06/2023	3300	167.5	3132.9	0.05501
11	G		22/06/2023	3537	167.5	3369.0	0.05916
12	G		22/06/2023	2750	167.5	2582.8	0.04535

KSL-W tested on MG63 in the presence of FBS

Well	Row	KSL-W [μg/mL]	Data	Sample (RLU)	Blank (RLU)	Sample-Blank	Cell activity (%)
1	D	450.0	11/11/2022	3154	490.0	2664.4	0.02358
1	E	450.0	11/11/2022	2146	490.0	1656.2	0.01465
1	F	450.0	11/11/2022	4451	490.0	3960.8	0.03505
2	D	225.0	11/11/2022	8003	490.0	7512.6	0.06648
2	E	225.0	11/11/2022	7704	490.0	7214.4	0.06384
2	F	225.0	11/11/2022	69234	490.0	68744.0	0.60828
3	D	112.5	11/11/2022	8154460	490.0	8153970.0	72.15014
3	E	112.5	11/11/2022	9856630	490.0	9856140.0	87.21173
3	F	112.5	11/11/2022	6594030	490.0	6593540.0	58.34272
4	D	56.3	11/11/2022	11343100	490.0	11342610.0	100.36471
4	E	56.3	11/11/2022	12536900	490.0	12536410.0	110.92801
4	F	56.3	11/11/2022	10753600	490.0	10753110.0	95.14854
5	D	28.1	11/11/2022	12218300	490.0	12217810.0	108.10889
5	E	28.1	11/11/2022	12807000	490.0	12806510.0	113.31798
5	F	28.1	11/11/2022	11547800	490.0	11547310.0	102.17599
6	D	14.1	11/11/2022	12922900	490.0	12922410.0	114.34352
6	E	14.1	11/11/2022	13133800	490.0	13133310.0	116.20966
6	F	14.1	11/11/2022	11780200	490.0	11779710.0	104.23238
7	D	7.0	11/11/2022	13253100	490.0	13252610.0	117.26528

7	E	7.0	11/11/2022	13775000	490.0	13774510.0	121.88330
7	F	7.0	11/11/2022	12203500	490.0	12203010.0	107.97793
8	D	3.5	11/11/2022	13482400	490.0	13481910.0	119.29424
8	E	3.5	11/11/2022	13990700	490.0	13990210.0	123.79191
8	F	3.5	11/11/2022	13052400	490.0	13051910.0	115.48940
9	D	1.8	11/11/2022	12644800	490.0	12644310.0	111.88276
9	E	1.8	11/11/2022	12437500	490.0	12437010.0	110.04847
9	F	1.8	11/11/2022	11418800	490.0	11418310.0	101.03454
10	D	0.9	11/11/2022	12953000	490.0	12952510.0	114.60986
10	E	0.9	11/11/2022	13124800	490.0	13124310.0	116.13003
10	F	0.9	11/11/2022	11489100	490.0	11488610.0	101.65659
11	D	0.4	11/11/2022	12321700	490.0	12321210.0	109.02382
11	E	0.4	11/11/2022	12785000	490.0	12784510.0	113.12332
11	F	0.4	11/11/2022	11910500	490.0	11910010.0	105.38533
12	D	0.2	11/11/2022	10019300	490.0	10018810.0	88.65111
12	E	0.2	11/11/2022	12347400	490.0	12346910.0	109.25123
12	F	0.2	11/11/2022	10842800	490.0	10842310.0	95.93782
9	H	Blank	11/11/2022	1040			
10	H	Blank	11/11/2022	268			
11	H	Blank	11/11/2022	232			
12	H	Blank	11/11/2022	420			
1	D	450	16/11/2022	3368	533.0	2835.5	0.02639
1	E	450	16/11/2022	2420	533.0	1887.2	0.01756
1	F	450	16/11/2022	4231	533.0	3697.7	0.03441
2	D	225	16/11/2022	10499	533.0	9965.5	0.09275
2	E	225	16/11/2022	11772	533.0	11238.7	0.10460
2	F	225	16/11/2022	8563	533.0	8030.0	0.07473
3	D	112.5	16/11/2022	9796150	533.0	9795617.0	91.16642

3	E	112.5	16/11/2022	8030940	533.0	8030407.0	74.73786
3	F	112.5	16/11/2022	9413860	533.0	9413327.0	87.60850
4	D	56.25	16/11/2022	12695200	533.0	12694667.0	118.14747
4	E	56.25	16/11/2022	12314800	533.0	12314267.0	114.60714
4	F	56.25	16/11/2022	11721300	533.0	11720767.0	109.08352
5	D	28.1	16/11/2022	11869800	533.0	11869267.0	110.46559
5	E	28.1	16/11/2022	12858400	533.0	12857867.0	119.66635
5	F	28.1	16/11/2022	12145800	533.0	12145267.0	113.03428
6	D	14.1	16/11/2022	12160000	533.0	12159467.0	113.16644
6	E	14.1	16/11/2022	12032200	533.0	12031667.0	111.97702
6	F	14.1	16/11/2022	12124400	533.0	12123867.0	112.83511
7	D	7.0	16/11/2022	11828200	533.0	11827667.0	110.07842
7	E	7.0	16/11/2022	12434400	533.0	12433867.0	115.72024
7	F	7.0	16/11/2022	11666800	533.0	11666267.0	108.57629
8	D	3.5	16/11/2022	12356400	533.0	12355867.0	114.99430
8	E	3.5	16/11/2022	12740900	533.0	12740367.0	118.57279
8	F	3.5	16/11/2022	12198800	533.0	12198267.0	113.52754
9	D	1.8	16/11/2022	12414100	533.0	12413567.0	115.53131
9	E	1.8	16/11/2022	12941800	533.0	12941267.0	120.44254
9	F	1.8	16/11/2022	11602500	533.0	11601967.0	107.97786
10	D	0.9	16/11/2022	13304600	533.0	13304067.0	123.81907
10	E	0.9	16/11/2022	12642800	533.0	12642267.0	117.65979
10	F	0.9	16/11/2022	12404400	533.0	12403867.0	115.44103
11	D	0.4	16/11/2022	13200500	533.0	13199967.0	122.85022
11	E	0.4	16/11/2022	13174700	533.0	13174167.0	122.61010
11	F	0.4	16/11/2022	13243600	533.0	13243067.0	123.25135
12	D	0.2	16/11/2022	12411700	533.0	12411167.0	115.50897
12	E	0.2	16/11/2022	12674600	533.0	12674067.0	117.95574

12	F	0.2	16/11/2022	12632300	533.0	12631767.0	117.56206
9	H	Blank	16/11/2022	1088			
10	H	Blank	16/11/2022	292			
11	H	Blank	16/11/2022	380			
12	H	Blank	16/11/2022	372			
1	D	450	18/11/2022	5273	573.5	4699.6	0.03513
1	E	450	18/11/2022	3657	573.5	3083.0	0.02304
1	F	450	18/11/2022	5643	573.5	5069.8	0.03789
2	D	225	18/11/2022	444496	573.5	443922.5	3.31807
2	E	225	18/11/2022	11730	573.5	11156.1	0.08339
2	F	225	18/11/2022	44593	573.5	44019.9	0.32902
3	D	112.5	18/11/2022	12648500	573.5	12647926.5	94.53601
3	E	112.5	18/11/2022	11811800	573.5	11811226.5	88.28215
3	F	112.5	18/11/2022	11622200	573.5	11621626.5	86.86500
4	D	56.25	18/11/2022	14030700	573.5	14030126.5	104.86716
4	E	56.25	18/11/2022	14130900	573.5	14130326.5	105.61610
4	F	56.25	18/11/2022	13801500	573.5	13800926.5	103.15402
5	D	28.1	18/11/2022	15994600	573.5	15994026.5	119.54619
5	E	28.1	18/11/2022	15246500	573.5	15245926.5	113.95457
5	F	28.1	18/11/2022	14921100	573.5	14920526.5	111.52239
6	D	14.1	18/11/2022	15365000	573.5	15364426.5	114.84029
6	E	14.1	18/11/2022	14950700	573.5	14950126.5	111.74363
6	F	14.1	18/11/2022	14156700	573.5	14156126.5	105.80894
7	D	7.0	18/11/2022	15672400	573.5	15671826.5	117.13793
7	E	7.0	18/11/2022	15142400	573.5	15141826.5	113.17648
7	F	7.0	18/11/2022	15163600	573.5	15163026.5	113.33494
8	D	3.5	18/11/2022	15911600	573.5	15911026.5	118.92581
8	E	3.5	18/11/2022	15389500	573.5	15388926.5	115.02341

8	F	3.5	18/11/2022	15829000	573.5	15828426.5	118.30842
9	D	1.8	18/11/2022	16494000	573.5	16493426.5	123.27892
9	E	1.8	18/11/2022	13560400	573.5	13559826.5	101.35194
9	F	1.8	18/11/2022	14433300	573.5	14432726.5	107.87636
10	D	0.9	18/11/2022	14787000	573.5	14786426.5	110.52007
10	E	0.9	18/11/2022	14235800	573.5	14235226.5	106.40017
10	F	0.9	18/11/2022	14144200	573.5	14143626.5	105.71551
11	D	0.4	18/11/2022	14555800	573.5	14555226.5	108.79198
11	E	0.4	18/11/2022	14310900	573.5	14310326.5	106.96149
11	F	0.4	18/11/2022	14419700	573.5	14419126.5	107.77471
12	D	0.2	18/11/2022	14140100	573.5	14139526.5	105.68486
12	E	0.2	18/11/2022	13446200	573.5	13445626.5	100.49836
12	F	0.2	18/11/2022	13671900	574	13671326.5	102.18534
9	H	Blank	18/11/2022	1148			
10	H	Blank	18/11/2022	268			
11	H	Blank	18/11/2022	328			
12	H	Blank	18/11/2022	550			
1	G	**Reference Control**	11/11/2022	9561430	490.0	9560940.0	84.59966
2	G		11/11/2022	11409400	490.0	11408910.0	100.95137
3	G		11/11/2022	11662700	490.0	11662210.0	103.19268
4	G		11/11/2022	12574000	490.0	12573510.0	111.25629
1	G		16/11/2022	9439390	533.0	9438857.0	87.84610
2	G		16/11/2022	10912300	533.0	10911767.0	101.55427
3	G		16/11/2022	11467300	533.0	11466767.0	106.71957
4	G		16/11/2022	11162200	533.0	11161667.0	103.88005
1	G		18/11/2022	12161500	573.5	12160926.5	90.89596
2	G		18/11/2022	11021100	573.5	11020526.5	82.37212

3 G		18/11/2022	14298700	573.5	14298126.5	106.87031
4 G		18/11/2022	16036800	573.5	16036226.5	119.86161
5 G	**Medium Control**	11/11/2022	12373000	490.0	12372510.0	109.47775
6 G		11/11/2022	12572800	490.0	12572310.0	111.24567
7 G		11/11/2022	12951300	490.0	12950810.0	114.59482
8 G		11/11/2022	13098700	490.0	13098210.0	115.89908
5 G		16/11/2022	10636600	533.0	10636067.0	98.98837
6 G		16/11/2022	9561570	533.0	9561037.0	88.98322
7 G		16/11/2022	9133550	533.0	9133017.0	84.99969
8 G		16/11/2022	12942000	533.0	12941467.0	120.44440
5 G		18/11/2022	16838000	573.5	16837426.5	125.85012
6 G		18/11/2022	13500300	573.5	13499726.5	100.90272
7 G		18/11/2022	15933100	573.5	15932526.5	119.08651
8 G		18/11/2022	13558200	573.5	13557626.5	101.33549
9 G	**Positive Control**	11/11/2022	7780	490.0	7290.5	0.06451
10 G		11/11/2022	4691	490.0	4200.9	0.03717
11 G		11/11/2022	4597	490.0	4106.9	0.03634
12 G		11/11/2022	3286	490.0	2796.4	0.02474
9 G		16/11/2022	7378	533.0	6845.2	0.06371
10 G		16/11/2022	4399	533.0	3865.8	0.03598
11 G		16/11/2022	5121	533.0	4588.1	0.04270
12 G		16/11/2022	3358	533.0	2825.4	0.02630
9 G		18/11/2022	8701	573.5	8127.6	0.06075
10 G		18/11/2022	5417	573.5	4843.7	0.03620
11 G		18/11/2022	5299	573.5	4725.6	0.03532
12 G		18/11/2022	3737	573.5	3163.1	0.02364

KSL-W tested on MG63 in the absence of FBS

Well	Row	KSL-W [µg/mL]	Date	Sample (RLU)	Blank (RLU)	Sample - Blank	Cell activity (%)
1	D	450.0	03/02/2023	25004	310.0	24693.6	0.27332
1	E	450.0	03/02/2023	28387	310.0	28077.0	0.31077
1	F	450.0	03/02/2023	36314	310.0	36004.0	0.39851
2	D	225.0	03/02/2023	17242	310.0	16932.2	0.18741
2	E	225.0	03/02/2023	21086	310.0	20776.2	0.22996
2	F	225.0	03/02/2023	49490	310.0	49180.2	0.54435
3	D	112.5	03/02/2023	6662290	310.0	6661980.0	73.73805
3	E	112.5	03/02/2023	4504530	310.0	4504220.0	49.85491
3	F	112.5	03/02/2023	4227270	310.0	4226960.0	46.78606
4	D	56.3	03/02/2023	9707120	310.0	9706810.0	107.43971
4	E	56.3	03/02/2023	9640460	310.0	9640150.0	106.70189
4	F	56.3	03/02/2023	9646030	310.0	9645720.0	106.76354
5	D	28.1	03/02/2023	9332750	310.0	9332440.0	103.29600
5	E	28.1	03/02/2023	9286540	310.0	9286230.0	102.78453
5	F	28.1	03/02/2023	9428030	310.0	9427720.0	104.35061
6	D	14.1	03/02/2023	9667590	310.0	9667280.0	107.00217
6	E	14.1	03/02/2023	9754820	310.0	9754510.0	107.96768
6	F	14.1	03/02/2023	9254660	310.0	9254350.0	102.43166
7	D	7.0	03/02/2023	9992290	310.0	9991980.0	110.59611
7	E	7.0	03/02/2023	9683320	310.0	9683010.0	107.17628
7	F	7.0	03/02/2023	9539620	310.0	9539310.0	105.58574
8	D	3.5	03/02/2023	9861670	310.0	9861360.0	109.15035
8	E	3.5	03/02/2023	9715390	310.0	9715080.0	107.53125
8	F	3.5	03/02/2023	10136200	310.0	10135890.0	112.18898

9	D	1.8	03/02/2023	9926420	310.0	9926110.0	109.86703
9	E	1.8	03/02/2023	9615080	310.0	9614770.0	106.42097
9	F	1.8	03/02/2023	10240100	310.0	10239790.0	113.33899
10	D	0.9	03/02/2023	9698990	310.0	9698680.0	107.34973
10	E	0.9	03/02/2023	9395800	310.0	9395490.0	103.99387
10	F	0.9	03/02/2023	10133200	310.0	10132890.0	112.15577
11	D	0.4	03/02/2023	9649720	310.0	9649410.0	106.80438
11	E	0.4	03/02/2023	9510160	310.0	9509850.0	105.25966
11	F	0.4	03/02/2023	10836600	310.0	10836290.0	119.94135
12	D	0.2	03/02/2023	9813060	310.0	9812750.0	108.61231
12	E	0.2	03/02/2023	9565420	310.0	9565110.0	105.87131
12	F	0.2	03/02/2023	11394200	310.0	11393890.0	126.11314
9	H	Blank	03/02/2023	726			
10	H	Blank	03/02/2023	122			
11	H	Blank	03/02/2023	124			
12	H	Blank	03/02/2023	268			
1	D	450	08/02/2023	24795	2661.3	22133.9	0.28384
1	E	450	08/02/2023	27533	2661.3	24871.7	0.31895
1	F	450	08/02/2023	23903	2661.3	21242.1	0.27241
2	D	225	08/02/2023	21048	2661.3	18386.9	0.23579
2	E	225	08/02/2023	13752	2661.3	11090.4	0.14222
2	F	225	08/02/2023	18085	2661.3	15424.1	0.19780
3	D	112.5	08/02/2023	4400860	2661.3	4398198.7	56.40230
3	E	112.5	08/02/2023	3953050	2661.3	3950388.7	50.65961
3	F	112.5	08/02/2023	2920130	2661.3	2917468.7	37.41349
4	D	56.25	08/02/2023	8486170	2661.3	8483508.7	108.79214
4	E	56.25	08/02/2023	8862780	2661.3	8860118.7	113.62177
4	F	56.25	08/02/2023	7269280	2661.3	7266618.7	93.18679

5	D	28.1	08/02/2023	8669060	2661.3	8666398.7	111.13751
5	E	28.1	08/02/2023	8376650	2661.3	8373988.7	107.38766
5	F	28.1	08/02/2023	6938780	2661.3	6936118.7	88.94848
6	D	14.1	08/02/2023	9560060	2661.3	9557398.7	122.56365
6	E	14.1	08/02/2023	8647320	2661.3	8644658.7	110.85872
6	F	14.1	08/02/2023	7166940	2661.3	7164278.7	91.87439
7	D	7.0	08/02/2023	8709890	2661.3	8707228.7	111.66111
7	E	7.0	08/02/2023	8839280	2661.3	8836618.7	113.32040
7	F	7.0	08/02/2023	7263480	2661.3	7260818.7	93.11241
8	D	3.5	08/02/2023	9709250	2661.3	9706588.7	124.47686
8	E	3.5	08/02/2023	8524110	2661.3	8521448.7	109.27868
8	F	3.5	08/02/2023	7130160	2661.3	7127498.7	91.40272
9	D	1.8	08/02/2023	8295420	2661.3	8292758.7	106.34597
9	E	1.8	08/02/2023	8579670	2661.3	8577008.7	109.99118
9	F	1.8	08/02/2023	6963440	2661.3	6960778.7	89.26471
10	D	0.9	08/02/2023	8295370	2661.3	8292708.7	106.34533
10	E	0.9	08/02/2023	8324750	2661.3	8322088.7	106.72209
10	F	0.9	08/02/2023	7196640	2661.3	7193978.7	92.25526
11	D	0.4	08/02/2023	7981240	2661.3	7978578.7	102.31694
11	E	0.4	08/02/2023	8327450	2661.3	8324788.7	106.75672
11	F	0.4	08/02/2023	7669480	2661.3	7666818.7	98.31894
12	D	0.2	08/02/2023	7853380	2661.3	7850718.7	100.67727
12	E	0.2	08/02/2023	8412460	2661.3	8409798.7	107.84688
12	F	0.2	08/02/2023	6488780	2661.3	6486118.7	83.17770
1	H	Blank	08/02/2023	1976			
2	H	Blank	08/02/2023	3092			
3	H	Blank	08/02/2023	2938			
4	H	Blank	08/02/2023	2638			

1	D	450.0	10/02/2023	13634	357.5	13276.1	0.18615
1	E	450.0	10/02/2023	32092	357.5	31734.7	0.44497
1	F	450.0	10/02/2023	39363	357.5	39005.9	0.54692
2	D	225.0	10/02/2023	10609	357.5	10251.1	0.14374
2	E	225.0	10/02/2023	17120	357.5	16762.5	0.23504
2	F	225.0	10/02/2023	25082	357.5	24724.3	0.34667
3	D	112.5	10/02/2023	4439210	357.5	4438852.5	62.23980
3	E	112.5	10/02/2023	3590350	357.5	3589992.5	50.33743
3	F	112.5	10/02/2023	3388310	357.5	3387952.5	47.50451
4	D	56.3	10/02/2023	9314490	357.5	9314132.5	130.59902
4	E	56.3	10/02/2023	9284070	357.5	9283712.5	130.17248
4	F	56.3	10/02/2023	8949140	357.5	8948782.5	125.47623
5	D	28.1	10/02/2023	8779760	357.5	8779402.5	123.10125
5	E	28.1	10/02/2023	8630250	357.5	8629892.5	121.00488
5	F	28.1	10/02/2023	8254400	357.5	8254042.5	115.73486
6	D	14.1	10/02/2023	9140580	357.5	9140222.5	128.16052
6	E	14.1	10/02/2023	8937850	357.5	8937492.5	125.31792
6	F	14.1	10/02/2023	8589760	357.5	8589402.5	120.43714
7	D	7.0	10/02/2023	8837260	357.5	8836902.5	123.90749
7	E	7.0	10/02/2023	8906900	357.5	8906542.5	124.88395
7	F	7.0	10/02/2023	8914350	357.5	8913992.5	124.98841
8	D	3.5	10/02/2023	10887000	357.5	10886642.5	152.64812
8	E	3.5	10/02/2023	8618170	357.5	8617812.5	120.83550
8	F	3.5	10/02/2023	8300870	357.5	8300512.5	116.38645
9	D	1.8	10/02/2023	10417100	357.5	10416742.5	146.05937
9	E	1.8	10/02/2023	8424090	357.5	8423732.5	118.11419
9	F	1.8	10/02/2023	8864260	357.5	8863902.5	124.28607
10	D	0.9	10/02/2023	9135850	357.5	9135492.5	128.09420

10	E	0.9	10/02/2023	9081990	357.5	9081632.5	127.33900
10	F	0.9	10/02/2023	8854120	357.5	8853762.5	124.14389
11	D	0.4	10/02/2023	8705190	357.5	8704832.5	122.05566
11	E	0.4	10/02/2023	8711780	357.5	8711422.5	122.14806
11	F	0.4	10/02/2023	9129820	357.5	9129462.5	128.00965
12	D	0.2	10/02/2023	8958170	357.5	8957812.5	125.60284
12	E	0.2	10/02/2023	8838800	357.5	8838442.5	123.92908
12	F	0.2	10/02/2023	9252090	357.5	9251732.5	129.72407
9	H	Blank	10/02/2023	1044			
10	H	Blank	10/02/2023	118			
11	H	Blank	10/02/2023	84			
12	H	Blank	10/02/2023	184			
1	D	450.0	19/04/2024	26244	254.5	25989.7	0.39498
1	E	450.0	19/04/2024	25513	254.5	25258.2	0.38386
1	F	450.0	19/04/2024	25501	254.5	25246.1	0.38368
2	D	225.0	19/04/2024	23607	254.5	23352.3	0.35489
2	E	225.0	19/04/2024	19089	254.5	18834.4	0.28623
2	F	225.0	19/04/2024	27886	254.5	27631.3	0.41992
3	D	112.5	19/04/2024	3465260	254.5	3465005.5	52.65917
3	E	112.5	19/04/2024	3460070	254.5	3459815.5	52.58030
3	F	112.5	19/04/2024	2077480	254.5	2077225.5	31.56849
4	D	56.3	19/04/2024	7385670	254.5	7385415.5	112.23933
4	E	56.3	19/04/2024	8278990	254.5	8278735.5	125.81549
4	F	56.3	19/04/2024	6631310	254.5	6631055.5	100.77499
5	D	28.1	19/04/2024	7315470	254.5	7315215.5	111.17247
5	E	28.1	19/04/2024	7815770	254.5	7815515.5	118.77574
5	F	28.1	19/04/2024	7381800	254.5	7381545.5	112.18051
6	D	14.1	19/04/2024	6781860	254.5	6781605.5	103.06297

6	E	14.1	19/04/2024	7732360	254.5	7732105.5	117.50812
6	F	14.1	19/04/2024	7161170	254.5	7160915.5	108.82750
7	D	7.0	19/04/2024	6867450	254.5	6867195.5	104.36371
7	E	7.0	19/04/2024	7012710	254.5	7012455.5	106.57129
7	F	7.0	19/04/2024	6465480	254.5	6465225.5	98.25480
8	D	3.5	19/04/2024	6553060	254.5	6552805.5	99.58579
8	E	3.5	19/04/2024	6460570	254.5	6460315.5	98.18018
8	F	3.5	19/04/2024	5690640	254.5	5690385.5	86.47923
9	D	1.8	19/04/2024	6378670	254.5	6378415.5	96.93551
9	E	1.8	19/04/2024	6332320	254.5	6332065.5	96.23111
9	F	1.8	19/04/2024	6474470	254.5	6474215.5	98.39143
10	D	0.9	19/04/2024	7148660	254.5	7148405.5	108.63738
10	E	0.9	19/04/2024	6896340	254.5	6896085.5	104.80277
10	F	0.9	19/04/2024	6167550	254.5	6167295.5	93.72703
11	D	0.4	19/04/2024	6903880	254.5	6903625.5	104.91736
11	E	0.4	19/04/2024	7105460	254.5	7105205.5	107.98085
11	F	0.4	19/04/2024	6486790	254.5	6486535.5	98.57866
12	D	0.2	19/04/2024	6868460	254.5	6868205.5	104.37906
12	E	0.2	19/04/2024	6344400	254.5	6344145.5	96.41470
12	F	0.2	19/04/2024	6975490	254.5	6975235.5	106.00564
9	H	Blank	19/04/2024	478			
10	H	Blank	19/04/2024	148			
11	H	Blank	19/04/2024	130			
12	H	Blank	19/04/2024	262			
1	G	**Reference Control**	03/02/2023	9270520	310.0	9270210.0	102.60721
2	G		03/02/2023	8686740	310.0	8686430.0	96.14565
3	G		03/02/2023	9199430	310.0	9199120.0	101.82035
4	G		03/02/2023	8983180	310.0	8982870.0	99.42679

1 G		08/02/2023	7886050	2661.3	7883388.7	101.09622
2 G		08/02/2023	7812290	2661.3	7809628.7	100.15033
3 G		08/02/2023	8036210	2661.3	8033548.7	103.02187
4 G		08/02/2023	7467720	2661.3	7465058.7	95.73158
1 G		10/02/2023	7159270	357.5	7158912.5	100.37939
2 G		10/02/2023	6586440	357.5	6586082.5	92.34740
3 G		10/02/2023	7149200	357.5	7148842.5	100.23819
4 G		10/02/2023	7633940	357.5	7633582.5	107.03502
1 G		19/04/2024	5769240	254.5	5768985.5	87.67375
2 G		19/04/2024	6218160	254.5	6217905.5	94.49618
3 G		19/04/2024	6974500	254.5	6974245.5	105.99060
4 G		19/04/2024	7359360	254.5	7359105.5	111.83948
5 G	**Medium Control**	03/02/2023	9163880	310.0	9163570.0	101.42687
6 G		03/02/2023	9113030	310.0	9112720.0	100.86403
7 G		03/02/2023	8924720	310.0	8924410.0	98.77973
8 G		03/02/2023	9271100	310.0	9270790.0	102.61363
5 G		08/02/2023	7710800	2661.3	7708138.7	98.84883
6 G		08/02/2023	7177000	2661.3	7174338.7	92.00340
7 G		08/02/2023	7640920	2661.3	7638258.7	97.95269
8 G		08/02/2023	7407250	2661.3	7404588.7	94.95611
5 G		10/02/2023	7278620	357.5	7278262.5	102.05287
6 G		10/02/2023	7280820	357.5	7280462.5	102.08371
7 G		10/02/2023	7649660	357.5	7649302.5	107.25544
8 G		10/02/2023	7060490	357.5	7060132.5	98.99434
5 G		19/04/2024	7213680	254.5	7213425.5	109.62552
6 G		19/04/2024	6478530	254.5	6478275.5	98.45313

7	G		19/04/2024	6635090	254.5	6634835.5	100.83244
8	G		19/04/2024	5823810	254.5	5823555.5	88.50307
9	G	**Positive Control**	03/02/2023	24098	310.0	23787.8	0.26329
10	G		03/02/2023	13920	310.0	13609.9	0.15064
11	G		03/02/2023	11457	310.0	11147.4	0.12338
12	G		03/02/2023	8191	310.0	7880.7	0.08723
9	G		08/02/2023	15446	2661.3	12784.5	0.16395
10	G		08/02/2023	10861	2661.3	8199.5	0.10515
11	G		08/02/2023	17599	2661.3	14937.4	0.19156
12	G		08/02/2023	15266	2661.3	12604.2	0.16164
9	G		10/02/2023	12949	357.5	12591.4	0.17655
10	G		10/02/2023	26320	357.5	25962.9	0.36404
11	G		10/02/2023	17344	357.5	16986.8	0.23818
12	G		10/02/2023	12428	357.5	12070.8	0.16925
9	G		19/04/2024	18819	254.5	18564.0	0.28213
10	G		19/04/2024	25549	254.5	25294.2	0.38441
11	G		19/04/2024	21092	254.5	20837.7	0.31668
12	G		19/04/2024	21737	254.5	21482.9	0.32648

KSL-W tested on L929 in the presence of FBS

Well	Row	KSL-W [µg/mL]	Date	Sample (RLU)	Blank (RLU)	Sample-Blank	Cell activity (%)
1	D	450.0	03/02/2023	5907	498.0	5409.4	0.04426
1	E	450.0	03/02/2023	2990	498.0	2492.4	0.02039
1	F	450.0	03/02/2023	6760	498.0	6261.9	0.05124
2	D	225.0	03/02/2023	15728	498.0	15230.1	0.12462

2	E	225.0	03/02/2023	32279	498.0	31780.7	0.26003
2	F	225.0	03/02/2023	10300	498.0	9802.3	0.08020
3	D	112.5	03/02/2023	11810100	498.0	11809602.0	96.62813
3	E	112.5	03/02/2023	12333100	498.0	12332602.0	100.90740
3	F	112.5	03/02/2023	10840300	498.0	10839802.0	88.69306
4	D	56.3	03/02/2023	13416400	498.0	13415902.0	109.77114
4	E	56.3	03/02/2023	13792500	498.0	13792002.0	112.84846
4	F	56.3	03/02/2023	13581300	498.0	13580802.0	111.12038
5	D	28.1	03/02/2023	14151800	498.0	14151302.0	115.78831
5	E	28.1	03/02/2023	13502500	498.0	13502002.0	110.47563
5	F	28.1	03/02/2023	13655600	498.0	13655102.0	111.72832
6	D	14.1	03/02/2023	13772000	498.0	13771502.0	112.68072
6	E	14.1	03/02/2023	12956700	498.0	12956202.0	106.00980
6	F	14.1	03/02/2023	12882000	498.0	12881502.0	105.39859
7	D	7.0	03/02/2023	13603100	498.0	13602602.0	111.29875
7	E	7.0	03/02/2023	12660100	498.0	12659602.0	103.58297
7	F	7.0	03/02/2023	13840100	498.0	13839602.0	113.23793
8	D	3.5	03/02/2023	14094500	498.0	14094002.0	115.31947
8	E	3.5	03/02/2023	13659800	498.0	13659302.0	111.76268
8	F	3.5	03/02/2023	13889100	498.0	13888602.0	113.63885
9	D	1.8	03/02/2023	13298500	498.0	13298002.0	108.80647
9	E	1.8	03/02/2023	12717700	498.0	12717202.0	104.05426
9	F	1.8	03/02/2023	12987900	498.0	12987402.0	106.26508
10	D	0.9	03/02/2023	14178300	498.0	14177802.0	116.00514
10	E	0.9	03/02/2023	13622600	498.0	13622102.0	111.45831
10	F	0.9	03/02/2023	12873500	498.0	12873002.0	105.32905
11	D	0.4	03/02/2023	13416700	498.0	13416202.0	109.77360
11	E	0.4	03/02/2023	12910500	498.0	12910002.0	105.63179

11	F	0.4	03/02/2023	13723600	498.0	13723102.0	112.28470
12	D	0.2	03/02/2023	13375000	498.0	13374502.0	109.43240
12	E	0.2	03/02/2023	12415100	498.0	12414602.0	101.57834
12	F	0.2	03/02/2023	13086400	498.0	13085902.0	107.07103
9	H	Blank	03/02/2023	1040			
10	H	Blank	03/02/2023	270			
11	H	Blank	03/02/2023	340			
12	H	Blank	03/02/2023	342			
1	D	450	08/02/2023	7652	538.0	7114.4	0.05435
1	E	450	08/02/2023	5311	538.0	4773.1	0.03646
1	F	450	08/02/2023	9476	538.0	8937.7	0.06828
2	D	225	08/02/2023	37520	538.0	36981.6	0.28252
2	E	225	08/02/2023	40214	538.0	39676.2	0.30311
2	F	225	08/02/2023	19616	538.0	19077.8	0.14575
3	D	112.5	08/02/2023	13192700	538.0	13192162.0	100.78248
3	E	112.5	08/02/2023	12910900	538.0	12910362.0	98.62965
3	F	112.5	08/02/2023	13556800	538.0	13556262.0	103.56405
4	D	56.25	08/02/2023	14500900	538.0	14500362.0	110.77657
4	E	56.25	08/02/2023	13359900	538.0	13359362.0	102.05982
4	F	56.25	08/02/2023	14869200	538.0	14868662.0	113.59023
5	D	28.1	08/02/2023	14709600	538.0	14709062.0	112.37095
5	E	28.1	08/02/2023	14143400	538.0	14142862.0	108.04543
5	F	28.1	08/02/2023	14709400	538.0	14708862.0	112.36942
6	D	14.1	08/02/2023	14183500	538.0	14182962.0	108.35177
6	E	14.1	08/02/2023	13451500	538.0	13450962.0	102.75960
6	F	14.1	08/02/2023	14886600	538.0	14886062.0	113.72316
7	D	7.0	08/02/2023	13999400	538.0	13998862.0	106.94533
7	E	7.0	08/02/2023	13955000	538.0	13954462.0	106.60613

7	F	7.0	08/02/2023	14718500	538.0	14717962.0	112.43894
8	D	3.5	08/02/2023	14525900	538.0	14525362.0	110.96756
8	E	3.5	08/02/2023	14153900	538.0	14153362.0	108.12564
8	F	3.5	08/02/2023	15300100	538.0	15299562.0	116.88212
9	D	1.8	08/02/2023	13715200	538.0	13714662.0	104.77416
9	E	1.8	08/02/2023	13878800	538.0	13878262.0	106.02399
9	F	1.8	08/02/2023	14266000	538.0	14265462.0	108.98204
10	D	0.9	08/02/2023	13633300	538.0	13632762.0	104.14848
10	E	0.9	08/02/2023	14419300	538.0	14418762.0	110.15318
10	F	0.9	08/02/2023	13904800	538.0	13904262.0	106.22262
11	D	0.4	08/02/2023	13782100	538.0	13781562.0	105.28525
11	E	0.4	08/02/2023	14161700	538.0	14161162.0	108.18523
11	F	0.4	08/02/2023	13582000	538.0	13581462.0	103.75657
12	D	0.2	08/02/2023	13609300	538.0	13608762.0	103.96513
12	E	0.2	08/02/2023	13721100	538.0	13720562.0	104.81923
12	F	0.2	08/02/2023	14074700	538.0	14074162.0	107.52059
9	H	Blank	08/02/2023	1152			
10	H	Blank	08/02/2023	296			
11	H	Blank	08/02/2023	296			
12	H	Blank	08/02/2023	408			
1	D	450	10/02/2023	4683	559.0	4123.9	0.02998
1	E	450	10/02/2023	1700	559.0	1141.1	0.00829
1	F	450	10/02/2023	4643	559.0	4083.9	0.02968
2	D	225	10/02/2023	11774	559.0	11214.7	0.08152
2	E	225	10/02/2023	6866	559.0	6306.9	0.04584
2	F	225	10/02/2023	15252	559.0	14692.5	0.10680
3	D	112.5	10/02/2023	12910700	559.0	12910141.0	93.84098
3	E	112.5	10/02/2023	12628900	559.0	12628341.0	91.79264

3	F	112.5	10/02/2023	12411700	559.0	12411141.0	90.21386
4	D	56.25	10/02/2023	14188000	559.0	14187441.0	103.12539
4	E	56.25	10/02/2023	14575800	559.0	14575241.0	105.94423
4	F	56.25	10/02/2023	14153500	559.0	14152941.0	102.87462
5	D	28.1	10/02/2023	14465400	559.0	14464841.0	105.14175
5	E	28.1	10/02/2023	14797000	559.0	14796441.0	107.55208
5	F	28.1	10/02/2023	14954200	559.0	14953641.0	108.69473
6	D	14.1	10/02/2023	15346000	559.0	15345441.0	111.54264
6	E	14.1	10/02/2023	14719700	559.0	14719141.0	106.99020
6	F	14.1	10/02/2023	15205100	559.0	15204541.0	110.51847
7	D	7.0	10/02/2023	14350000	559.0	14349441.0	104.30294
7	E	7.0	10/02/2023	14680600	559.0	14680041.0	106.70600
7	F	7.0	10/02/2023	14969300	559.0	14968741.0	108.80449
8	D	3.5	10/02/2023	15145500	559.0	15144941.0	110.08525
8	E	3.5	10/02/2023	14487300	559.0	14486741.0	105.30094
8	F	3.5	10/02/2023	14688200	559.0	14687641.0	106.76124
9	D	1.8	10/02/2023	14706300	559.0	14705741.0	106.89280
9	E	1.8	10/02/2023	14782700	559.0	14782141.0	107.44814
9	F	1.8	10/02/2023	14685000	559.0	14684441.0	106.73798
10	D	0.9	10/02/2023	14963000	559.0	14962441.0	108.75870
10	E	0.9	10/02/2023	14915200	559.0	14914641.0	108.41125
10	F	0.9	10/02/2023	14093400	559.0	14092841.0	102.43777
11	D	0.4	10/02/2023	14657700	559.0	14657141.0	106.53954
11	E	0.4	10/02/2023	14299100	559.0	14298541.0	103.93296
11	F	0.4	10/02/2023	14699300	559.0	14698741.0	106.84192
12	D	0.2	10/02/2023	14713600	559.0	14713041.0	106.94586
12	E	0.2	10/02/2023	14351800	559.0	14351241.0	104.31602
12	F	0.2	10/02/2023	14929100	559.0	14928541.0	108.51229

9	H	Blank	10/02/2023	1182			
10	H	Blank	10/02/2023	264			
11	H	Blank	10/02/2023	286			
12	H	Blank	10/02/2023	504			
1	G	**Reference Control**	03/02/2023	11310400	498.0	11309902.0	92.53950
2	G		03/02/2023	11959100	498.0	11958602.0	97.84727
3	G		03/02/2023	12523500	498.0	12523002.0	102.46529
4	G		03/02/2023	13095800	498.0	13095302.0	107.14794
1	G		08/02/2023	11525200	538.0	11524662.0	88.04350
2	G		08/02/2023	13004100	538.0	13003562.0	99.34166
3	G		08/02/2023	14083200	538.0	14082662.0	107.58552
4	G		08/02/2023	13748600	538.0	13748062.0	105.02932
1	G		10/02/2023	13761200	559.0	13760641.0	100.02308
2	G		10/02/2023	13552800	559.0	13552241.0	98.50826
3	G		10/02/2023	13844700	559.0	13844141.0	100.63002
4	G		10/02/2023	13873400	559.0	13872841.0	100.83864
5	G	**Medium Control**	03/02/2023	12525400	498.0	12524902.0	102.48083
6	G		03/02/2023	12654100	498.0	12653602.0	103.53388
7	G		03/02/2023	12642000	498.0	12641502.0	103.43487
8	G		03/02/2023	12273500	498.0	12273002.0	100.41975
5	G		08/02/2023	13769700	538.0	13769162.0	105.19052
6	G		08/02/2023	13893500	538.0	13892962.0	106.13630
7	G		08/02/2023	13704400	538.0	13703862.0	104.69165
8	G		08/02/2023	14693900	538.0	14693362.0	112.25101
5	G		10/02/2023	13681200	559.0	13680641.0	99.44158
6	G		10/02/2023	14123700	559.0	14123141.0	102.65801
7	G		10/02/2023	14947500	559.0	14946941.0	108.64603

8	G		10/02/2023	14410800	559.0	14410241.0	104.74488
9	G	**Positive Control**	03/02/2023	7977	498.0	7478.6	0.06119
10	G		03/02/2023	5337	498.0	4839.2	0.03959
11	G		03/02/2023	5737	498.0	5239.3	0.04287
12	G		03/02/2023	4309	498.0	3810.7	0.03118
9	G		08/02/2023	8833	538.0	8295.2	0.06337
10	G		08/02/2023	5761	538.0	5223.3	0.03990
11	G		08/02/2023	5987	538.0	5449.5	0.04163
12	G		08/02/2023	4553	538.0	4014.8	0.03067
9	G		10/02/2023	9612	559.0	9052.8	0.06580
10	G		10/02/2023	6236	559.0	5676.6	0.04126
11	G		10/02/2023	6690	559.0	6130.8	0.04456
12	G		10/02/2023	4781	559.0	4221.9	0.03069

KSL-W tested on L929 in the absence of FBS

Well	Row	KSL-W [µg/mL]	Date	Sample (RLU)	Blank (RLU)	Sample-Blank	Cell activity (%)
1	D	450.0	15/02/2023	54265	260.5	54004.0	0.74777
1	E	450.0	15/02/2023	39147	260.5	38886.2	0.53844
1	F	450.0	15/02/2023	44150	260.5	43889.3	0.60772
2	D	225.0	15/02/2023	41045	260.5	40784.5	0.56472
2	E	225.0	15/02/2023	27533	260.5	27272.5	0.37763
2	F	225.0	15/02/2023	38230	260.5	37969.3	0.52574
3	D	112.5	15/02/2023	839388	260.5	839127.5	11.61901
3	E	112.5	15/02/2023	944262	260.5	944001.5	13.07116
3	F	112.5	15/02/2023	1082480	260.5	1082219.5	14.98500

4	D	56.3	15/02/2023	9508270	260.5	9508009.5	131.65306
4	E	56.3	15/02/2023	10543600	260.5	10543339.5	145.98880
4	F	56.3	15/02/2023	8486600	260.5	8486339.5	117.50646
5	D	28.1	15/02/2023	9445730	260.5	9445469.5	130.78709
5	E	28.1	15/02/2023	9921630	260.5	9921369.5	137.37666
5	F	28.1	15/02/2023	8589200	260.5	8588939.5	118.92712
6	D	14.1	15/02/2023	9830870	260.5	9830609.5	136.11995
6	E	14.1	15/02/2023	11205700	260.5	11205439.5	155.15659
6	F	14.1	15/02/2023	9057650	260.5	9057389.5	125.41353
7	D	7.0	15/02/2023	10216400	260.5	10216139.5	141.45821
7	E	7.0	15/02/2023	10085800	260.5	10085539.5	139.64985
7	F	7.0	15/02/2023	10238900	260.5	10238639.5	141.76976
8	D	3.5	15/02/2023	10070600	260.5	10070339.5	139.43938
8	E	3.5	15/02/2023	11660500	260.5	11660239.5	161.45400
8	F	3.5	15/02/2023	10067500	260.5	10067239.5	139.39646
9	D	1.8	15/02/2023	9990950	260.5	9990689.5	138.33651
9	E	1.8	15/02/2023	10555800	260.5	10555539.5	146.15773
9	F	1.8	15/02/2023	9870310	260.5	9870049.5	136.66606
10	D	0.9	15/02/2023	10521800	260.5	10521539.5	145.68694
10	E	0.9	15/02/2023	11087700	260.5	11087439.5	153.52270
10	F	0.9	15/02/2023	11506200	260.5	11505939.5	159.31748
11	D	0.4	15/02/2023	10591200	260.5	10590939.5	146.64789
11	E	0.4	15/02/2023	10877000	260.5	10876739.5	150.60524
11	F	0.4	15/02/2023	10287900	260.5	10287639.5	142.44824
12	D	0.2	15/02/2023	9867710	260.5	9867449.5	136.63006
12	E	0.2	15/02/2023	10929700	260.5	10929439.5	151.33495
12	F	0.2	15/02/2023	11032800	260.5	11032539.5	152.76253
9	H	Blank	15/02/2023	486			

10	H	Blank	15/02/2023	114			
11	H	Blank	15/02/2023	110			
12	H	Blank	15/02/2023	332			
1	D	450	17/02/2023	49448	231.5	49216.6	0.60662
1	E	450	17/02/2023	43726	231.5	43494.7	0.53609
1	F	450	17/02/2023	39199	231.5	38967.4	0.48029
2	D	225	17/02/2023	34491	231.5	34259.2	0.42226
2	E	225	17/02/2023	30256	231.5	30024.0	0.37006
2	F	225	17/02/2023	27216	231.5	26984.8	0.33260
3	D	112.5	17/02/2023	1318720	231.5	1318488.5	16.25101
3	E	112.5	17/02/2023	1959660	231.5	1959428.5	24.15090
3	F	112.5	17/02/2023	836515	231.5	836283.5	10.30760
4	D	56.25	17/02/2023	8036920	231.5	8036688.5	99.05605
4	E	56.25	17/02/2023	8897290	231.5	8897058.5	109.66053
4	F	56.25	17/02/2023	8265050	231.5	8264818.5	101.86787
5	D	28.1	17/02/2023	6875400	231.5	6875168.5	84.73976
5	E	28.1	17/02/2023	8665380	231.5	8665148.5	106.80212
5	F	28.1	17/02/2023	7668700	231.5	7668468.5	94.51756
6	D	14.1	17/02/2023	8250020	231.5	8249788.5	101.68261
6	E	14.1	17/02/2023	9518900	231.5	9518668.5	117.32217
6	F	14.1	17/02/2023	7976680	231.5	7976448.5	98.31357
7	D	7.0	17/02/2023	10193200	231.5	10192968.5	125.63324
7	E	7.0	17/02/2023	8703130	231.5	8702898.5	107.26741
7	F	7.0	17/02/2023	8357750	231.5	8357518.5	103.01044
8	D	3.5	17/02/2023	8551110	231.5	8550878.5	105.39369
8	E	3.5	17/02/2023	7921230	231.5	7920998.5	97.63012
8	F	3.5	17/02/2023	8502850	231.5	8502618.5	104.79886
9	D	1.8	17/02/2023	8318610	231.5	8318378.5	102.52802

9	E	1.8	17/02/2023	7968220	231.5	7967988.5	98.20929
9	F	1.8	17/02/2023	8608280	231.5	8608048.5	106.09834
10	D	0.9	17/02/2023	8913170	231.5	8912938.5	109.85626
10	E	0.9	17/02/2023	8707100	231.5	8706868.5	107.31634
10	F	0.9	17/02/2023	8476880	231.5	8476648.5	104.47877
11	D	0.4	17/02/2023	9071220	231.5	9070988.5	111.80430
11	E	0.4	17/02/2023	8809150	231.5	8808918.5	108.57416
11	F	0.4	17/02/2023	8994290	231.5	8994058.5	110.85610
12	D	0.2	17/02/2023	9122050	231.5	9121818.5	112.43080
12	E	0.2	17/02/2023	8091590	231.5	8091358.5	99.72989
12	F	0.2	17/02/2023	9208150	231.5	9207918.5	113.49203
9	H	Blank	17/02/2023	472			
10	H	Blank	17/02/2023	120			
11	H	Blank	17/02/2023	86			
12	H	Blank	17/02/2023	248			
1	D	450	22/02/2023	48285	333.0	47952.4	0.57081
1	E	450	22/02/2023	45222	333.0	44888.7	0.53434
1	F	450	22/02/2023	47741	333.0	47408.3	0.56433
2	D	225	22/02/2023	36300	333.0	35966.9	0.42814
2	E	225	22/02/2023	32561	333.0	32228.4	0.38363
2	F	225	22/02/2023	39115	333.0	38781.6	0.46164
3	D	112.5	22/02/2023	2330750	333.0	2330417.0	27.74040
3	E	112.5	22/02/2023	2276720	333.0	2276387.0	27.09725
3	F	112.5	22/02/2023	1469080	333.0	1468747.0	17.48341
4	D	56.25	22/02/2023	8290490	333.0	8290157.0	98.68289
4	E	56.25	22/02/2023	8818850	333.0	8818517.0	104.97229
4	F	56.25	22/02/2023	8955690	333.0	8955357.0	106.60118
5	D	28.1	22/02/2023	8958150	333.0	8957817.0	106.63047

5	E	28.1	22/02/2023	8699590	333.0	8699257.0	103.55267
5	F	28.1	22/02/2023	9200630	333.0	9200297.0	109.51686
6	D	14.1	22/02/2023	8773560	333.0	8773227.0	104.43318
6	E	14.1	22/02/2023	8762600	333.0	8762267.0	104.30271
6	F	14.1	22/02/2023	9745230	333.0	9744897.0	115.99957
7	D	7.0	22/02/2023	8407230	333.0	8406897.0	100.07252
7	E	7.0	22/02/2023	8824030	333.0	8823697.0	105.03395
7	F	7.0	22/02/2023	9488690	333.0	9488357.0	112.94581
8	D	3.5	22/02/2023	8997800	333.0	8997467.0	107.10244
8	E	3.5	22/02/2023	9206370	333.0	9206037.0	109.58518
8	F	3.5	22/02/2023	9545030	333.0	9544697.0	113.61646
9	D	1.8	22/02/2023	10057200	333.0	10056867.0	119.71314
9	E	1.8	22/02/2023	9226360	333.0	9226027.0	109.82314
9	F	1.8	22/02/2023	9513690	333.0	9513357.0	113.24340
10	D	0.9	22/02/2023	8415670	333.0	8415337.0	100.17299
10	E	0.9	22/02/2023	10107200	333.0	10106867.0	120.30832
10	F	0.9	22/02/2023	9414880	333.0	9414547.0	112.06721
11	D	0.4	22/02/2023	9527690	333.0	9527357.0	113.41005
11	E	0.4	22/02/2023	9892550	333.0	9892217.0	117.75321
11	F	0.4	22/02/2023	10117500	333.0	10117167.0	120.43093
12	D	0.2	22/02/2023	9517260	333.0	9516927.0	113.28590
12	E	0.2	22/02/2023	9764650	333.0	9764317.0	116.23074
12	F	0.2	22/02/2023	10148000	333.0	10147667.0	120.79399
9	H	Blank	22/02/2023	710			
10	H	Blank	22/02/2023	154			
11	H	Blank	22/02/2023	144			
12	H	Blank	22/02/2023	324			
1	G	**Reference Control**	15/02/2023	6599570	260.5	6599309.5	91.37762

2	G		15/02/2023	7127740	260.5	7127479.5	98.69095
3	G		15/02/2023	6915800	260.5	6915539.5	95.75631
4	G		15/02/2023	8246010	260.5	8245749.5	114.17512
1	G		17/02/2023	7941030	231.5	7940798.5	97.87416
2	G		17/02/2023	8311510	231.5	8311278.5	102.44051
3	G		17/02/2023	8386730	231.5	8386498.5	103.36763
4	G		17/02/2023	7814750	231.5	7814518.5	96.31770
1	G		22/02/2023	7541020	333.0	7540687.0	89.76149
2	G		22/02/2023	8662790	333.0	8662457.0	103.11461
3	G		22/02/2023	9124790	333.0	9124457.0	108.61409
4	G		22/02/2023	8275950	333.0	8275617.0	98.50982
5	G	**Medium Control**	15/02/2023	5776650	260.5	5776389.5	79.98302
6	G		15/02/2023	6016700	260.5	6016439.5	83.30689
7	G		15/02/2023	5762360	260.5	5762099.5	79.78516
8	G		15/02/2023	5864800	260.5	5864539.5	81.20360
5	G		17/02/2023	7051710	231.5	7051478.5	86.91287
6	G		17/02/2023	7333650	231.5	7333418.5	90.38791
7	G		17/02/2023	7036480	231.5	7036248.5	86.72515
8	G		17/02/2023	6532390	231.5	6532158.5	80.51200
5	G		22/02/2023	8107880	333.0	8107547.0	96.50917
6	G		22/02/2023	8152610	333.0	8152277.0	97.04162
7	G		22/02/2023	8303890	333.0	8303557.0	98.84240
8	G		22/02/2023	8722690	333.0	8722357.0	103.82764
9	G	**Positive Control**	15/02/2023	18097	260.5	17836.9	0.24698
10	G		15/02/2023	16355	260.5	16094.5	0.22285
11	G		15/02/2023	10178	260.5	9917.7	0.13733

12	G		15/02/2023	7604	260.5	7343.9	0.10169
9	G		17/02/2023	11305	231.5	11073.7	0.13649
10	G		17/02/2023	11445	231.5	11213.9	0.13822
11	G		17/02/2023	8805	231.5	8573.7	0.10567
12	G		17/02/2023	7548	231.5	7316.8	0.09018
9	G		22/02/2023	14869	333.0	14536.1	0.17303
10	G		22/02/2023	13431	333.0	13098.4	0.15592
11	G		22/02/2023	12404	333.0	12071.3	0.14369
12	G		22/02/2023	7768	333.0	7435.5	0.08851

KSL-W tested on hMSCs in the presence of FBS

Well	Row	KSL-W [µg/mL]	Date	Sample (RLU)	Blank (RLU)	Sample-Blank	Cell activity (%)
1	D	450.0	24/05/2023	2998	568.0	2430.4	0.04134
1	E	450.0	24/05/2023	2584	568.0	2016.3	0.03430
1	F	450.0	24/05/2023	4093	568.0	3524.7	0.05996
2	D	225.0	24/05/2023	19562	568.0	18993.7	0.32311
2	E	225.0	24/05/2023	14238	568.0	13670.3	0.23255
2	F	225.0	24/05/2023	15856	568.0	15288.3	0.26008
3	D	112.5	24/05/2023	3280180	568.0	3279612.0	55.79114
3	E	112.5	24/05/2023	2417820	568.0	2417252.0	41.12110
3	F	112.5	24/05/2023	2274330	568.0	2273762.0	38.68011
4	D	56.3	24/05/2023	5763840	568.0	5763272.0	98.04193
4	E	56.3	24/05/2023	5352940	568.0	5352372.0	91.05191
4	F	56.3	24/05/2023	6120530	568.0	6119962.0	104.10977
5	D	28.1	24/05/2023	5882690	568.0	5882122.0	100.06375

5	E	28.1	24/05/2023	6248350	568.0	6247782.0	106.28418
5	F	28.1	24/05/2023	6497180	568.0	6496612.0	110.51715
6	D	14.1	24/05/2023	5906190	568.0	5905622.0	100.46352
6	E	14.1	24/05/2023	5982030	568.0	5981462.0	101.75367
6	F	14.1	24/05/2023	6621370	568.0	6620802.0	112.62981
7	D	7.0	24/05/2023	6478310	568.0	6477742.0	110.19614
7	E	7.0	24/05/2023	6568680	568.0	6568112.0	111.73347
7	F	7.0	24/05/2023	6568780	568.0	6568212.0	111.73517
8	D	3.5	24/05/2023	6196660	568.0	6196092.0	105.40485
8	E	3.5	24/05/2023	6351340	568.0	6350772.0	108.03619
8	F	3.5	24/05/2023	6618940	568.0	6618372.0	112.58847
9	D	1.8	24/05/2023	6304480	568.0	6303912.0	107.23903
9	E	1.8	24/05/2023	6865660	568.0	6865092.0	116.78555
9	F	1.8	24/05/2023	6289060	568.0	6288492.0	106.97672
10	D	0.9	24/05/2023	5817100	568.0	5816532.0	98.94797
10	E	0.9	24/05/2023	5905000	568.0	5904432.0	100.44328
10	F	0.9	24/05/2023	6138530	568.0	6137962.0	104.41597
11	D	0.4	24/05/2023	5561620	568.0	5561052.0	94.60187
11	E	0.4	24/05/2023	5611760	568.0	5611192.0	95.45482
11	F	0.4	24/05/2023	5834260	568.0	5833692.0	99.23988
12	D	0.2	24/05/2023	4708350	568.0	4707782.0	80.08646
12	E	0.2	24/05/2023	5062690	568.0	5062122.0	86.11432
12	F	0.2	24/05/2023	5418350	568.0	5417782.0	92.16463
9	H	Blank	24/05/2024	920			
10	H	Blank	24/05/2025	404			
11	H	Blank	24/05/2026	336			
12	H	Blank	24/05/2027	612			
1	D	450	25/05/2023	2620	462.0	2158.3	0.04454

1	E	450	25/05/2023	2382	462.0	1920.2	0.03963
1	F	450	25/05/2023	3681	462.0	3218.6	0.06642
2	D	225	25/05/2023	19478	462.0	19015.5	0.39243
2	E	225	25/05/2023	19315	462.0	18853.3	0.38908
2	F	225	25/05/2023	16253	462.0	15790.8	0.32588
3	D	112.5	25/05/2023	2623200	462.0	2622738.0	54.12679
3	E	112.5	25/05/2023	2528310	462.0	2527848.0	52.16849
3	F	112.5	25/05/2023	1481690	462.0	1481228.0	30.56886
4	D	56.25	25/05/2023	5455130	462.0	5454668.0	112.57077
4	E	56.25	25/05/2023	5445420	462.0	5444958.0	112.37038
4	F	56.25	25/05/2023	5071720	462.0	5071258.0	104.65814
5	D	28.1	25/05/2023	5286610	462.0	5286148.0	109.09294
5	E	28.1	25/05/2023	5553440	462.0	5552978.0	114.59965
5	F	28.1	25/05/2023	5220100	462.0	5219638.0	107.72034
6	D	14.1	25/05/2023	5393440	462.0	5392978.0	111.29764
6	E	14.1	25/05/2023	5283650	462.0	5283188.0	109.03185
6	F	14.1	25/05/2023	5057110	462.0	5056648.0	104.35663
7	D	7.0	25/05/2023	5522950	462.0	5522488.0	113.97041
7	E	7.0	25/05/2023	5537840	462.0	5537378.0	114.27770
7	F	7.0	25/05/2023	5047680	462.0	5047218.0	104.16202
8	D	3.5	25/05/2023	5755160	462.0	5754698.0	118.76265
8	E	3.5	25/05/2023	5470470	462.0	5470008.0	112.88735
8	F	3.5	25/05/2023	5088760	462.0	5088298.0	105.00981
9	D	1.8	25/05/2023	5469040	462.0	5468578.0	112.85784
9	E	1.8	25/05/2023	5274470	462.0	5274008.0	108.84240
9	F	1.8	25/05/2023	5061990	462.0	5061528.0	104.45734
10	D	0.9	25/05/2023	5472070	462.0	5471608.0	112.92037
10	E	0.9	25/05/2023	5382500	462.0	5382038.0	111.07187

10	F	0.9	25/05/2023	5309670	462.0	5309208.0	109.56884
11	D	0.4	25/05/2023	5185070	462.0	5184608.0	106.99741
11	E	0.4	25/05/2023	5404070	462.0	5403608.0	111.51702
11	F	0.4	25/05/2023	4802790	462.0	4802328.0	99.10810
12	D	0.2	25/05/2023	4795350	462.0	4794888.0	98.95456
12	E	0.2	25/05/2023	4685670	462.0	4685208.0	96.69103
12	F	0.2	25/05/2023	4618450	462.0	4617988.0	95.30378
9	H	Blank	25/05/2024	684			
10	H	Blank	25/05/2025	312			
11	H	Blank	25/05/2026	400			
12	H	Blank	25/05/2027	452			
1	D	450	26/05/2023	9950	379.5	9570.6	0.15730
1	E	450	26/05/2023	3024	379.5	2644.9	0.04347
1	F	450	26/05/2023	4325	379.5	3945.3	0.06485
2	D	225	26/05/2023	17615	379.5	17235.2	0.28328
2	E	225	26/05/2023	24126	379.5	23746.3	0.39030
2	F	225	26/05/2023	23306	379.5	22926.8	0.37683
3	D	112.5	26/05/2023	3796240	379.5	3795860.5	62.38984
3	E	112.5	26/05/2023	3835850	379.5	3835470.5	63.04088
3	F	112.5	26/05/2023	3547950	379.5	3547570.5	58.30887
4	D	56.25	26/05/2023	6807680	379.5	6807300.5	111.88672
4	E	56.25	26/05/2023	6933400	379.5	6933020.5	113.95309
4	F	56.25	26/05/2023	6651760	379.5	6651380.5	109.32397
5	D	28.1	26/05/2023	6759880	379.5	6759500.5	111.10107
5	E	28.1	26/05/2023	6702310	379.5	6701930.5	110.15483
5	F	28.1	26/05/2023	7904870	379.5	7904490.5	129.92045
6	D	14.1	26/05/2023	6523140	379.5	6522760.5	107.20994
6	E	14.1	26/05/2023	6626000	379.5	6625620.5	108.90058

6	F	14.1	26/05/2023	6484060	379.5	6483680.5	106.56761
7	D	7.0	26/05/2023	6488240	379.5	6487860.5	106.63631
7	E	7.0	26/05/2023	6721710	379.5	6721330.5	110.47369
7	F	7.0	26/05/2023	6397890	379.5	6397510.5	105.15130
8	D	3.5	26/05/2023	6455640	379.5	6455260.5	106.10049
8	E	3.5	26/05/2023	6684510	379.5	6684130.5	109.86226
8	F	3.5	26/05/2023	6771670	379.5	6771290.5	111.29485
9	D	1.8	26/05/2023	6518560	379.5	6518180.5	107.13466
9	E	1.8	26/05/2023	6623080	379.5	6622700.5	108.85258
9	F	1.8	26/05/2023	6346520	379.5	6346140.5	104.30696
10	D	0.9	26/05/2023	6740330	379.5	6739950.5	110.77974
10	E	0.9	26/05/2023	5873240	379.5	5872860.5	96.52800
10	F	0.9	26/05/2023	6291420	379.5	6291040.5	103.40132
11	D	0.4	26/05/2023	6522900	379.5	6522520.5	107.20600
11	E	0.4	26/05/2023	6483550	379.5	6483170.5	106.55923
11	F	0.4	26/05/2023	6163290	379.5	6162910.5	101.29534
12	D	0.2	26/05/2023	6346760	379.5	6346380.5	104.31091
12	E	0.2	26/05/2023	6350290	379.5	6349910.5	104.36893
12	F	0.2	26/05/2023	6044720	379.5	6044340.5	99.34649
9	H	Blank	26/05/2024	790			
10	H	Blank	26/05/2025	204			
11	H	Blank	26/05/2026	242			
12	H	Blank	26/05/2027	282			
1	G	**Reference Control**	24/05/2023	5212640	568.0	5212072.0	88.66519
2	G		24/05/2023	5780970	568.0	5780402.0	98.33334
3	G		24/05/2023	6308510	568.0	6307942.0	107.30759
4	G		24/05/2023	6213650	568.0	6213082.0	105.69388
1	G		25/05/2023	4242410	462.0	4241948.0	87.54325

2 G		25/05/2023	4760260	462.0	4759798.0	98.23038
3 G		25/05/2023	5185230	462.0	5184768.0	107.00071
4 G		25/05/2023	5196130	462.0	5195668.0	107.22566
1 G		26/05/2023	5703750	379.5	5703370.5	93.74221
2 G		26/05/2023	5958570	379.5	5958190.5	97.93051
3 G		26/05/2023	6230010	379.5	6229630.5	102.39197
4 G		26/05/2023	6445590	379.5	6445210.5	105.93531
5 G	**Medium Control**	24/05/2023	6663750	568.0	6663182.0	113.35076
6 G		24/05/2023	7895850	568.0	7895282.0	134.31063
7 G		24/05/2023	7013560	568.0	7012992.0	119.30155
8 G		24/05/2023	6883590	568.0	6883022.0	117.09057
5 G		25/05/2023	5357220	462.0	5356758.0	110.55015
6 G		25/05/2023	5482410	462.0	5481948.0	113.13376
7 G		25/05/2023	5229450	462.0	5228988.0	107.91330
8 G		25/05/2023	5208520	462.0	5208058.0	107.48136
5 G		26/05/2023	6552200	379.5	6551820.5	107.68758
6 G		26/05/2023	6566500	379.5	6566120.5	107.92262
7 G		26/05/2023	6717030	379.5	6716650.5	110.39677
8 G		26/05/2023	6794730	379.5	6794350.5	111.67387
9 G	**Positive Control**	24/05/2023	5493	568.0	4925.2	0.08379
10 G		24/05/2023	3525	568.0	2956.5	0.05029
11 G		24/05/2023	3216	568.0	2648.4	0.04505
12 G		24/05/2023	2658	568.0	2090.3	0.03556
9 G		25/05/2023	3891	462.0	3428.6	0.07076
10 G		25/05/2023	2614	462.0	2152.3	0.04442
11 G		25/05/2023	2526	462.0	2064.3	0.04260

12	G		25/05/2023	2058	462.0	1596.2	0.03294
9	G		26/05/2023	4823	379.5	4443.4	0.07303
10	G		26/05/2023	3146	379.5	2766.9	0.04548
11	G		26/05/2023	2908	379.5	2528.8	0.04156
12	G		26/05/2023	2166	379.5	1786.7	0.02937

KSL-W tested on hMSCs in the absence of FBS

Well	Row	KSL-W [µg/mL]	Date	Sample (RLU)	Blank (RLU)	Sample - Blank	Cell activity (%)
1	D	450.0	31/05/2023	1126	143.5	982.5	0.02034
1	E	450.0	31/05/2023	922	143.5	778.5	0.01612
1	F	450.0	31/05/2023	1600	143.5	1456.6	0.03016
2	D	225.0	31/05/2023	1530	143.5	1386.6	0.02871
2	E	225.0	31/05/2023	1212	143.5	1068.6	0.02212
2	F	225.0	31/05/2023	2786	143.5	2642.8	0.05472
3	D	112.5	31/05/2023	54029	143.5	53885.9	1.11567
3	E	112.5	31/05/2023	45318	143.5	45174.6	0.93531
3	F	112.5	31/05/2023	36019	143.5	35875.6	0.74278
4	D	56.3	31/05/2023	3319860	143.5	3319716.5	68.73231
4	E	56.3	31/05/2023	2264180	143.5	2264036.5	46.87522
4	F	56.3	31/05/2023	2336110	143.5	2335966.5	48.36448
5	D	28.1	31/05/2023	4285910	143.5	4285766.5	88.73367
5	E	28.1	31/05/2023	3784970	143.5	3784826.5	78.36207
5	F	28.1	31/05/2023	3609460	143.5	3609316.5	74.72826
6	D	14.1	31/05/2023	4086270	143.5	4086126.5	84.60027
6	E	14.1	31/05/2023	3999440	143.5	3999296.5	82.80252
6	F	14.1	31/05/2023	3845390	143.5	3845246.5	79.61302

7	D	7.0	31/05/2023	4204880	143.5	4204736.5	87.05600
7	E	7.0	31/05/2023	3877300	143.5	3877156.5	80.27370
7	F	7.0	31/05/2023	3640180	143.5	3640036.5	75.36430
8	D	3.5	31/05/2023	4148530	143.5	4148386.5	85.88932
8	E	3.5	31/05/2023	4093570	143.5	4093426.5	84.75141
8	F	3.5	31/05/2023	3899220	143.5	3899076.5	80.72753
9	D	1.8	31/05/2023	3761580	143.5	3761436.5	77.87780
9	E	1.8	31/05/2023	3942260	143.5	3942116.5	81.61865
9	F	1.8	31/05/2023	3770580	143.5	3770436.5	78.06414
10	D	0.9	31/05/2023	3486880	143.5	3486736.5	72.19033
10	E	0.9	31/05/2023	3546010	143.5	3545866.5	73.41458
10	F	0.9	31/05/2023	3449120	143.5	3448976.5	71.40854
11	D	0.4	31/05/2023	3521890	143.5	3521746.5	72.91519
11	D	0.4	31/05/2023	3560110	143.5	3559966.5	73.70651
11	E	0.4	31/05/2023	3263540	143.5	3263396.5	67.56624
12	F	0.2	31/05/2023	3253170	143.5	3253026.5	67.35154
12	D	0.2	31/05/2023	3146300	143.5	3146156.5	65.13887
12	E	0.2	31/05/2023	3193590	143.5	3193446.5	66.11798
9	H	Blank	31/05/2023	350			
10	H	Blank	31/05/2023	70			
11	H	Blank	31/05/2023	58			
12	H	Blank	31/05/2023	96			
1	D	450	01/06/2023	1404	138.0	1266.1	0.02797
1	E	450	01/06/2023	1642	138.0	1504.1	0.03323
1	F	450	01/06/2023	2698	138.0	2560.3	0.05657
2	D	225	01/06/2023	3266	138.0	3128.4	0.06912
2	E	225	01/06/2023	2420	138.0	2282.2	0.05042
2	F	225	01/06/2023	4017	138.0	3878.7	0.08569

3	D	112.5	01/06/2023	683616	138.0	683478.0	15.10068
3	E	112.5	01/06/2023	847851	138.0	847713.0	18.72927
3	F	112.5	01/06/2023	623912	138.0	623774.0	13.78159
4	D	56.25	01/06/2023	4588000	138.0	4587862.0	101.36369
4	E	56.25	01/06/2023	4103560	138.0	4103422.0	90.66053
4	F	56.25	01/06/2023	4510590	138.0	4510452.0	99.65340
5	D	28.1	01/06/2023	5533440	138.0	5533302.0	122.25213
5	E	28.1	01/06/2023	5620600	138.0	5620462.0	124.17783
5	F	28.1	01/06/2023	5715130	138.0	5714992.0	126.26637
6	D	14.1	01/06/2023	5699690	138.0	5699552.0	125.92524
6	E	14.1	01/06/2023	5325130	138.0	5324992.0	117.64975
6	F	14.1	01/06/2023	5378530	138.0	5378392.0	118.82957
7	D	7.0	01/06/2023	6112090	138.0	6111952.0	135.03676
7	E	7.0	01/06/2023	5491310	138.0	5491172.0	121.32132
7	F	7.0	01/06/2023	5674510	138.0	5674372.0	125.36892
8	D	3.5	01/06/2023	5740080	138.0	5739942.0	126.81761
8	E	3.5	01/06/2023	5696020	138.0	5695882.0	125.84415
8	F	3.5	01/06/2023	5720320	138.0	5720182.0	126.38104
9	D	1.8	01/06/2023	5455840	138.0	5455702.0	120.53765
9	E	1.8	01/06/2023	5475790	138.0	5475652.0	120.97842
9	F	1.8	01/06/2023	5695480	138.0	5695342.0	125.83222
10	D	0.9	01/06/2023	5558390	138.0	5558252.0	122.80337
10	E	0.9	01/06/2023	5514290	138.0	5514152.0	121.82903
10	F	0.9	01/06/2023	5163410	138.0	5163272.0	114.07673
11	D	0.4	01/06/2023	5460840	138.0	5460702.0	120.64812
11	D	0.4	01/06/2023	5484530	138.0	5484392.0	121.17152
11	E	0.4	01/06/2023	5211190	138.0	5211052.0	115.13238
12	F	0.2	01/06/2023	5293140	138.0	5293002.0	116.94297

12	D	0.2	01/06/2023	5105110	138.0	5104972.0	112.78866
12	E	0.2	01/06/2023	5069800	138.0	5069662.0	112.00852
9	F	Blank	01/06/2023	340			
10	D	Blank	01/06/2023	64			
11	E	Blank	01/06/2023	80			
12	F	Blank	01/06/2023	68			
1	D	450.0	15/06/2023	3889	196.5	3692.1	0.07206
1	E	450.0	15/06/2023	3931	196.5	3734.1	0.07288
1	F	450.0	15/06/2023	4357	196.5	4160.3	0.08120
2	D	225.0	15/06/2023	4035	196.5	3838.2	0.07491
2	E	225.0	15/06/2023	4271	196.5	4074.2	0.07952
2	F	225.0	15/06/2023	4807	196.5	4610.4	0.08998
3	D	112.5	15/06/2023	1014550	196.5	1014353.5	19.79707
3	E	112.5	15/06/2023	1117750	196.5	1117553.5	21.81122
3	F	112.5	15/06/2023	636799	196.5	636602.5	12.42453
4	D	56.3	15/06/2023	5956320	196.5	5956123.5	116.24526
4	E	56.3	15/06/2023	4948710	196.5	4948513.5	96.57980
4	F	56.3	15/06/2023	4909750	196.5	4909553.5	95.81942
5	D	28.1	15/06/2023	6531880	196.5	6531683.5	127.47843
5	E	28.1	15/06/2023	6381500	196.5	6381303.5	124.54347
5	F	28.1	15/06/2023	6063400	196.5	6063203.5	118.33513
6	D	14.1	15/06/2023	6439120	196.5	6438923.5	125.66804
6	E	14.1	15/06/2023	6251120	196.5	6250923.5	121.99885
6	F	14.1	15/06/2023	6010110	196.5	6009913.5	117.29508
7	D	7.0	15/06/2023	6311760	196.5	6311563.5	123.18236
7	E	7.0	15/06/2023	6082060	196.5	6081863.5	118.69932
7	F	7.0	15/06/2023	5710170	196.5	5709973.5	111.44117
8	D	3.5	15/06/2023	6632290	196.5	6632093.5	129.43812

8	E	3.5	15/06/2023	6399810	196.5	6399613.5	124.90082
8	F	3.5	15/06/2023	5739740	196.5	5739543.5	112.01828
9	D	1.8	15/06/2023	6019260	196.5	6019063.5	117.47366
9	E	1.8	15/06/2023	5537350	196.5	5537153.5	108.06825
9	F	1.8	15/06/2023	5203410	196.5	5203213.5	101.55077
10	D	0.9	15/06/2023	6412100	196.5	6411903.5	125.14069
10	E	0.9	15/06/2023	5928950	196.5	5928753.5	115.71108
10	F	0.9	15/06/2023	5677890	196.5	5677693.5	110.81116
11	D	0.4	15/06/2023	6333710	196.5	6333513.5	123.61076
11	D	0.4	15/06/2023	5828420	196.5	5828223.5	113.74904
11	E	0.4	15/06/2023	5531760	196.5	5531563.5	107.95915
12	F	0.2	15/06/2023	5575660	196.5	5575463.5	108.81594
12	D	0.2	15/06/2023	5303340	196.5	5303143.5	103.50109
12	E	0.2	15/06/2023	5540010	196.5	5539813.5	108.12017
9	F	Blank	15/06/2023	472			
10	D	Blank	15/06/2023	64			
11	E	Blank	15/06/2023	78			
12	F	Blank	15/06/2023	172			
1	D	450.0	22/06/2023	3050	167.5	2882.9	0.05062
1	E	450.0	22/06/2023	2548	167.5	2380.8	0.04181
1	F	450.0	22/06/2023	3717	167.5	3549.1	0.06232
2	D	225.0	22/06/2023	3402	167.5	3235.0	0.05681
2	E	225.0	22/06/2023	2718	167.5	2550.8	0.04479
2	F	225.0	22/06/2023	4917	167.5	4749.5	0.08340
3	D	112.5	22/06/2023	777495	167.5	777327.5	13.64974
3	E	112.5	22/06/2023	417707	167.5	417539.5	7.33192
3	F	112.5	22/06/2023	394033	167.5	393865.5	6.91621
4	D	56.3	22/06/2023	4828210	167.5	4828042.5	84.77958

4	E	56.3	22/06/2023	4714210	167.5	4714042.5	82.77776
4	F	56.3	22/06/2023	5000720	167.5	5000552.5	87.80883
5	D	28.1	22/06/2023	6219790	167.5	6219622.5	109.21548
5	E	28.1	22/06/2023	5581910	167.5	5581742.5	98.01442
5	F	28.1	22/06/2023	5712590	167.5	5712422.5	100.30914
6	D	14.1	22/06/2023	6592130	167.5	6591962.5	115.75371
6	E	14.1	22/06/2023	6354970	167.5	6354802.5	111.58922
6	F	14.1	22/06/2023	5937690	167.5	5937522.5	104.26186
7	D	7.0	22/06/2023	6746080	167.5	6745912.5	118.45704
7	E	7.0	22/06/2023	5979880	167.5	5979712.5	105.00271
7	F	7.0	22/06/2023	5912140	167.5	5911972.5	103.81320
8	D	3.5	22/06/2023	6844830	167.5	6844662.5	120.19108
8	E	3.5	22/06/2023	6240990	167.5	6240822.5	109.58775
8	F	3.5	22/06/2023	5961310	167.5	5961142.5	104.67662
9	D	1.8	22/06/2023	6495050	167.5	6494882.5	114.04900
9	E	1.8	22/06/2023	5574920	167.5	5574752.5	97.89168
9	F	1.8	22/06/2023	5941790	167.5	5941622.5	104.33385
10	D	0.9	22/06/2023	6503530	167.5	6503362.5	114.19791
10	E	0.9	22/06/2023	5926270	167.5	5926102.5	104.06132
10	F	0.9	22/06/2023	6096740	167.5	6096572.5	107.05475
11	D	0.4	22/06/2023	6313210	167.5	6313042.5	110.85592
11	D	0.4	22/06/2023	6220070	167.5	6219902.5	109.22040
11	E	0.4	22/06/2023	5735380	167.5	5735212.5	100.70933
12	F	0.2	22/06/2023	6342770	167.5	6342602.5	111.37499
12	D	0.2	22/06/2023	5572590	167.5	5572422.5	97.85077
12	E	0.2	22/06/2023	5240150	167.5	5239982.5	92.01318
9	F	Blank	22/06/2023	386			
10	D	Blank	22/06/2023	92			

11	E	Blank	22/06/2023	66			
12	F	Blank	22/06/2023	126			
1	G	**Reference Control**	31/05/2023	3488430	143.5	3488286.5	72.22243
2	G		31/05/2023	3775550	143.5	3775406.5	78.16704
3	G		31/05/2023	5396030	143.5	5395886.5	111.71789
4	G		31/05/2023	6660250	143.5	6660106.5	137.89264
1	G		01/06/2023	4134710	138.0	4134572.0	91.34875
2	G		01/06/2023	4589200	138.0	4589062.0	101.39020
3	G		01/06/2023	4789830	138.0	4789692.0	105.82290
4	G		01/06/2023	4591370	138.0	4591232.0	101.43815
1	G		15/06/2023	4703750	196.5	4703553.5	91.79894
2	G		15/06/2023	5092760	196.5	5092563.5	99.39122
3	G		15/06/2023	4979100	196.5	4978903.5	97.17292
4	G		15/06/2023	5720200	196.5	5720003.5	111.63692
1	G		22/06/2023	5599540	167.5	5599372.5	98.32400
2	G		22/06/2023	5649900	167.5	5649732.5	99.20832
3	G		22/06/2023	6079320	167.5	6079152.5	106.74886
4	G		22/06/2023	5451180	167.5	5451012.5	95.71883
5	G	**Medium Control**	31/05/2023	4130120	143.5	4129976.5	85.50815
6	G		31/05/2023	4410490	143.5	4410346.5	91.31301
7	G		31/05/2023	4465900	143.5	4465756.5	92.46023
8	G		31/05/2023	4311130	143.5	4310986.5	89.25583
5	G		01/06/2023	4603120	138.0	4602982.0	101.69775
6	G		01/06/2023	4259770	138.0	4259632.0	94.11181
7	G		01/06/2023	4525110	138.0	4524972.0	99.97421
8	G		01/06/2023	4704630	138.0	4704492.0	103.94050
5	G		15/06/2023	4634030	196.5	4633833.5	90.43822

6 G		15/06/2023	4643900	196.5	4643703.5	90.63085
7 G		15/06/2023	4578360	196.5	4578163.5	89.35171
8 G		15/06/2023	4483650	196.5	4483453.5	87.50326
5 G		22/06/2023	5983270	167.5	5983102.5	105.06223
6 G		22/06/2023	6192210	167.5	6192042.5	108.73118
7 G		22/06/2023	6010370	167.5	6010202.5	105.53811
8 G		22/06/2023	5805770	167.5	5805602.5	101.94537
9 G	**Positive Control**	31/05/2023	3212	143.5	3068.9	0.06354
10 G		31/05/2023	1948	143.5	1804.7	0.03736
11 G		31/05/2023	1968	143.5	1824.7	0.03778
12 G		31/05/2023	1504	143.5	1360.6	0.02817
9 G		01/06/2023	3781	138.0	3642.6	0.08048
10 G		01/06/2023	2526	138.0	2388.3	0.05277
11 G		01/06/2023	2806	138.0	2668.3	0.05895
12 G		01/06/2023	2248	138.0	2110.2	0.04662
9 G		15/06/2023	4083	196.5	3886.2	0.07585
10 G		15/06/2023	2814	196.5	2617.8	0.05109
11 G		15/06/2023	3140	196.5	2943.9	0.05746
12 G		15/06/2023	2424	196.5	2227.7	0.04348
9 G		22/06/2023	4927	167.5	4759.5	0.08358
10 G		22/06/2023	3300	167.5	3132.9	0.05501
11 G		22/06/2023	3537	167.5	3369.0	0.05916
12 G		22/06/2023	2750	167.5	2582.8	0.04535
